# Acknowledgment to the Reviewers of *Molecules* in 2022

**DOI:** 10.3390/molecules28031072

**Published:** 2023-01-20

**Authors:** Molecules Editorial Office

**Affiliations:** MDPI AG, St. Alban-Anlage 66, 4052 Basel, Switzerland

High-quality academic publishing is built on rigorous peer review. *Molecules* was able to uphold its high standards for published papers due to the outstanding efforts of our reviewers. Thanks to the efforts of our reviewers in 2022, the median time to first decision was 14 days and the median time to publication was 34 days. Regardless of whether the articles they examined were ultimately published, the editors would like to express their appreciation and thank the following reviewers for the time and dedication that they have shown *Molecules*:
A. K. M. Mominul IslamLin DuA. N. M. Mamun-Or-RashidLin JiangAabid Manzoor ShahLin LiAamir MirzaLin QiuAamod DesaiLin XiAaron MuthLin YuAaron NilsenLina BaranauskieneAarti BainsLina CossignaniAasif HelalLina GaoAbbas RezaeeLina T. Al KuryAbd El-Fatah AbomohraLinards KlavinsAbdallah IbrahimLincheng ZhouAbdalrhman MiladLinda D. StewartAbdel Majid A. AdamLinda LorenzAbdel-Basit Al-OdayniLinda ShimizuAbdelfattah El MoussaouiLindamir Hernandez PastoriniAbdelghafar Abu-ElsaoudLindiwe KhotsengAbdelghani BenyoucefLindon W. K. MoodieAbdelghani IddarLing LiuAbdelhakim BouyahyaLing TianAbdelilah BeljebbarLing WangAbdelkhaleq LegssyerLing YangAbdelkharim MadhiLinghong MengAbdellatif BoukirLingxia ZhaoAbdelnaser A. ElzaawelyLingxiao XieAbdel-Naser B. SingabLingzhi WangAbdelouahab BellouLinhan LinAbdelrahman ZkriaLinnan LiAbdelsamed I. ElshamyLinnet KristianAbdeslam AsehraouaLinsen QingAbdeslam MeniaiLionel Fernel GamarraAbdollah AllahverdiLionel GamarraAbdollah GhasemianLioubov Kiwi-MinskerAbdolreza HosseindoustLipin JiAbdorreza Mohammadi NafchiLiping ChenAbdou BoucekkineLiping DuAbdul Bari Bari ShahLiping LuoAbdul BasitLiqiang LuoAbdul Hakeem DeshmukhLisa MehlmannAbdul Kader Jailani AmirudeenLisa PilkingtonAbdul QayyumLisa-Maria CallAbdul RohmanLisard Iglesias CarresAbdul SadiqLisardo BoscaAbdul Samad MumtazLiu HongkaiAbdul SirajLiu QiangAbdullah A. Al-GhanayemLiubov N. SkrypnikAbdullah Al Hadi Ahmad FuaadLiudmil AntonovAbdullah AlanaziLiudmyla V. GarmanchukAbdullah AlotaibiLivia DonatiAbdullah F. AlAsmariLivia PatrascuAbdullah KhanLiviu GiurgiulescuAbdullah N. AlodhaybLivleen ShuklaAbdullah S. AlshammariLiwei D. GengAbdullah ShaitoLiwei YangAbdullah UlasLiwen MaAbdullatif Bin MuhsinahLiya LiAbdusalom SuleymanovLiya LiuAbeer A. SharfalddinLiyan YangAbel Bult-ItoLiyun SongAbhay K. PandeyLjiljana Damjanović-VasilićAbhichart KrissanaprasitLjiljana Mihajlović-LalićAbhijeet SinhaLjiljana PopovicAbhijit GhoshLjubica AndjelkovicAbhijit ShirkeLjubica Glavas-ObrovacAbhilasha Kandahalli Venkataranga NayakaLjubisa NikolicAbhimanyu DevLjubiša Šari$\ddot{c}$Abhinav JoshiLoana MussoAbhinaw KumarLochan SinghAbhirup PatraLoganathan VeeramuthuAbhisek BhattacharyaLoic LemonnierAbhishek BhattacharyaLok ShresthaAbhishek ChauhanLoka Raghu Kumar PenkeAbhishek K. SinghLong Chiau MingAbid Aslam MaanLong HanAbid UllahLong JiaoAbilash Valsala GopalakrishnanLong ShengAbimbola E. OluwalanaLong WuAbiodun Olagoke AdenijiLong Y. ChiangAbishek IyerLongfei YuanAbolfazl AlirezaluLonghai GuoAbraão Almeida SantosLonglong MaAbraham Loera MuroLongtao MaAbraham Madariaga-MazónLongteng TangAbraham Wall MedranoLore TroalenAbu Bakar Mohamad HafiziLoredana AlboniciAbu Hena Mostafa KamalLoredana DumitrascuAbu Naser Md Ahsanul HaqueLoredana Elena NitaAbul HossainLoredana Elena VijanAbul Kalam AzadLoredana MarcuAbul MallikLorelei Irina BrasoveanuAbuzer AliLorella PasquinucciAçelya YilmazerLorena Mara Alexandre E. SilvaAchille AntenucciLorena TuchscherrAchinta BordoloiLorena UrbanelliAda PopoloLorenza TrabalziniAdam BuckiLorenzo AntoniniAdam CudowskiLorenzo AvaldiAdam EkielskiLorenzo CapriniAdam GnatowskiLorenzo CorsiÁdám HaimhofferLorenzo GuazzelliAdam HermawanLorenzo MalavasiAdam HuczyńskiLorenzo MortaraAdam KlimowiczLorenzo SpadaAdam KowalczykLorenzo TassiAdam LesnerLorenzo TesiAdam LiwoLoreta Tamašauskaitė-TamašiūnaitėAdam MatkowskiLoris PintoAdam MieczkowskiLothar BreckerAdam PomorskiLouis E. N. JackaiAdam RomanLouis Kuoping ChaoAdam SajnógLouis Pergaud SandjoAdam SieradzanLouis V. AdriaenssensAdam SzewczykLouise BennetAdam T. SuttonLourdes Macías-RodríguezAdam WeitemierLowell D. KispertAdam ZmysłowskiLu LuAdan Pinto-FernandezLu ZhangAdawiya J. HaiderLu ZhengAdél LenLuan Luong ChuAdela PinteaLuba TchertanovAdelaide MirandaLuben N. ArnaudovAdelbert De ClercqĽubomír MedveckýAdele CarradoLubomira Nikolaeva-GlombAdele CutignanoLubos CipakAdele PapettiLuca BellucciAdelia AquinoLuca BursiAdelina Jiménez ArellanesLuca CiceroAdelino M. GalvãoLuca Domenico D'AndreaAderval S. LunaLuca FortiAdesh SainiLuca GonsalviAdi SikinLuca MoriniAdil AkkouchLuca PalazzoloAdil Shareef MohammedLuca PanarielloAdina Ionuta GavrilaLuca PinziAdina NegreaLuca PosaAdina RaducanLuca RosciniAdina SantanaLuca SancinetoAdina-Elena SegneanuLuca ScottiAditi JainLuca SilvestriAditya GaraiLuca ValgimigliAdnan AynaLuca VanellaAdnan KadiLucas Bragança CarvalhoAdnan KhanLucas D. DiasAdnan MujahidLucas Freitas De FreitasAdnan YounisLucas Hernández-SaraviaAdolf SandbichlerLucas MeiliAdolfo Andrade CettoLucélia HoehneAdolfo Paz SilvaLucia BalejčíkováAdrian AntosikLucia BattistiniAdrian ArendowskiLucia BurgioAdrián BenitoLucia CalucciAdrian Bogdan ȚiguLucia CarlucciAdrian BurlacuLucia Carmen TrincăAdrian Cǎtǎlin PuitelLucia D'AccoltiAdrian KellerLucia FagiolariAdrian Krzysztof AntosikLucia Maria Armelin-CorreaAdrián MatencioLucia Monica VecaAdrián RojasLucia MontenegroAdrian ȘpacLucia MorbidelliAdrian SturzaLucia RoccoAdrian Zaragoza-BastidaLucia SilvestriniAdriana ArigòLucian CopoloviciAdriana BakalovaLucian HriţcuAdriana ChilinLuciana BordinAdriana CiurbaLuciana Daniela LarioAdriana DescalzoLuciana Pereira RangelAdriana FilipLuciana ScottiAdriana FlachLuciana SpinelliAdriana HanganLucian-Gabriel ZamfirAdriana M. CalvoLuciano C. AlmeidaAdriana MorarLuciano De SioAdriana TarantinoLucie BrulíkováAdriana TrifanLucie HejnovaAdriana UrdăLucie Raisová StuchlíkováAdriane Bianchi Pedroni MedeirosLucien VelevaAdriano A. MendesLucila DoumicAdriano FischLucilla ParnettiAdriano Gomes Da CruzLucinda V. ReisAdriano MollicaLúcio Cardozo-FilhoAdriano TroiaLucjan StrekowskiAdrienne ScheckLucjusz ZaprutkoAdy GiordanoLucretia UdrescuAekkhaluck IntharuksaLucrezia SergioAfonso Caricati-NetoLudmila A. FrankAftab AlamLudmila L. SemenychevaAftab YaseenLudovic ColombeauAfzal Husain KhanLudovico MiglioloAfzal HussainLudovina GalegoAfzal KhanLuigi CampanellaAfzal ShaikLuigi MandrichAgata AntoniewskaLuigi MannaAgata Antosik-RogóżLuigi MenghiniAgata GórskaLuís Adriano Santos Do NascimentoAgata Jabłońska-TrypućLuis Alberto Cira ChávezAgata JędrzejukLuis Alfonso Trujillo-CayadoAgata Kot-WasikLuis AlvesAgata ŁamaczLuis Angel Medina JuarezAgata Motyka-PomagrukLuis Antônio EsmerinoAgata Trzęsowska-KruszyńskaLuis Argel PovedaAgata WawrzyńczakLuis BarbeitoAgata ZnamirowskaLuís C. N. Da SilvaAgatha BastidaLuis CáceresAgieshkumar Balakrishna PillaiLuís Carlos De MoraisAgileo Hernández-GordilloLuis Castro-SánchezAglaia PappaLuis Cláudio Nascimento Da SilvaAgnes CastilloLuis Cobos-PucÁgnes FarkasLuis Cubilla-RiosAgnès HagègeLuis Del Pozo-YaunerÁgnes KlusóczkiLuis Edmundo Lugo UribeAgnes PurwidyantriLuis Eduardo DíazÁgnes TelbiszLuis Enrique CucaAgnese De MarioLuis Exequiel IbarraAgnese GiacominoLuis Felipe GiraldoAgnieszka A. KaczorLuis Felipe Pérez-RomeroAgnieszka BossowskaLuis Fernando Magaña SolísAgnieszka CuprysLuis Filipe Baptista De GouveiaAgnieszka Dobrzyńska-IngerLuís Filipe TeixeiraAgnieszka DudziakLuís GalesAgnieszka Ewa Wia̧cekLuis García-ZapateiroAgnieszka FogelLuis GoyaAgnieszka Gadomska-GajadhurLuís Gustavo Romani FernandesAgnieszka GalantyLuis Jesús Villarreal-GómezAgnieszka Jagiello-GruszfeldLuis Menéndez-AriasAgnieszka JasińskaLuis Noguera ArtiagaAgnieszka JeleńLuís Pedro Ferreira RatoAgnieszka Kącka-ZychLuis PeñalozaAgnieszka KarbownikLuís PinhoAgnieszka KarczmarczykLuís Pinto Da SilvaAgnieszka KicelLuis Puente-DíazAgnieszka KierysLuis R. HernándezAgnieszka KorgaLuis Rivera GavidiaAgnieszka KowalczukLuis Roberto De Camargo GonçalvesAgnieszka Łapczuk-KrygierLuis RodrigoAgnieszka LaskowskaLuis VacaAgnieszka Łękawa-RausLuisa B. MaiaAgnieszka MarkowskaLuísa Correia-SáAgnieszka MroczekLuisa Maria FerreiraAgnieszka PilarskaLuisa Maria Hora De CarvalhoAgnieszka Pluta-KubicaLuisa PastiAgnieszka RichertLuiz Antonio Alcântara PereiraAgnieszka Skalska-KamińskaLuiz Felipe Domingues PasseroAgnieszka StawarskaLuiz Leonardo SaldanhaAgnieszka StolarczykLuiz PereiraAgnieszka SynowiecLuiza Rosaria Sousa DiasAgnieszka Tafelska-KaczmarekLujia LiuAgnieszka TorzewskaLujun PanAgnieszka Ulatowska-JarżaLukas HlebaAgnieszka ViapianaLukáš KubalaAgnieszka WdowiakLukas PfeiferAgnieszka WnukLukas RycekAgnieszka Zdybicka-BarabasLukáš SmolkoAgnieszka ZielińskaLukáš StřižíkAgnieszka ZwolińskaLukáš ŽídekAgostino CasapulloŁukasz BalewskiAgre PaterneŁukasz DąbrowskiAgris BerzinsŁukasz FijałkowskiAgueda MassaŁukasz HetmańczykAgustín AriñoŁukasz K. KaczyńskiAgustin R. González-ElipeŁukasz KomstaAgustina La-VeniaLukasz KozlowskiAharon AzaguryŁukasz KucharskiAhlam NematiŁukasz KuźmaAhmad Abd-El-AzizŁukasz ŁopusiewiczAhmad Adlie ShamsuriŁukasz SęczykAhmad Beng Hong KuehŁukasz SobottaAhmad Fahmi Lim AbdullahLukasz SojkaAhmad Fairuz OmarŁukasz StępieńAhmad G. HegaziŁukasz ŚwiątekAhmad Hussaini JagabaŁukasz SzczukowskiAhmad IrfanŁukasz SzeleszczukAhmad J. ObaidullahLuke HunterAhmad M. HarzandiLumei PuAhmad ManasrahLuminita CrisanAhmad Shukri Muhammad NoorLuna Maslov BandićAhmar RaufLuntao WangAhmed A. Al-KarmalawyLuqmanulhakim BaharudinAhmed A. ElhenawyLusheng FanAhmed A. ZakiLu-Sheng HsiehAhmed A. ZakyLusi ErnawatiAhmed Abdel FattahLuu Thai DanhAhmed AbdelwahabLuxin SunAhmed Al-JanabiLynda H. McCarthyAhmed Al-JumailiLyudmila DimitrovaAhmed Al-MamooriLyudmila M. LyalinaAhmed AlzamlyLyudmila ParfenovaAhmed E. Abdel MoneimM. Ali AboudzadehAhmed Eid AlprolM. ArulperumjothiAhmed ElbannaM. Gracia García-MartínAhmed El-FiqiM. JameelAhmed FatimiM. L. Almoraima GilAhmed GaberM. Quadir SiddiquiAhmed GabrM. Ramesan RekhaAhmed H. El-GhorabM. RameshAhmed HajibM. Shahnawaz KhanAhmed HasaballahM. Venkata KamalakarAhmed HashimMaaria KortesniemiAhmed HassanMaarit KaronenAhmed I. A. Abd El-MageedMaarten MeesAhmed KhodairMacarena Martínez-BailénAhmed M. Abu-DiefMachi KannaAhmed M. GoudaMaciej BalawejderAhmed M. NaglahMaciej ChotkowskiAhmed M. ZeitounMaciej JarzebskiAhmed MaghrabyMaciej KluzAhmed MedianiMaciej Łukasz DębowskiAhmed Mohamed Abdel-AzeemMaciej MaciejczykAhmed Mohammed Abu-Dief MohammedMaciej MalinowskiAhmed Mohsen Elsaid HamdanMaciej NastajAhmed MustafaMaciej PilarekAhmed N. BadrMaciej PrzybyłekAhmed NafisMaciej StrzemskiAhmed Nuri KursunluMaciej SzaleniecAhmed RagabMaciej ZaranekAhmed RakibMadalina PandeleAhmed S. El-ShafieMădălin-Iancu EnacheAhmed SayedMadan Kumar PerumalAhmed TawfikMadan VermaAhmed TemirakMaddaka ReddeppaAhmed WahidMaddalena ColliniAhmedova AnifeMadhavan NampoothiriAhmer SiyalMadhu KamleAhmet Emin TopalMadhu Sudhana SaddalaAhmet KertmenMadhulika PradhanAhmet SariMadoka SuzukiAhmet Sinan CevikMafalda AlmeidaAhsan HabibMagadi SrivathsaAida Mihaela VasileMagali GauthierAidilla MubarakMagda H. AbdellattifAikaterini K. AndreopoulouMagda JanalikovaAikaterini KoutsavitiMagdalena BartnikAikaterini MarkopoulouMagdalena BarwiolekAilton Cesar LemesMagdalena BrodaAimable NahimanaMagdalena CaraAinara SaralegiMagdalena Cristina StanciuAinhoa Plaza-ZabalaMagdalena DadanAirat KayumovMagdalena Długosz-LisieckaAirton DeppmanMagdalena Dziagwa-BeckerAirton Germano Bispo-JrMagdalena FabjanowiczAishwaryadev BanerjeeMagdalena Franczyk-ŻarówAistė BalčiūnaitienėMagdalena FrańskaAiti VizziniMagdalena Frej-MądrzakAitor MaestroMagdalena GantnerAiva PlotnieceMagdalena GozdowskaAivars VembrisMagdalena GrajzerAivars ZhurinshMagdalena HurkaczAivett BilbaoMagdalena IwanAixiang DingMagdalena IzdebskaAjaikumar KunnumakkaraMagdalena Jastrzębska-WięsekAjay D. PingaleMagdalena KapłanAjay KumarMagdalena KolasaAjay Kumar ChinnamMagdalena KotańskaAjay NayakMagdaléna KrulováAjaya Kumar SinghMagdalena KrystyjanAjaz AhmedMagdalena LaskowskaAjinkya KawaleMagdalena LigorAjit K. ParhiMagdalena LisAjit RoyMagdalena MaciuszekAjmal KhanMagdalena Makarska-BialokozAjmal Koya PulikkalMagdalena MałeckaAjmer Singh GrewalMagdalena MalikAjmir KhanMagdalena Markowicz-PiaseckaAkalesh Kumar VermaMagdalena MatczukAkbar IdhayadhullaMagdalena Mielczarek-PutaAkbar KaramiMagdalena NarajczykAkhilesh RaiMagdalena OćwiejaAkhlaq Ahmad BhattiMagdalena PaczkowskaAkhtar NadhmanMagdalena RadovicAkifumi SugiyamaMagdalena RogulskaAkihide RyoMagdalena RutkowskaAkihito YokosukaMagdalena SałdykaAkil AhmadMagdalena Skunik-NuckowskaAkilavalli NarasimhanMagdalena SzumeraAkinori KideraMagdalena WójciakAkira InoueMagdalena Wójciak-KosiorAkira KatsuyamaMagdalena WronaAkira KitamuraMagdalena Zielińska-DawidziakAkira MonkawaMagdalena ŻukAkira WanikawaMagdalena ZybertAkiyoshi SawabeMagdalene J. SeilerÁkos KukiMagdaleno R. Vasquez, Jr.Akram HijaziMagdalini HatzikamariAkram Hosseinian SerajehlooMaged E. MohamedAlaa A. M. Abdel-AzizMaged Elsayed Ahmed MohammedAlaa El Din MahmoudMaged M. A. FoudaAlaa KamelMagna GabrieleAlain Favre-RéguillonMagnus SchmidtAlain SarauxMagnus Wolf-WatzAlakananda DasMahadevamurthy MuraliAlam Md BadrulMaharshi BhaswantAlam ZebMahaveer KurkuriAlami AnouarMahavir SinghAlan B. BrownMahboob AlamAlana Gabrieli De SouzaMahbubul MatinAlana ThackrayMahdi MirzaeeAlbena StoyanovaMahdi Niknam ShahrakAlbert FlotatsMahdie AzariAlberto A. Escobar-PuentesMahendiran DharmasivamAlberto BaldelliMahendra D. ChrodiaAlberto CozMahendra SharmaAlberto Da Nova AraújoMahendra SinghAlberto De OliveiraMahendran SekarAlberto FalchettiMaher A. KamelAlberto GallegosMahesh AttimaradAlberto García-PeñasMahesh KumarAlberto Jorge Oliveira LopesMahesh ParitAlberto MarraMahesh PattabiramanAlberto MartínezMaheshkumar Prakash PatilAlberto MezzettiMahi M. MohiuddinAlberto MinassiMahjoub JabliAlberto VitaliMahmood AhmedAlbino CarrizzoMahmood Alizadeh SaniAlcilene Rodrigues MonteiroMahmood BaraniAlciviadis-Constantinos CefalasMahmood TajbakshAldo NicosiaMahmoud A. Al-SamanAldo PezzutoMahmoud Abo-AshourAldo TavaMahmoud Amouzadeh TabriziAldona BeganskienėMahmoud BalbaaAlejandra García GarcíaMahmoud BendaryAlejandra TorresMahmoud ElfakyAlejandro A. TapiaMahmoud El-MaghrabeyAlejandro BaezaMahmoud ElsayedAlejandro BugarinMahmoud El-ShahatAlejandro Islas-JácomeMahmoud IbrahimAlejandro PlascenciaMahmoud KandeelAlejandro UrzuaMahmoud NasrAleksandar KondinskiMahmoud S. M. MohamedAleksandar M. VeselinovicMahmoud SalehAleksandar Ž. KostićMahmoud SayedAleksander BilewiczMahmoud SofyAleksander BurilovMahnaz EstekiAleksander ShtilMahnaz NajafiAleksander V. VasilyevMahomet NjoyaAleksandr A. RubelMahwash MukhtarAleksandr AgafontsevMaialen Espinal-ViguriAleksandr Andreevich BaykovMaicol ManciniAleksandr S. KazachenkoMaija NissinenAleksandra A. JovanovićMaikel Yusat BallesterAleksandra BenkoMaitane AsensioAleksandra BocianMaitree SuttajitAleksandra DeptuchMaja BaretićAleksandra DimitrijevićMaja C. PagnaccoAleksandra GavaricMaja ČolnikAleksandra GrzelakowskaMaja D. NešićAleksandra Janošević LežaićMaja DentAleksandra JózefczykMaja HitlAleksandra KoraćMaja Jazvinšćak JembrekAleksandra KotyniaMaja KatalinićAleksandra KrolickaMaja LeitgebAleksandra MarciniakMaja Matoša KočarAleksandra MaršavelskiMaja MiskulinAleksandra MišanMaja PotokarAleksandra NastasovićMaja RadetićAleksandra NesicMaja WelnaAleksandra OwczarekMajdi HochlafAleksandra Piechota-PolanczykMajid GhashangAleksandra RedzickaMajid HosseinzadehAleksandra RodackaMajid S. JabirAleksandra Sałagacka-KubiakMajid Sharifi RadAleksandra SzcześMakarona EleniAleksandra SzydłowskaMakoto MurakamiAleksandra WdowczykMakoto NishiyamaAleksandra WilczyńskaMakoto NodaAleksei A. TitovMakoto TsunodaAleksei KaledaMakoto YamaguchiAleksei MachulkinMaksim RebezovAleksei V. AlmaevMaksym M. FizerAleksey KuznetsovMaksym PogorielovAlena StuparMakusu TsutsuiAleš LapanjeMalamataris StavrosAleš ZadražilMalček MichalAlessandra BaldiMalcolm JoyceAlessandra CarrubbaMalek GassoumiAlessandra ColciagoMałgorzata CieślakAlessandra De Souza BarbosaMałgorzata DołowyAlessandra NoriciMałgorzata E. ZakrzewskaAlessandra PalladiniMalgorzata GajewskaAlessandra RicelliMałgorzata GniewoszAlessandra StacchiottiMałgorzata GrabarczykAlessandro BenedettiMałgorzata GrembeckaAlessandro BonardiMałgorzata JarończykAlessandro CasnatiMalgorzata JaworskaAlessandro FassinaMałgorzata JeleńAlessandro GalendaMałgorzata JurakAlessandro GenoniMałgorzata Kabsch-KorbutowiczAlessandro GrinzatoMałgorzata KapralAlessandro MandoliMałgorzata KozyraAlessandro MarianiMalgorzata Krok-BorkowiczAlessandro MarroneMałgorzata KrólAlessandro MaugeriMalgorzata KucinskaAlessandro PaiardiniMałgorzata KujawskaAlessandro PesaresiMałgorzata Małodobra-MazurAlessandro PontiMalgorzata Miazga-KarskaAlessandro ScarsoMałgorzata MironiukAlessandro TonacciMałgorzata MusiałAlessandro Vito TaddeoMałgorzata Muzolf-PanekAlessandro ZappiMalgorzata NowackaAlessia BacchiMałgorzata OchotaAlessia CatalanoMałgorzata OlejnikAlessia CiogliMałgorzata PiecykAlessia DavidMałgorzata RogalińskaAlessia LodiMalgorzata RozanowskaAlessio AlesciMałgorzata StarowiczAlessio DessìMalgorzata SzafarzAlessio PortaMałgorzata SzczukoAlessio ReggioMałgorzata SztankeAlessio ZulianiMałgorzata Szultka-MłyńskaAlethia Muñiz-RamirezMałgorzata SzymiczekAlex Bruno Lobato RodriguesMałgorzata TabaszewskaAlex De OliveiraMałgorzata Tatarczak-MichalewskaAlex Gutterres TarantoMałgorzata WasilewskaAlex KhomichMałgorzata WojtkowskaAlex OkaruMałgorzata WroniakAlex T. De Théodore AtchadéMalgorzata Zakłos-SzydaAlex V. ZakharovMałgorzata Zienkiewicz-StrzałkaAlexander A. KorlyukovMaliha AsmaAlexander A. ShtilMalik MaazaAlexander A. SpasovMalina DagmaraAlexander AlijahMalinee SriariyanunAlexander B. VerchovskyMallesh KurakulaAlexander BaykovMallique QaderAlexander BediloMamata SinghviAlexander Benedikt LeichtleMamdouh SamyAlexander BunevMamoru TanakaAlexander DeutschMana MukherjeeAlexander FominManabu KuroboshiAlexander GagarinManar ShoshaniAlexander I. AlexandrovManas KinraAlexander I. KuleffManasés González-CortazarAlexander I. MalovMandana BimakrAlexander J. KastaniotisMandras NarcisaAlexander K. GoroncyManeka MalalgodaAlexander KonevManesh NautiyalAlexander KornienkoMange Ram YadavAlexander KyrychenkoMani DuraiAlexander LarinManić NebojšaAlexander LomzovManickam MinakshiAlexander M. PanichManinder SinghAlexander M. ZakharenkoManish KothakondaAlexander MarinManish KumarAlexander NaumkinManisha NigamAlexander NemukhinManju Rawat SinghAlexander O. ChizhovManjusha LekshmiAlexander OsmolovskiyMan-Kin WongAlexander RufManny MathuthuAlexander S. AntonovManny MinakshiAlexander S. LukatkinManoch KongchumAlexander S. NovikovManohar BhandariAlexander SamokhvalovManohar Prasad BhandariAlexander TerentyevManoj GargAlexander TsouknidasManoj ThapaAlexander V. AksenovManoj TripathiAlexander V. Artem’evManoko S. Maubane-NkadimengAlexander V. OleskinManol Hristov OgnyanovAlexander V. PolezhaevManoli M. ZubiturAlexander V. SkripovManolis FousterisAlexander V. StepakovManolis StratakisAlexander V. VeselovskyMansoor AlshehriAlexander VanetsevMansour HaddadAlexander VtyurinMansour SobehAlexander WittemannMansoureh TatariAlexander ZhbanovMansureh GhavamAlexander ZhgunManuel B. AguilarAlexandr KazachenkoManuel Córdoba-DiazAlexandr PiskunovManuel Gutierrez-AguilarAlexandr ProsekovManuel López-LópezAlexandra Alexandra GasparManuel MedardeAlexandra B. ShintyapinaManuel MinteguiagaAlexandra Cristina BlagaManuel PerisAlexandra Cristina MunteanuManuel PiñeroAlexandra García DuránManuel Tejada-JiménezAlexandra JitǎreanuManuel ValdebranAlexandra KuriganovaManuel ValeroAlexandra M. A. SilvaManuel VazquezAlexandra NistorManuela CalinAlexandra NunesManuela Colla CarvalheiroAlexandra S. SilchenkoManuela CurcioAlexandra SawayaManuela F. FrascoAlexandra ZakharenkoManuela LabbozzettaAlexandre GiacobboManuela LeriAlexandre LamasManuela OliveiraAlexandre M. P. BotasManuela PellegriniAlexandre MagalhaesManuela Poletto KleinAlexandre N. MagasinskiManuela Reyes-EstebanezAlexandrina NanManuela RolliniAlexandros PapachristoforouManuela StefanelliAlexandros RovasManzoor MirAlexandru EvanghelidisMao MinouraAlexey A. KalininMao PengAlexey AleksandrovMaqsood AhmedAlexey BobrovskyMaqsood MalikAlexey ChubarovMara BanovićAlexey ErmakovMara CarsoteAlexey FedorovMara E. M. BragaAlexey KomolovMara Madalina MihaiAlexey M. BogdanovMara MarongiuAlexey MikhaylovMāra PilmaneAlexey N. BilyachenkoMarat EseevAlexey N. SkvortsovMarat GafurovAlexey NikulinMarc MullerAlexey OrlovMarc PignitterAlexey RedkovMarc RegierAlexey S. KononikhinMarc SnapperAlexey S. KubasovMarc TorrentAlexey S. TreninMarc VisseauxAlexey SafonovMarc-Antoine LauzonAlexey ShavardaMarcel BermúdezAlexey SivokhinMarcel IlieAlexey SukhorukovMarcel KaiserAlexey TarasovMarcel KrzanAlexey V. IvanovMarcel Matei DudaAlexey V. Medved’KoMarcel PârvuAlexey V. SalinMarcel PopaAlexey VasilchenkoMarcela AragonAlexey YashchenokMarcela De MoraesAlexia BarbarossaMarcela-Elisabeta Barbinta-PatrascuAlexsandr ProsekovMarcello CasertanoAlfa SharmaMarcello CeciAlfonsina MilitoMarcello LocatelliAlfonso Alejo-ArmijoMarcello ManfrediAlfonso AnnunziataMarcello NicolettiAlfonso Fernández-GonzálezMarcello TagliaviaAlfonso IadonisiMarcelo A. LimaAlfredo CaturanoMarcelo Andrés MuñozAlfredo Di LeoMarcelo Andrés Umsza-GuezAlfredo Estrada-AnguloMarcelo AvenaAlfredo J. IbáñezMarcelo Barcellos Da RosaAlfredo Saavedra-MolinaMarcelo Bispo De JesusAlfredo TeixeiraMarcelo CatalanAlfredo Téllez-ValenciaMarcelo D. CatarinoAlhumaidi AlabbasMarcelo FernandesAli AltwayMarcelo FerreiraAli AziziMarcelo FranchinAli BahadurMarcelo Henrique GehlenAli DadMarcelo J. NietoAli DehshahriMarcelo P. BemquererAli H. JawadMarcelo Paes BarrosAli IrfanMarcia De FigueiredoAli Kaan KalkanMarcia Regina FranzolinAli MakkyMarcin AndrzejakAli MirzaeiMarcin BalcerzykAli Mohammad AmaniMarcin BudnyAli N. A. KoamMarcin CzerwinskiAli SaeedMarcin CzopAli SakMarcin D. SyczewskiAli ShukurMarcin DębowskiAli ZarrabiMarcin GnybaAliaksandr A. KasachMarcin GronowskiAlibek YdyrysMarcin HeljakAlica PizentMarcin HernesAlice Elena GheneaMarcin KaźmierczakAlice Raphael KarikacheryMarcin KidońAlice VilelaMarcin KosmalskiAlicia BoltMarcin KremieniewskiAlicia RoqueMarcin KruzelAlicja PonderMarcin LemanowiczAlicja TymoszukMarcin LukasiewiczAlicja WzorekMarcin MaździarzAliev Firdavs AbdusamievichMarcin OżarowskiAlin CiobicaMarcin PisarekAlina AdamsMarcin SamiecAlina AlexandrovaMarcin ZielińskiAlina AstefaneiMarcin ZiemniakAlina BączkiewiczMárcio José De Abreu RodriguesAlina BoraMarco A. López-MataAlina ButuMarco A. Loza-MejíaAlina Gabriela RusuMarco Alvarez-PerezAlina Georgescu SerbMarco AnniAlina I. MytarevaMarco Antonio Garza NavarroAlina PaunescuMarco BiagiAlina PlenisMarco BortolusAlina PorfireMarco BrunoAlina Pyka-PająkMarco CattoAlina Violeta UrsuMarco ChiariniAliona Ghendov-MosanuMarco ColellaAlireza HadiMarco ConsumiAlireza Nezamzadeh-EjhiehMarco De AmiciAlireza Reza FarrokhiMarco E. M. PelusoAlireza SarmadianMarco GirolamiAlireza SeidaviMarco GuerriniAlirica SuarezMarco MalferrariAlisa SokolovskayaMarco MartinoAlistair J. LeesMarco MasiAliya NazMarco MazzonnaAlka GuptaMarco MelladoAlla IvanovaMarco MilanesioAlla ZamyatinaMarco NaiaAllen SchoffstallMarco PaolantoniAllison LedouxMarco PiccininiAlma D. Alarcon-RojoMarco PoianaAlma Elizabeth Cruz GuerreroMarco RabuffettiAlmerinda Di VenereMarco RossiAlmira RamanavicieneMarco RoversoAlmudena Marrufo-CurtidoMarco Sanchez-GuerraAlmudena MartiMarco Vittori AntisariAlpesh MaldeMarcos Augusto De Lima NobreAl-Shaimaa F. AhmedMarcos De Oliveira JuniorAltaf MohammedMarcos F. S. TeixeiraAltino Branco ChoupinaMarcos FloresÁlvaro De Jesús Ruíz-BaltazarMarcos José Solache-RíosÁlvaro Fernández OchoaMarcos Leoni Gazarini DutraÁlvaro González-GarcinuñoMarcus ScottiÁlvaro Martínez-CamarenaMarcus Vinicius TresAlvaro MombrúMarek BekirAlvaro VillanuevaMarek BobkoAly El GamalMarek BrzezinskiAlyssa Hidalgo VidalMarek CebratAmaç Fatih TuyunMarek GancarzAmador Menéndez-VelázquezMarek K. BernardAmal A. M. ElgharbawyMarek KalenikAmal GhanimMarek KieliszekAmal KasryMarek KolencikAmal NarayananMarek KowalczukAmalia StefaniuMarek MajdanAmanda PacholakMarek PiątkowskiAmanda-Lee Ezra ManicumMarek PieszkaAmandeep SandhuMarek PietrzakAmandeep SinghMarek SanakAmandine FlouratMarek SikorskyAmani TaamalliMarek StankevicAmany RagabMarek WesołowskiAmar BallabhMarek WiśniewskiAmarajothi DhakshinamoorthyMargarida MaiaAmbreen ShoaibMargarita A. KhimichAmeeduzzafar ZafarMargarita De Lorena Ramos-GarcíaAmeer Fawad ZahoorMargarita E. NeganovaAmel TahaMargarita OrlovaAmelia CataldiMargarita ParraAmelia M. SilvaMargarita Vega-HolmAmelia SalimontiMargaux FresnaisAmer Jassim Al-KhafajiMargherita AmentaAmila AbeynayakaMargherita De RosaAmilra Prasanna De SilvaMargherita MaranesiAmin Abu-HashemMargit DrapalAmin Babaei-GhazviniMaria A. LongoAmin Hamed MashhadzadehMaria A. SulimovaAmin Mousavi KhaneghahMaría África Fernández-PriorAmin Reza RajabzadehMaria Alexandra GuedesAmin ShavandiMaría Ángeles Ferrer AyalaAmin Talebi Bazmin AbadiMaría Ángeles Fontecha-CámaraAminoddin HajiMaria AngelovaAminu MohammedMaria Annunziata M. CapozziAminul IslamMaria Antonietta PanaroAmir AfzalMaria Asun CanteraAmir BashkinMaria AtanassovaAmir H. MosaviMaría Ávila-GálvezAmir M. RamezaniMaria BabakAmir ZadaMaria Barbara RóżańskaAmira F. El-YazbiMaría Belén PascualAmira MoawadMária Budai-SzűcsAmirah GozaliMaria C. GiannakourouAmit AdhikaryMaria C. TeixeiraAmit Baran SharangiMaria CamposAmit JaisiMaria CarafaAmit JaiswalMaria Carla CraveroAmit KhuranaMaría Carmen SeijoAmit KumarMaria Carmen VillaránAmit Kumar HalderMaria Carolina VarelaAmit SharmaMaria Cecilia MansillaAmit V. PandeyMaria ChernyaevaAmitava BanerjeeMaria Chiara CristianoAmith MaroliMaria Chiara De SantisAmiya Kumar PandaMaria Chiara MontiAmjad El-QanniMaria Ciudad-MuleroAmjad HussainMaria Concepcion MartinezAmjad IqbalMaria Consolata MilettaAmjad Islam AqibMaria Cristina AnnesiniAmmar AltemimiMaria Cristina Baracat-PereiraAmmar BaderMaria Cristina De RosaAmmar PatelMaria Cristina PetraliaAmmar TahirMaría D. LópezAmmara SaleemMaria Da Conceição Monteiro André De OliveiraAmmu K. RadhakrishnanMaria Da Graça Costa MiguelAmna ParveenMaría De Cortes Sánchez-MataAmnat JareratMaria De FenzaAmparo RoseroMaría De La Luz Cádiz-GurreaAmr AminMaría De Lourdes García MagañaAmr FoudaMaria De Lurdes CristianoAmr NegmMaria De Lurdes DapkeviciusAmr ShehabeldineMaria De MieriAmrinder SinghMaría Dea-AyuelaAmrita AhluwaliaMaria Del Alamo SanzaAmrita SalviMaria Del Carmen PortilloAmro AmaraMaria Del Cisne Guamán-BalcázarAmro HassaneinMaría Del Mar ContrerasAna A. Feregrino-PérezMaría Del Mar HernándezAna A. Vilas-BoasMaría Del Rosario RoderoAna AlmeidaMaria DoligalskaAna AmićMaría Dolores GuillénAna BelchiorMaría Dolores Llobat BordesAna BeloquiMaria Dolores Rivero-PérezAna BorotaMaria DonniacuoAna BudimirMaria Drussilla BuscemiAna C. FernandesMaria E. FortunăAna C. GomesMaria E. M. AraujoAna Carolina MoreiraMaria Eduarda Machado AraújoAna Carolina Silveira RabeloMaría Elena Estrada-ZúñigaAna ČikošMaria Elena Maldonado CelisAna Čipak GašparovićMaria Eleni SakavitsiAna ĆirićMaria Elisa CrestoniAna Cristina S. FigueiredoMaria Elisabete MachadoAna Cristina SoriaMaria Elisabetta ClementiAna D. Popović-BijelićMaria Enrica Di PietroAna Estela LedesmaMaria Eugénia QueirozAna Filipa PiresMaría Eva González-TrujanoAna GavrilovićMaria ExindariAna Henriques MotaMaria Fátima C. Guedes da SilvaAna I. ArrobaMaria Fátima SilvaAna I. Jiménez-AbizandaMaría Fernanda Montenegro-LandívarAna I. MatesanzMaria Fernandez-HerreraAna Isabel BarbosaMaria Filomena CaeiroAna Isabel Fraguas-SánchezMaría Florencia PignataroAna Isabel Gracia LostaoMaria FrosiniAna JeromelMaria G. ChernyshevaAna Joaquina Perez-BernaMaria G. KhrenovaAna José Jiménez-AraujoMaria Gabriella VillaniAna Jurinjak TusekMaria GavrilescuAna KaluševićMaría GianelliAna KramarMaria GiotiAna Laura De La GarzaMaria Giovana Binder PagnoncelliAna Laura VillasusoMaria Giovanna BuonomennaAna LeahuMaria GoulielmakiAna Leonor Abrahão NencioniMaria Grazia AmmendoliaAna LimaMaria Grazia BottoneAna LourençoMaria Grazia MolaAna Lúcia CardosoMaria Grazia SignorelloAna Lucía Morocho-JácomeMaria Helena FernandesAna M. Carmona-RibeiroMaria Helena SilvaAna M. G. SilvaMaria I. PiloAna M. GeerMariá Inés IslaAna ManhitaMaria IorizziAna Margraida AraújoMaría Isabel Rodríguez LópezAna María BotanaMaria J. Casanueva-MarencoAna María Calderón De La BarcaMaria J. MouraAna María ContentoMaria J. PerryAna María García-BoresMaría Janeth Rodríguez-RoqueAna María García-MoraMaria JastrzębskaAna María GómezMaría Jesús Durán-PeñaAna Maria JosceanuMaría Jesús OrtegaAna Maria MendezMaría Jesús RodrigoAna Maria PiresMaría Jesús Rodríguez-YoldiAna Maria PutzMaria João BarrocaAna Maria Simonet MoralesMaria João CebolaAna Marta De MatosMaria João LimaAna Paula Betencourt Martins AmaroMaria João RamalhoAna Paula Da Costa RibeiroMaria João RamalhosaAna PetronilhoMaria João SousaAna PilipovicMaría José Aliaño GonzálezAna PintoMaría José AurellAna Podolski-RenićMaria Jose De Jesus ValleAna Reis-MachadoMaría José Martínez-EstesoAna ReyMaría José Torralvo FernándezAna Rita Xavier De Jesus GameiroMaria K. RybarczykAna RodrigoMaria KhalikovaAna SilvaMaria KomelkovaAna Sofia Dias MestreMaría L. Flores-LópezAna SousaMaría Laura BoskoAna TeixeiraMaría Laura DántolaAna Teresa Corte-RealMaria LavlinskayaAna TomićMaria LevantiAna Varela CoelhoMaria LeżańskaAna VinhaMaria LopesAna ŽugićMaría Luisa Del Prado AudeloAna-Belen BlazquezMaría Luisa Fernández-de CórdovaAnaïs Pitto-BarryMaría Luisa MarínAnamaria BechirMaria Luísa SerralheiroAna-Maria GalanMaria Luisa TestaAna-Maria GurbanMaría Lujan FerreiraAnamaria HanganuMaría M. ZanardiAnamaria SdrobisMaria Manuela Abreu Da Da SilvaAna-Maria UdreaMaria Manuela GasparAnamarija StankovićMaría Margarita Canales-MartínezAnand AnbarasuMaría Margarita Suárez NavarroAnand Dev TiwariMaría Marhuenda-MuñozAnantha DuddupudiMaria MarinescuAnas AhmadMaria MarudovaAnas AlazzamMaria MerneaAnas ShamsiMaria MohoraAnastasia A. ShvetsovaMaría Moreno-GuzmánAnastasia FrolkovaMaria N. TutukinaAnastasia I. SolomatinaMaria NikolovaAnastasia MitseaMaria Olga VarráAnastasia NazarovaMaria Paola BertuccioAnastasia SpiliopoulouMaria Paola GermanoAnastassia MashentsevaMaria PapatsirouAnatoli PopovMaría Paulina RomeroAnatoliy M. DunaevMaría Perez-JiménezAnatoly A. ShatalovMaria Perla ColombiniAnatoly MitrofanovMaria Pia DonzelloAnatoly N. VereshchaginMaria Pia FerrazAnatoly V. ZherdevMaria PieriAnbalagan SaravananMaría Pilar Vázquez-TatoAnbazhagan SathiyaseelanMaria Pilar VinardellAnca Corina FarcasMaria Pina SerraAnca DinischiotuMaria PoptsovaAnca Maria JuncanMaria R. SerialAnca VasileMaria RaposoAnca ZanfirescuMaria Rayane Correia De OliveiraAnca-Maria ArseniuMaria ReifAnca-Roxana PetroviciMaría Rocío Rodríguez ArcosAn-Chen ChangMaria Rosa GigliobiancoAncuța ChetrariuMaria Rosário MartinsAncuța Elena PrisacaruMaria S MuntyanAnđelija MalenovićMaria Sameiro T. GonçalvesAnders BarlowMaría Shantal Rodríguez-FloresAnders E. SundínMaría Soledad PratsAnderson Oliveira SouzaMaría Sonia FreireAndersone AnnaMaria StrianeseAndi AlijagićMaria SzymonowiczAndong XiaMaria TalmonAndonis HatzidimitriouMaria TassiAndra DinacheMaria Terezinha Serrão PeraçoliAndrás BallaMaria TsimidouAndrás RabMaria TufarielloAndrás SzabóMaria V. TurovskayaAndrás SzarkaMaria Valeria D'AuriaAndraz DovnikMaria Valeria RaimondiAndre AraujoMaria Victoria CardoAndré Da CostaMaría Victoria CastelliAndré DelavaultMária VilkováAndre LopesMaria WierzejewskaAndré MeloMaria ZoumpaniotiAndre O. FalcaoMaria ZychAndré Rolim BabyMariacecilia PasiniAndré SchäferMariadoss Arokia Vijaya AnandAndre SilvaMariaevelina AlfieriAndrea Anna SowislokMariagrazia FortinoAndrea Antonino ScamporrinoMaria-Loredana SoranAndrea AntosovaMarialuigia FantacuzziAndrea ArsiccioMarialuisa MennaAndrea AstolfiMarian BurduceaAndrea AtreiMarian Gabriel BanciuAndrea BaschieriMarian SzebenyiAndrea BenciniMariana Altenhofen Da SilvaAndrea BuneaMariana Buranelo EgeaAndrea CalcaterraMariana BusilaAndrea CaporaleMariana E. GhicaAndrea CarpentieriMariana RochaAndrea CastellanetaMariana SantosAndrea CiccioliMariana Varna-PannerecAndrea CostamagnaMariana-Atena PoianaAndrea DefantMariane Bittencourt FagundesAndrea EhrmannMariangela De RobertisAndrea FinMarianna KapetanouAndrea GaertnerMarianna KocsisAndrea GhiroldiMarianna RaczykAndrea Herrero-CerveraMarianna V. KharlamovaAndrea KissMarianne PrévôtAndrea KliewerMariano Francesco CaratozzoloAndrea Luísa Fernandes AfonsoMariano LagunaAndrea MacchiaMariano Martínez VázquezAndrea MahnMariano StornaiuoloAndrea MartinelliMariarosaria MilosoAndrea MezzettaMaribel NavarroAndrea MottaMaricarmen Iñiguez-MorenoAndrea PaolellaMarie Claude ClochardAndrea PastoreMarie Claude MenetAndrea PautzMarie Hélène David CordonnierAndrea PelusoMarie Laronze-CochardAndrea PerrelliMarie-Aude HiebelAndrea PoloMarie-Christine OnutaAndrea PozziMarie-Hélène AntoineAndrea ProtoMariela PatrignaniAndrea R. NikolićMariella TeránAndrea RagusaMarie-Pierre SantoniAndrea RónaváriMarieta ConstantinAndrea Roxanne J. AnasMarieta L. C. PassosAndrea SalvoMarietta FodorAndrea SpallarossaMariia NesterkinaAndrea SpecialeMarija BanožićAndrea SpitaleriMarija Gavrovic-JankulovicAndrea VanniniMarija HefferAndrea WangorschMarija IvanovAndrea ZattoniMarija JankunecAndreas DamianouMarija JozanovićAndreas EisenreichMarija KovačAndreas HauserMarija LesjakAndreas HerrmannMarija M. BabićAndreas KoschellaMarija MilanovićAndreas KösterMarija RadoicicAndreas PetryMarija TakicAndreas ReiberMarijan BubolaAndreas S. KalogirouMarijana AčanskiAndreas SchnepfMarijana PopovićAndreas StaschMarijana Radić StojkovićAndreas TsakalofMarijana SkorićAndreea RatiuMarijana Zovko KončićAndreea-Iren SerbanMarijo BuzukAndreev YaroslavMarilena CarboneAndrei EgorinMarilena E. LekkaAndrei HonciucMarilena GilcaAndrei I. KhlebnikovMarilena RadoiuAndrei IvanetsMarilene DemasiAndrei LucaMarili Villa Nova RodriguesAndrei MostovshchikovMarilia BarrecaAndrei OkhokhoninMarilia M. HornAndrei PotapovMarilia Oliveria Fonseca GoulartAndrei RotaruMarilisa LeoneAndrei SarbuMarin KrapacAndrei V. KovalevskyMarin MicutzAndreia CorciovaMarin ŞenilăAndreja GoršekMarin TortiAndreja Leboš PavuncMarin UgrinaAndreja R. LeskovacMarina Aleksandrovna DarenskayaAndres Barajas-SolanoMarina DerkhoAndrés González SantanaMarina DonovaAndrés M. DurantiniMarina JovanovićAndrés MorenoMarina KirillovaAndresa BerrettaMarina KostićAndrêssa Silva FernandesMarina KrjuchkovaAndrew BreksaMarina RumyantsevaAndrew Chih Wei HuangMarina S. DukhinovaAndrew HectorMarina S. ZelenskayaAndrew HungMarina SalaAndrew LowellMarina StrakhovskayaAndrew McDonaldMarina T. TomovićAndrew PetitMarina V. DunaevaAndrew R. BurgoyneMarina V. PadkinaAndrew S. HursthouseMarina VillalonAndrew WestwellMarina Yu. StogniyAndrew WillettsMarina ZekićAndrey A. VoshkinMarino ParoliAndrey BogomolovMarino ResendizAndrey BuglakMarinônio Lopes CornélioAndrey BukhtiyarovMario AbateAndrey ChibiryaevMario Albert Ruiz-LópezAndrey ElchaninovMario Ferreira Conceição SantosAndrey GatinMario GutiérrezAndrey GeneralovMario InclánAndrey IvanovMario MikulaAndrey KhalimonMario Nikola MužekAndrey KistanovMario PrejanòAndrey KuskovMario RetaAndrey MarkovMário RosadoAndrey MereshchenkoMario SalazarAndrey NaumovMário SilvaAndrey O. DoroshenkoMario SimirgiotisAndrey P. ZhdanovMario VarcamontiAndrey Poddel'skyMario Vincenzo RussoAndrey R. SuprunMario WaserAndrey StavrianidiMario ZorattiAndrey V. KustovMariola A. DietrichAndrey V. SybachinMariola Koszytkowska-StawińskaAndrey YaroslavtsevMariola KozłowskaAndria Agusta AgustaMariola NapiórkowskaAndrii KashubaMariola Zielińska-BłajetAndrii MaryninMarion EhrichAndris JankevicsMarion Zammit MangionAndriy KovalenkoMarios G. KostakisAndriy KytsyaMarios G. KrokidisAndriy SynytsyaMarios SpanakisAndrzej AmbroziakMaris CinelliAndrzej BąkMaris LaubertsAndrzej BilMaris TurksAndrzej CendrowskiMarisa NicolaiAndrzej MiniewiczMarisol Juan-BorrásAndrzej OkuniewskiMarisol VillalvaAndrzej PółtorakMaritza Omaña-MolinaAndrzej PuszkaMarius Emil RusuAndrzej RegiecMarius GhejuAndrzej StepulakMarius StefanAndrzej WasikMariusz FleszarAndrzej ZiębaMariusz HartmanAneta Agnieszka BartosikMariusz JasińskiAneta JezierskaMariusz KepczynskiAneta KopećMariusz MojzychAneta NowakiewiczMariusz SikoraAneta Otocka-KmiecikMariusz Tomasz DziadasAneta PogorzelskaMariusz WitczakAneta StachowiczMariya V. EdelevaAneta SzymańskaMariya VorobyevaÁngel AmestyMariyana AtanasovaÁngel Berenguer-MurciaMarjan VračkoAngel Carbonell-BarachinaMark AgostinoAngel GalabovMark B. BushuevAngel GuerreroMark B. FramptonAngel Gustavo Salas-LaisMark BradleyAngel L. PeyMark ClemensAngel Martin-PendasMark E. DumontÁngel Ramos-OrganilloMark Edmund SmithÂngela BritoMark J. CalcottAngela CapocefaloMark J. RobertsonAngela CorvinoMark N. KobrakAngela De BonisMark P. HeitzAngela De SimoneMark WeaverAngela Di SommaMark WelkerAngela IvaskMark WunderlichAngela Maria MartinsMarkel VinogradovAngela MarottaMarko AnderluhAngela PattiMarko GerićAngela Privat-MaldonadoMarko JukičAngela SantilioMarko LehtonenAngela SardoMarko MilerÁngela Sastre-SantosMarko R. CincovićAngela TartagliaMarko S. MarkovÁngeles DomínguezMarko TrajkovskiÁngeles Peña-GallegoMarkus BergerAngelica Plata-RuedaMarkus BurschAngelika Baranowska-ŁA̧czkowskaMarkus MandlAngelika KmitaMarkus MetsäläAngelika Tkaczyk-WlizłoMarkus MüllnerAngeliki GiannoulisMarkus NaglAngelita M. BarcellosMarkus W. RibbeAngelo AlbiniMarkus WolfienAngelo BañaresMarlena Gąsior-GłogowskaAngelo Del MondoMarlena Zielinska-GorskaAngelo GismondiMarlise Inez KleinÂngelo LuísMarlon Henrique E. Silva CardosoAngelos SikalidisMarole M. MalulekaAngga HermawanMarquez-Rocha Facundo-JoaquinAngie Herrera RamírezMars G. SharapovAngiola ForleoMarsen Garcia Pinto CoelhoAnh Duc NguyenMarta AdamiakAnhe WangMarta Baranowska-KuczkoAnibal De Freitas Santos JuniorMarta BautistaAnibal DisalvoMarta Castineira ReisAnicuta StoicaMarta CiecierskaAniekan Magnus UkpongMarta Coimbra MarquesAnife AhmedovaMarta Denel-BobrowskaAniko BorbasMarta Ferreiro-GonzálezAnikó PósaMarta FijałkowskaAnil Kumar KalvalaMarta GalloAnil Kumar SirohaMarta Hałas-WiśniewskaAnil Kumar YedluriMarta KaszowskaAnil Reddy MarriMarta KepinskaAnil TharappelMarta Klimek-SzczykutowiczAnima GhosalMarta KopaczyńskaAnimesh SamantaMarta KopańskaAnindityo PatmonoajiMarta LaranjoAniruddha DeyMarta LemieszekAniruddha MondalMarta Martínez-GuitiánAnirudh KumarMarta Mazurkiewicz-PawlickaAnirudh SrivastavaMarta Medina O’DonnellAnish KhanMarta NapieralaAnisoara CimpeanMarta NevesAnissa ChouikhaMarta OleszekAnisur RahmanMarta Pastor-BeldaAnita BosakMarta ReboiroAnita ChaudharyMarta SochockaAnita LiškaMarta Sofía ValeroAnita ŠalićMarta StrugaAnita SosnowskaMarta Tsirigotis-ManieckaAnja KolaričMarta ZagrebelskyAnjan BediMartha Cecilia Rosales-HernándezAnjana K. ValaMartha Elena LondoñoAnka Trajkovska PetkoskaMartha Martínez-GordilloAnkana KakotiMartha Rocío Moreno-JimenezAnkit RochaniMartha WellsAnkita PunethaMartial CaillaudAnkita SinhaMartijn De KoningAnkur VaidyaMartin A. MasuelliAnmol KumarMartín AlujaAnna A. EfimovaMartin BaumgartenAnna A. OkunkovaMartin BojinovAnna BaldisserottoMartin BrehmAnna BelcarzMartin BrezaAnna BeniniMartin G. O'TooleAnna BielenicaMartín González-AndradeAnna BieniekMartin GottelandAnna BiernasiukMartín J. RiveiraAnna BirkováMartin KotoraAnna CalarcoMartin KrssakAnna CarforaMartin LeníčekAnna Cleta CroceMartin PéčAnna CzubaszekMartin R. JohnstonAnna DalinovaMartin RabeAnna De FalcoMartin SztachoAnna DolegaMartin VlkAnna Domínguez-VidalMartin VogtAnna DrabczykMartin WittAnna Duda-MadejMartina BancirovaAnna ElkinaMartina BortolamiAnna FilipekMartina HrastAnna FornellMartina JakovljevicAnna GajdaMartina KieningerAnna GalickaMartina SmolićAnna GliszczynskaMartina Srajer GajdosikAnna GrendaMartina VargaAnna GrygierMartina VermathenAnna GubalMartis GeorgianaAnna GumieniczekMartyn E. PembleAnna Helena MazurekMartyna ElasAnna JaneckaMartyna Ewa Maszota-ZieleniakAnna JelińskaMartyna WieczorekAnna JurowskaMaruf AhmedAnna Kaczmarek-KędzieraMaruti Jayram DhanavadeAnna Karin BelfrageMaruyama NaokiAnna Kasielska-TrojanMarvin A. Soriano-UrsúaAnna KazachenkoMarwa A. A. FayedAnna Kiełtyka-DadasiewiczMarwa ElkadyAnna KilanowiczMarwa IssaAnna KleczkaMary BatistaAnna Kostecka-GugalaMary Cano-SarabiaAnna KubíčkováMaryna StasevychAnna KurenkovaMaryna V. StasevychAnna KwiecieńMarysol AlvearAnna L. LussMarzanna KurzawaAnna LesniakMarzena Włodarczyk-StasiakAnna Lucia TorneselloMarzia Bruna GariboldiAnna M. BrudziszMarzia VasarriAnna M. Kurek-GóreckaMarzio RancanAnna MarabottiMaša BuljacAnna Maria AlmericoMaša Islamčević RazboršekAnna Maria CocliteMasabumi MinamiAnna MarońMasafumi TanakaAnna Marzec-GrządzielMasafumi UnnoAnna MaślankaMasahiko HadaAnna Merecz-SadowskaMasahiko TsuchiyaAnna MichalewiczMasahiko YamaguchiAnna MinariniMasahiro NojiAnna MkrtchyanMasahiro WakaoAnna N. BerlinaMasahiro YamasakiAnna NebishMasahito YoshidaAnna NowakMasakazu MorimotoAnna OniszczukMasaki FujitaAnna Palko-ŁabuzMasami IshimashiAnna PanekMasamitsu MaekawaAnna PannaccioneMasanobu MoritaAnna PawełczykMasanori KatakuraAnna PęksaMasanori KoshimizuAnna PetruczynikMasanori MurakamiAnna PiccirilloMasao TakayanagiAnna PilutinMasaru HashimotoAnna Pla-QuintanaMasashi KawamiAnna Przybylska-BalcerekMasataka SunagawaAnna Renata DembskaMasatake SugitaAnna Rita BiliaMasatoshi WatanabeAnna Rita MigliaccioMasayuki HashimotoAnna RonowskaMasayuki NashimotoAnna RuszczyńskaMasayuki SatakeAnna Rybarczyk-PlonskaMasayuki TakahashiAnna RząsaMasfer AlkahtaniAnna Rzepecka-StojkoMashiar RahmanAnna SadowskaMashooq Ahmad BhatAnna SanninoMasood KhanAnna SansoneMasooma IbrahimAnna ShtroMasoud MadadelahiAnna SiemiątkowskaMasoud Nazarian-SamaniAnna SpagnolettaMasoud Salavati-NiasariAnna StępniowskaMasoumeh GhalkhaniAnna StójMasrina Binti Mohd NadzirAnna StojakowskaMassimiliano AmmirabileAnna SygudaMassimiliano BianchiAnna SzymczykMassimiliano CordaroAnna TrubetskayaMassimiliano GaetaAnna Trusek-HolowniaMassimiliano MeliAnna TuśnioMassimiliano PerducaAnna ValentinoMassimo Delle PianeAnna VilàMassimo La DedaAnna W. SobanskaMassimo LazzariAnna Wajs-BonikowskaMassimo MariottiAnna Wiktorowska-OwczarekMassimo NabissiAnna Winiarska-MieczanMassimo SerraAnna Wójcik-AugustynMassimo StefaniAnna WołowiczMassimo ZeraniAnna ZdziennickaMassinissa YahiaAnna Zielińska-JurekMassoud KaykhaiiAnnagiulia Di TranaMastoura Mohamed EdreesAnna-Irini KoukkouMasudur RahmanAnnalisa BarbieriMat Amin AmizaAnnalisa ChiavaroliMatan MusselAnnalisa ContursiMateja GermAnnalisa GuaragnaMateo AlajarínAnnalisa PaoloneMateus Henrique PetrarcaAnnaluisa MaricondaMateusz BrelaAnnamária PallagMateusz DaśkoAnnarita StringaroMateusz GierszewskiAnne Harduin-LepersMateusz KowalikAnne M. WilsonMateusz MołońAnne VessieresMateusz MrukiewiczAnnelaure DamontMateusz PizAnne-Laure RolletMateusz WaliczekAnne-Marie ReißnerMateusz WoźniakAnnette LisMatheus MarconAnnette Suleika Ortiz MirandaMathieu Di MiceliAnoop ArunagiriMathieu PucheaultAnoop AyyappanMathilde Body-MalapelAnoop KumarMathilde BouchéAnoop RawatMati KarelsonAnouar AlamiMatiss OzolsAnroop NairMatjaž KristlAnselm HornMatt MarlowAntal RockenbauerMattanjah De VriesAntal UdvardyMatteo A. RussoAnte MiličevićMatteo BonomoAnte PrkićMatteo BrigantiAnter El-AzabMatteo GuidottiAnthi KarnaouriMatteo MartiAnthia MatsakidouMatteo MoriAnthimia BatrinouMatteo RiccòAnthony J. BaucumMatteo SavastanoAnthony J. BerdisMatteo SensiAnthony Neal WatkinsMatthew BakerAnthony O'DonoghueMatthew BeardAnthonymuthu SelvarajMatthew BrichacekAnti RohumaaMatthew DonaduAntía González PereiraMatthew GrovesAntigoni G. MargellouMatthew McMillinAntimo CutoneMatthew O’RourkeAntionette L. WilliamsMatthew Y. LuiAntje HuefnerMatthias BallauffAntoanela PatrasMatthias EckhardtAntoine LeydierMatthias NeesAnton A. AnisimovMattia AstiAnton E. ShikovMattia BartoliAnton I. RosenbaumMatylda ResztakAnton LarenkovMaura MalinskaAnton Lvovich MaksimovMaura PavesiAnton ManakhovMaura Téllez-TéllezAnton MostovoyMaurice CollinsAnton S. TkachenkoMaurício Dos Santos PereiraAnton TerasmaaMauricio Homem-de-MelloAntonela Ninčević GrassinoMauricio L. CafieroAntonella ArestaMauricio LlaverAntonella CaniniMauricio Moncada-BasualtoAntonella Caterina BocciaMauricio MoraisAntonella CurulliMauricio MorelAntonella LeggioMauricio Rodriguez-dorantesAntonella MessoreMauricio SarabiaAntonella MinutoloMaurício Temotheo TavaresAntonella RosiMaurizio BattinoAntonella SmeriglioMaurizio BenagliaAntonella VirgilioMaurizio MandalàAntonella ZannettiMaurizio MattarelliAntonelli MichelaMaurizio MulasAntoni SzumnyMauro AdamoAntoni WiedlochaMauro AngelettiAntonia Ávila-FloresMauro BancheroAntonia Di MolaMauro BolognaAntônia Eliene DuarteMauro Carlos Costa RibeiroAntonia MancusoMauro Cesar GeraldesAntonieta RuizMauro ColucciaAntonina ArgoMauro FormicaAntonina I. KarlinaMauro MagnoniAntonino BiundoMauro Manuel Martinez PachecoAntonino LauriaMauro MartínezAntonino TestaMauro SattaAntonio ApicellaMauro Sergio PavaoAntonio ArtiguesMax K. LeongAntonio BauzáMax MajireckAntonio BelcariMax RyadnovAntonio Bernabé-AntonioMaxim A. DubinnyiAntonio CarrieriMaxim A. GureevAntonio ColucciaMaxim GelinAntonio De Leon RodriguezMaxim PiskunovAntonio Del VecchioMaxim PyatnovAntonio Di BartolomeoMaxim S. KokoulinAntonio Di MartinoMaxim V. MusalovAntonio Diego Molina GarciaMaxime PichonAntonio Diogo Silva VieiraMaxime RobinAntonio Doménech-CarbóMaya M. ZaharievaAntonio Eduardo Miller CrottiMayada Ragab FaragAntonio EvidenteMayra Beatriz Gómez-PatiñoAntonio FacchianoMayumi KomineAntonio FranconettiMayur K. LadumorAntonio FronteraMayya RazgonovaAntonio GaldamezMazen AlmehmadiAntonio Garrido-FernándezMazen KhaledAntónio Gil CastroMazen NazalAntonio GiordanoMd Abdus SubhanAntonio GloriaMd AdnanAntonio Gonzalez-CasadoMd Amirul IslamAntónio H. S. DelgadoMd Atiar RahmanAntonio Henrique MartinsMd Hakimul HaqueAntonio J. Martinez-MartinezMd Jahoor AlamAntonio J. MotaMd Jamal UddinAntonio J. MuñozMd Jawaid AkhtarAntonio J. Pérez-LópezMd Kausar RazaAntónio Jorge ParolaMd MushtaqueAntónio M. PeresMd Nabiul IslamAntonio M. ScarfoneMd Nazmul Hasan ZilaniAntónio MachadoMd ShahinozzamanAntonio MassaMd Tabish RehmanAntonio MonopoliMd Toufiqur RahmanAntonio Pulido-BoschMd Zahid AkhterAntonio RandazzoMd. Abdul HannanAntónio RangelMd. Abu HanifAntonio Romo-MancillasMd. Akter HosenAntonio Rosales MartínezMd. AsaduzzamanAntonio SalomoneMd. Ashraful Islam MollaAntonio SansonettiMd. Imtaiyaz HassanAntonio TraniMd. Maidul IslamAntonio V. Herrera-HerreraMd. Moshfekus Saleh-E-InAntonio Valle GallardoMd. Nuralam HossainAntonio Valverde-GonzálezMd. Salatul Islam MozumderAntonio Vidal-PuigMd. SharkerAntonio ZandonaMebin SamuelAntonio ZuorroMedhi RiddhimanAntonio-José TrujilloMeena GhoshAntonios KolocourisMeenakshi SharmaAntonios M. MakrisMeerambika MishraAntonis GitsasMegat HanafiahAntonius R. B. OlaMegawati ZunitaAntony KamMehdi AyouzAntony R. ThiruppathiMehdi GhalambazAnu KaliaMehdi Hatefi-ArdakaniAnuj KumarMehdi MehraliAnuj RanjanMehdi Mogharabi-ManzariAnuja YadavMehdi Nasr IsfahaniAnup K. PaulMehdi NikooAnúp PandíțhMehdi PordelAnupam Das TalukdarMehdi ShahrakiAnuradha RoyMehdi Shakourian-FardAnurag KumarMehmet L. YolaAnurag MisraMehmet OzcanAnurag RoyMehmet SenelAnusorn SeubsaiMehran AlaviAnwar RayanMehran KarimzadehAnwar UsmanMehrdad RostamiAnyanee KamkaewMehrez E. El-NagarAnyang WangMehtap SahinerAnzar KhanMei Er ChenAnže ŽupaničMei XuApinun KanpiengjaiMei-Chin LuApip AmrullahMei-Chin MongApostolia TsiasiotiMeiji Soe AungApostolos ThomareisMeijia LiApurba Krishna BhattacharjeeMeijuan ZouAraa Mebdir HoliMeiling FengArafa MusaMeixue DongArancha Llama-PalaciosMeiya LiuAránzazu Zarzuelo CastañedaMejdi SnoussiArash MoeiniMekala VenkatachalamArash NikoubashmanMelanie CoombsArash SalahinejadMelgardt De VilliersArashdeep SinghMelih Özgür ÇelikArasu GanesanMelinda FogarasiAravind KajjamMelinda Haydee KovacsAravinda PaiMelinda KrebszAravindan JayaramanMelinda SzekelyArbi GuetatMelisa Del BarrioArchimede RotondoMelissa D. CantleyArdala KatzfussMelissa Michelle CadelisAree ChoodumMelkie Getnet TadesseAref Abbasi MoudMeltem YanilmazArellano María EvaristaMelvin E. KlegermanArghya GhoshMelvin LeowAri HashimotoMelzig MatthiasAri KoivistoMeng CuiAri LehtonenMeng GaoAri Satia NugrahaMenglong XuArian VelayatiMengmeng LiAriane RozzaMengxia LiuArianna MarengoMengxing LiArie WardhonoMengyuan ChenAriel Colaço De OliveiraMengze LyuAriel FontanaMensah-Darkwa KwadwoAriel Geer WallaceMercedes FernandezArie-Lev GruzmanMercedes Salazar HernándezArif A. BabaevMeriel G. JonesArif KhanMershen GovenderArif MermerMery MalandrinoAriful HaqueMeseret AmdeArijit A. AdhikariMesut YurukcuArindam IndraMetha WanapatArindam PramanikMetin GuldasArindam SinharoyMeysam SoleimaniAris GiannakasMeysam VadiatiArjun H. BanskotaMi ChenArkadij SobolevMi Jeong KimArkadiusz JarotaMia BrkljačaArkadiusz MatwijczukMia EricsonArkadiusz OrchelMian NavedArkadiusz SzpicerMiano TeodoroArkajyoti PaulMiao ZhangArkajyoti SenguptaMiao-Hsia LinArkamitra KarMiaorong YuArlene Gonçalves CorrêaMicael De Andrade LimaArmando Alexei RodríguezMichael A. FirerArmando CaseiroMichael A. LeaArmando J. D. SilvestreMichael A. ShestopalovArmando ZarrelliMichael AppellArmandodoriano BiancoMichael AssfalgArmida Sánchez-EscalanteMichael BertocchiArmin TarrahMichael BowmanArnab GhoshMichael BühlArnab RitMichael CampbellArnaldo César PereiraMichael CromptonArnaud BondonMichael DzakovichArnaud Kamdem TamoMichael EskinArnaud PallottaMichael G. WellerArnd BaumannMichael GhijsArno PfitznerMichael J. McGlincheyArnold GrünwellerMichael Jacob IoelovichArnoldo Aquino-GálvezMichael JanickeArokia Mariyadoss VijayaanandMichael John PlaterAroldo José Romero-AnayaMichael MarnerArpád DobolyiMichael O'ConnorArpad Mihai RostasMichael PissasArpita RoyMIchael QianArpron LeesombunMichael R. HamblinArrigo CiceroMichael Rivera MananghayaArrigoni FedericaMichael RothArseniy LobovMichael RustArshad Husain RahmaniMichael SchalliArtem A. SelyutinMichael SevillaArtem AbakumovMichael SimonichArtem BelousovMichael SmirnovArtem BezrukovMichael StevensonArtem ChizhovMichael TellierArtem GushchinMichael TsangArtem KozlovskiyMichael W. BeckArtem MarikutsaMichael WarkArtem MitrofanovMichael WoeltjeArtem OsypenkoMichaela Schulz-SiegmundArtem RogachevMichail SyrpasArtem SurovMichał BijakArtem Yu ManyakhinMichał Błazej PonczekArthitaya Kawee-aiMichal CeglowskiArthur D. TinocoMichal CzerwińskiArthur G. RobertsMichał GleńskArthur R. TulyabaevMichal JaniakArtur BeberokMichal JurášekArtur ChrobakMichal LetekArtur KasprzakMichał M. WójcikArtur KhannanovMichal MachovskýArtur MuchaMichał MajewskiArtur NowakMichal NiemczakArtur SewerynMichał NowakowskiArtur SzwengielMichał OtrębaArtur ZalevskyMichał PiegzaArturo Cadena RamírezMichal PyzikArturo Edgar Zenteno-GalindoMichał ŚwiecaArturo Navarro OcañaMichał TomczykArtyom PlyushchMichal ZaliberaArulraj RamalingamMichal ZemanArun JayaseelanMichalis K. ArfanisArun KumarMichel BardetArun Kumar MannaMichel BaronArun Kumar SangaiahMichel F. SannerArun PatilMichel GoldmannAruna DhathathreyanMichel HananiaArunaksharan NarayanankuttyMichel MonsArunas JagminasMichel PfefferArunkumar PalaniappanMichel RavelonandroArvind GuptaMichel RouleuxArvind NegiMichela BuccioniArya UthamanMichele ArestaAsad Ullah KhanMichele CrudoAsako YamayoshiMichele Di FoggiaAsgar A. AliMichele MaffiaAsgar KayanMichele ManfraAsha ThomasMichele MariAshaimaa MoussaMichele NavarraAshfaq AhmadMichele R. ChierottiAshish KabraMichele RetiniAshish N. AphaleMichele RicuperoAshkan BighamMichelle Ji Yeon YooAshok ChhetryMichelle KuttelAshok K. ShakyaMichelle SaoiAshok KumarMichiaki MatsumotoAshok Kumar JangidMichio IwaokaAshok Kumar TiwariMicholas Dean SmithAshraf AliMickaël LafondAshraf ElbendaryMieczyslaw MakoszaAshraf El-BindaryMieczyslaw PietrzykAshraf KhalifaMieke BuntinxAshraf N. AbdallaMieszko WieckiewiczAshton Keith CowanMiguel A. OrtegaAshutosh BahugunaMiguel A. PelliteroAshutosh TripathiMiguel A. PrietoAshwani KumarMiguel A. Puertas-MejíaAshwani MittalMiguel Angel Garcia-AlvaradoAsim JilaniMiguel Ángel González-CurbeloAsiya MustafinaMiguel Ángel Hurtado OlivaAsma Ismail MahmodMiguel Ángel Ruíz CabreraAsmaa M. FahimMiguel Aythami Ayuso SebastianAsmita BanstolaMiguel CardosoAsta JudzentieneMiguel G. Ramírez-ElíasAsta ZubrieneMiguel Ponce-VargasAsta ŽukauskaitėMiguel Rebollo-HernanzAstrid BuicaMiguel SantosAstrid WeißMiha LukšičAstrida VelenaMihaela BacalumAswir Abd RashedMihaela BadeaAtaul IslamMihaela Cristina BaicanAtcharaporn OntawongMihaela DeaconuAtefeh ZarepourMihaela NiculaeAthanasios K. AnagnostopoulosMihaela PerteaAthanasios KatsourasMihaela SaracilaAthanasios KimbarisMihaela TrifAthanasios PapakyriakouMihai BrebuAthanasios PappasMihai NitulescuAthanasios ZarkadoulasMihai TodicăAthanassios K. GiatropoulosMihai Valentin PredoiAtheer ZgairMihail Lucian BirsaAtheesha SinghMihail Lucian PascuAthinarayanan SundaresanMihail MondeshkiAthri D. RathnayakeMihajlo EtinskiAtiah H. AlmalkiMihalis FakisAtilla KormosMihalj PošaAtiyeh MahdaviMihaly MezeiAtsushi IshiharaMihály T. CserepesAtsushi KobayashiMihkel KaljurandAtsushi KuboMijat BožovićAtsushi MuramatsuMika PetterssonAtsushi SekiMikael JensenAtsushi ShimojimaMikako FujitaAtsushi ShishidoMikalai M. KrukAtsushi UchidaMike DrewAtsuya MuranakaMikhail A. Il’yushinAttila BecskeiMikhail BeklemishevAttila BendeMikhail BelonenkoAttila BényeiMikhail DunaevskiyAttila BôtaMikhail KarushevAttila GergelyMikhail KiskinAttila KissMikhail KrasavinAttila ReményiMikhail M. Vorob'evAtukuri DorababuMikhail N. BerlovAudrey LaventureMikhail Nikolaevich LyulyukinAudun Formo BueneMikhail PetrovAugustin C. MotMikhail ProskurninAugustin M. MǎdǎlanMikhail S. DrenichevAugusto C. ToméMikhail ShepelevAugusto Cannone FalchettoMikhail V. KhvostovAunchalee AussanasuwannakulMikhail Yu MoskalikAura RusuMikiya TanakaAurea Ramírez-JiménezMikko I. KettunenAurel TabacaruMiklós KubinyiAurelia Cristina NechiforMiklós NagyAurelia Magdalena PisoschiMikó ÉvaAurelia VisaMilad HadidiAurelian Cristian BoscorneaMilan Babu PoudelAurelio Luna MaldonadoMIlan BeraAurelio San-MartínMilan ČížAurica P. ChiriacMilan JakubekAurica PrecupasMilan KragovićAurora Santos LopezMilan Kumar LalAurora SilvaMilan MalaníkAurore FraixMilan MladenovicAvi WeissbergMilan S. DimitrijevićAvijit JanaMilan SenćanskiAvila-Sosa RaúlMilan StankovicAvinash MishraMilán SzőriAvinash PremrajMilan UrbanAvneesh GautamMilan ZeljkovicAvtar SinghMilardi DaniloAwadh YadavMilena A. VegaAwal NoorMilena Magalhães AleluiaAxel G. GriesbeckMilena MasulloAxel KleinMilena P. PopovaAxel MagalonMilena PanticAxel MethnerMilena PetkovicAyat F. HashimMilena PetriccioneAydi AbdelkarimMilena RašetaAydin HassaniMilena ŚciskalskaAyelén ToroMilena TzanovaAyesha TahirMilena VukicAyman ElkhateebMili DasAyman H. KamelMilica AćimovićAyman M. MostafaMilica Ljaljevic GrbicAyoub KasratiMilica M. SredojevićAyse Günes-BayirMilica MarkovicAyşegül ErdoğanMilica NicetinAysegul Uygun OksuzMilica PavlicevicAyub KhanMilica PešićAyumi SugiuraMilica VujkovicAyuob AghanejadMiljana PricaAyush RanawadeMilka DoktorovaAzadeh NilghazMilka MaleševićAzaj Thaminum AnsariMilka MilevaAzalia Avila-NavaMilos MilcicAzamat TemerdashevMiloš ProkopijevićAzat GabdulkhakovMilton KanashiroAzeddin El BarnossiMilton RoyAzeem MustafaMilton Yutaka Nishiyama JuniorAzeman Nur HidayahMina LeeAzhagiya Singam Ettayapuram RamaprasadMinaxi SharmaAzhar RasulMindaugas LiaudanskasAziz AmineMindaugas MacernisAziz KarakayaMine OzcelikAzizur RahmanMing FangAzrina AzlanMing LiuAzzurra StefanucciMing WangB. NagarajaganeshMing XuBabu JosephMing YangBaby SabulalMingbao FengBachir Raho GhalemMingbo ZhouBadreddine BabesMing-Chi WeiBadrinath KakdeMinggui LinBagrat A. ShainyanMing-Hsi ChiangBahaa YoussifMinghua ChenBahauddeen AlrfaeiMinghua QiuBahman RashidiMinghua ZhangBahram SalehMing-Jiuan WuBahri BaşaranMing-Ju ChenBaihan LinMingjun CuiBaitang JinMing-Jun TsaiBaki HazerMingkang SunBakier SlawomirMing-Kuem LinBakr F. Abdel-WahabMingli LiBala Krishna PrabhalaMingli WangBalaji BakthavatchalamMingliang YeBalaji NagarajanMing-Sheng TengBalaji SambandamMingtao ChenBalakrishnan KirubasankarMing-Tsz ChenBalamuralikrishnan BalasubramanianMingwei WangBalarabe IsmailMing-Yi ShenBalasankar AthinarayananMingyi WuBalasubramani Subramani ParanthamanMingyou HuBalasubramanian MalaikozhundanMingyu QiaoBalasubramanian ViswanathanMinh Vien LeBalázs LászlóMinh-Dung TruongBalazs VargaMin-Hua ChenBalbina J. PlotkinMin-Hui LiBaldomero EsquivelMin-Kyeong LeeBalenahalli RameshMintu ChandraBalik DzhambazovMintu PorelBalu Alagar Venmathi MaranMinxiang ZengBancha YingngamMinyi TianBang Yeon HwangMio MatsumuraBang-Yuan ChenMiona BelovićBanruo HuangMiquel Torrent-SucarratBanzeer Ahsan AbbasiMiran ŠebeštjenBaomin WangMiranda SertićBaosheng XuMircea NasuiBaoxia DongMirco CastoldiBaoyang LuMireille VonlanthenBapan PramanikMirela F. NicolovBappaditya ChandraMirela GhergheBaranovskaia VasilisaMirela MihailaBarbara AzzimontiMirela SucheaBarbara BellichMirela-Fernanda ZaltariovBarbara BlackwellMirella NardiniBarbara BorczakMíriam Anders ApelBarbara CanonicoMiriam Frida KarlssonBarbara ConwayMiriam Ortega-HerasBarbara De FilippisMiriama SimunkovaBárbara FerreiraMirian PateiroBarbara FuenzalidaMiriding MutailipuBárbara J. HenriquesMirjana B. ČolovićBarbara LeśniewskaMirjana ComorBarbara MalawskaMirjana StojkovićBarbara MarczewskaMirko D. De RossoBarbara MavroidiMirko MaturiBarbara MiroslawMirna Estarrón-EspinosaBarbara MognettiMirna Habuda-StanicBarbara Moniuszko-SzajwajMiroslav BarancikBarbara PanunziMiroslav ČernýBárbara Paranhos CoelhoMiroslav JeškovskýBarbara ParrinoMiroslav JůzlBarbara PawłowskaMiroslav M. NovakovićBarbara PieczyrakMiroslav OndrejovičBarbara SawickaMiroslav ŽivićBárbara Socas-RodríguezMiroslava HlebovaBarbara SzczepankiewiczMiroslava KačániováBarbara SzczęśniakMiroslava NedyalkovaBarbara Teixeira-CostaMirosław AniołBarbara VercelliMirosław DudekBarbara WiewióraMiroslaw GalazkaBarbarella De Matos MacchiMirosław GiurgBaris DemirMiroslaw JablonskiBarkat KhanMirosław MaziejukBarnali MaitiMiroslaw ZagajaBarry LavineMirosława ChwilBartłomiej GórskiMirosława TeleszkoBartłomiej GrygorcewiczMirsada SalihovicBartłomiej IglińskiMirta MilićBartłomiej MazelaMirta R. AlcarazBartłomiej PotaniecMirta RubčićBartłomiej SzulczykMirtha Navarro-HoyosBartłomiej WysockiMisu MoscoviciBartłomiej ZieniukMitja KrizmanBartosz BondziorMitsuaki MoriyamaBartosz HrycakMitsuhiro YoshimatsuBartosz KruszewskiMitsuru TashiroBartosz PiechowiczMittal RochakBartosz TrzaskowskiMladen ParadžikBaruh PolisMladena Lalic-PopovicBasant RoondheMoaamen RefatBasanta SaikiaModestas ŽiliusBasharat Nabi DarModesto Ocen OlanyaBashayer R. Al-MubarakMogens NielsenBaskar VenkidasamyMohamad AzmiBasma NajarMohamad Fakhrul Ridhwan SamsudinBassam El-EswedMohamad Hesam ShahrajabianBassem A. SabryMohamad RafiBassma ElwakilMohamed A. A. OrabiBayirta V. EgorovaMohamed A. El-EsawiBeat C. BornhauserMohamed A. HabilaBeata CristóvãoMohamed A. HassanBeata DedicovaMohamed A. TammamBeata DruzynskaMohamed AbdelgawadBeata Godlewska-ŻyłkiewiczMohamed AbdelwahabBeata JanoszkaMohamed AddiBeata JanowskaMohamed AdelBeata JedrzejewskaMohamed AldlemyBeata KaletaMohamed Al-FatimiBeata KorchowiecMohamed Ali IbrahimBeata Łaźniewska-PiekarczykMohamed AlkafafyBeata Morak-MłodawskaMohamed Al-SayeghBeata NowickaMohamed Aymen ChaouchBeata OlejnickaMohamed Bechir Ben HamidaBeata PolakMohamed BouazizBeata Smieja-KrólMohamed DakirBeata StaniszMohamed El HattabBeata Wielgus-KutrowskaMohamed El RaeyBeate PaulusMohamed El-AassarBeatrice CantoniMohamed El-MeseryBeatrice FerrariMohamed ElnagarBeatrice MacchiMohamed El-NewehyBeatriz Buentello-VolanteMohamed El-RaiyBeatriz E. JaramilloMohamed F. M. IbrahimBeatriz Gil HernándezMohamed Fawzy RamadanBeatriz GinerMohamed G. ShehataBeatriz Hernández-CarlosMohamed Gamal MohamedBeatriz LimaMohamed Hussein Hamdy RobyBeatriz MaciáMohamed Jawed AhsanBeatriz Pérez-FernándezMohamed L. AshourBeatriz Sevilla-MoránMohamed Mehawed AbdellatifBeenu Moza JalaliMohamed MohanyBegoña CerdáMohamed NasserBegoña VerdejoMohamed O. MahmoudBehafarid GhalandariMohamed Osman RadwanBehrooz Alizadeh BehbahaniMohamed RadwanBehrouz ShaabaniMohamed S. HasaninBehrouz ShamsaeIMohamed S. NafieBei GaoMohamed SaidBe-Jen WangMohamed SalaheldeenBéla FiserMohamed SalemBéla PályiMohamed Samir MohyeldinBelén AltavaMohamed ShaabanBelén Callejón LeblicMohamed ShaalanBelen FerrerMohamed Sheikh MohamedBelgin SeverMohamed Z. M. SalemBelkhiri LotfiMohamed ZakriyaBello MartinianoMohamed ZayedBen C. L. ChanMohamed-Bassem AshourBen De Lacy CostelloMohamed-Elamir F. HegazyBen MeinenMohammad AatifBeñat ArtetxeMohammad AbediBence SiposMohammad Akhlaquer RahmanBenedetta MarmiroliMohammad Ali HesarinejadBenedetta SqueoMohammad AshfaqBénédicte F. JordanMohammad Azam AnsariBenedikt M. MortzfeldMohammad B. UddinBenedito C. PrezotoMohammad Bagher AskariBeng Fye LauMohammad Bagher HassanpouraghdamBengü ÖztürkMohammad BaigBenhur GodoiMohammad BasyuniBenita WiatrakMohammad BayanBenjamin NamanMohammad Fahad UllahBenjamin P. FingerhutMohammad Faheem KhanBenjamin R. CaroneMohammad FaizanBenjamin RiouxMohammad FasihiBenjamin SellesMohammad H. SemreenBenjamin TawiahMohammad HojjatiBenneth Ben-AzuMohammad Hossein SayadiBenoit BriardMohammad IkramBenoit ChabotMohammad ImranBenoît GuillotMohammad IrfanBenoît HeinrichMohammad Jamshed SiddiquiBenoît IgneMohammad KashifBenson KariukiMohammad Kazem MohammadiBenyoucef AbdelghaniMohammad Mahboob AlamBeom Jin KimMohammad Manjurul HaqueBeow Keat YapMohammad Mehedi HasanBerenice Fernández-RojasMohammad MofidfarBerenika M. Szczȩśniak-SiȩgaMohammad MohsenzadehBernal-Mercado Ariadna ThalíaMohammad OvesBernard PossidenteMohammad QneibiBernardina ScafuriMohammad Reza Edalatian DovomBernardo A. A. NogueiraMohammad Reza MahmoudiBernardo Castro-DominguezMohammad Reza RouiniBernardo ChavesMohammad RizwanullahBernardo Gonçalves AlmeidaMohammad Ruhul Amin BhuiyanBernardo Lopez-TorresMohammad Safiqul IslamBernat CrosasMohammad Saidur RhamanBernát GáborMohammad Saood ManzarBernd DiehlMohammad ShahidBernd RathkeMohammad SouriBernhard BiersackMohammad Tajul IslamBernhard EllingerMohammad ThaljiBernhard RyffelMohammad Zain KhanBernhard SpinglerMohammadhassan ValadanBernhard WünschMohammadian MehdiBerta Barta HollóMohammadjavad MohammadiBertrand FournierMohammadreza ElahifardBertrand JóźwiakMohammadreza KhorramiBertrand LiagreMohammadreza MohammadabadiBetty Yuen-Kwan LawMohammed A. DahabBhakti BasuMohammed AbbasBharath Kumar VilluriMohammed AkbarBharati NeelamrajuMohammed AlasawshBhaskar BiswasMohammed AsadBhaskar MondalMohammed AurifullahBhasker RadaramMohammed BourhiaBhaswati SenguptaMohammed BuleBhawna UpretyMohammed El-BanaBhekumuzi GumbiMohammed El-MagdBhupendra PrajapatiMohammed El-MowafyBhupinder KapoorMohammed F. AldawsariBhupinder SandhuMohammed F. HamzaBianca FurduiMohammed GagaouaBianca Maria TihăuanMohammed GhazwaniBianca R. AlbuquerqueMohammed H. MohammedBianka SiewertMohammed HassanBibhisan RoyMohammed HawashBidya Dhar SahuMohammed HelalBidyut SahaMohammed IsmaelBifulco AurelioMohammed MajdoubBijan GhalehMohammed Oday EzzatBijender KumarMohammed Qasim ChaudhryBijo MathewMohammed Rafi ShaikBilal Ahamad ParayMohammed RasheedBilal JavedMohammed RhaziBilge Coşkuner FilizMohammed Rizwan KhanBiliana NikolovaMohammed SayedBiljana AbramovićMohan KaruppayilBiljana ArsicMohan Kumar Muthu KaruppanBiljana BalenMohan LalBiljana BufanMohandas MandhakiniBiljana BujanovicMohandoss SonaimuthuBiljana DojnovMohd AdnanBiljana GlišićMohd AsgherBiljana KukavicaMohd Hafiz Mohd ZaidBiljana LoncarMohd Helmy MokhtarBiljana ŠljukićMohd ImranBill DennyMohd Nazam AnsariBilqees BanoMohd Nazri IsmailBimal GhimireMohd Nurazzi NorizanBimal JanaMohd RafatullahBimal KoiralaMohd RaufBimal Kumar GhimireMohd Rehan AjmalBimlesh KumarMohd SaeedBin LuoMohd UbaidullahBin YangMohd Wajid Ali KhanBin Yu (China)Mohd Zaheen HassanBin Yu (Hong Kong)Mohd Zamir PakhuruddinBin ZhangMohd Zuwairi SaimanBinbin LuoMohini GuleriaBing WangMohit ChawlaBing WeiMohnad AbdallaBing YangMohsen ElsharkawyBingchen WangMohsen HesamiBing-Lan LiuMohsen MaghrebiBingpu ZhouMohsen PadervandBingsheng LiMohsin KhurshidBingxin ZhaoMohtadin HashemiBingyou YangMoira AmbrosiBin-Nan WuMoisés Tolentino B. Da SilvaBinoy MaitiMojca Čakić SemenčićBinu TimsinaMojtaba MansourianfarBinwei ZhangMominur RahmanBipeen DahalMona El-AasrBiplab BanerjeeMona El-NeketiBiplab K. MaitiMona I. ShaabanBirgit HetzerMona M. FreidinBirgit LohbergerMónia A. R. MartinsBirgit M. PruessMónica A. Ramírez-CabreraBishnubrata PatraMónica BenitoBishoy Y. A. El-AaragMonica BucciantiniBishweshwar PantMonica ButnariuBissera PilichevaMónica CarreraBistra GalunskaMonica CaselliBistra StamboliyskaMonica De PalmaBiswadeep DasMônica Feijó NaccacheBiswajit BhattacharyaMónica Graciela ParisiBiswajit BhattacharyyaMonica GulatiBiswajit Gopal RoyMónica María Umaña ZamoraBiswajit PandaMonica MironescuBiswajita PradhanMonica NardiBiswanath DindaMonica Rosa LoizzoBiswanath MahantyMónica SantosBiswarup JashMonica TrifBjörn HambergerMonika A. OlszewskaBjörn ThieleMonika BarbarićBjörn VoßMonika BeszterdaBjørnar HasselMonika BorkowskaBjörn-Philipp DiercksMonika CzerwińskaBlair TuttleMonika DąbrowskaBlanca Estela García PérezMonika FabiańskaBlanca F. Iglesias-FigueroaMonika FurkoBlanca Lorenzo VeigaMonika GargBlanca TorresMonika I. HollenhorstBlanco-Canosa Juan BautistaMonika JachBlas Lotina-HennsenMonika Kadela-TomanekBlaž StresMonika KaraśBłażej GierczykMonika KassayováBłażej KudłakMonika KijewskaBo GaoMonika KniotekBo JiangMonika KosmalaBo LiMonika KovačevićBo OuYangMonika Le$\ddot{s}$kiewiczBo PangMonika Marcinkowska-LesiakBo YangMonika MichaelisBo YuanMonika MichalskaBoas PuckerMónika MolnárBoaz G. OliveiraMonika MounBoban AndjelkovicMonika Musiał-KulikBo-Cheng TangMonika NaumowiczBochuan TanMonika PapieżBodo LinzMonika PituchaBogdan BujnickiMonika PrakashBogdan Ionel TambaMonika PrendeckaBogdan L. LesyngMonika SienkiewiczBogdan SevastreMonika SobiechBogdan SmykMonika SpilkaBogdan Stefan VasileMonika Srebro-HooperBoggarapu Praphulla ChandraMonika StaśBoguslaw MichalikMonika SujkaBohan YinMonika TomaniovaBoitumelo Joyce MatsosoMonika Wilamowska-ZawłockaBojan BožićMonika WujecBojan ButinarMonika Żubrowska-SudołBojana DanilovicMonique Mathé-AllainmatBojana IkonićMonjurul HaqBojana ŠarićMonther A. KhanfarBojana VasiljevicMontserrat Mitjans ArnalBojana VidovicMonwadee WonglapsuwanBokanyi LjudmillaMood MohanBokhimi XimMoonsung ChoiBoldeanu Mihail VirgilMorad M. El-HendawyBonga Lewis NgcoboMorana JaganjacBonggi LeeMoreno MarcelliniBon-Jae GuMorgan HawkerBono LučićMorifumi HasegawaBoon Chin TanMorihito TakitaBoonyadist VongsakMorris ArgyleBoonyarach KitiyananMorteza Hasanzadeh KafshgariBoredi Silas ChidiMorteza Sasani GhamsariBorhan AlbissMosa RebamangBoris ErshovMosab KaseemBoris KrichelMoshi GesoBoris MaryasinMoslem AbdipourBoris MinaevMostafa A. HussienBoris PopovicMostafa ElachouriBoris TenchovMostafa ElfawalBoris ZlatopolskiyMostafa GoudaBorislav AngelovMostafa RatebBorislav KovačevićMotasem AlazaizaBorys OśmiałowskiMotoyoshi KobayashiBostan NazishMouhamad KhoderBotao WuMouhcine FadilBouafia AbderrhmaneMounir Mohamed Salem-BekhitBouayad NoureddinMountasser DoumaBouchaib IhssaneMourad A. M. Aboul-SoudBouraoui IlahiMourad KharbachBoyko G. TsyntsarskiMousa GhaemyBoyu ZhangMoustafa DarwishBożena MuszyńskaMoustafa GabrBożena TyliszczakMousumi DebnathBožidar ČobeljićMozaniel OliveiraBradley S. SimpsonMozaniel Santana De OliveiraBrahim AissaMozhgan HosseinnezhadBrahim BouizgarneMrinal BhattacharjeeBrandon Peter Lucke-WoldMrutyunjay JenaBranimir PavlićMubasher HussainBranislav MarkovićMucong LiBranislava Srđenović-ČonićMudasir DarBranka PetkovićMudasir RashidBranka PilicMudassir Hussain TahirBrelid HaraldMuhamad MustafaBrenda A. Silva-EspinozaMuhamad Ramdzan BuyongBrenda L. Sánchez-GaytánMuhammad A. AlsherbinyBrendan M. DugganMuhammad AamirBrenden W. HamiltonMuhammad Abdul HaqBreno De Mello SilvaMuhammad Adly Rahandi LubisBrett PatersonMuhammad AfzaalBrian E. LoveMuhammad Ahsan AltafBrian LeonardMuhammad Ali ButtBrian RolczynskiMuhammad Ali SikandarBrice WilsonMuhammad Altaf NazirBrienne N. SeinerMuhammad ArifBrigida AlfanoMuhammad ArshadBrigitte HantuschMuhammad AshfaqBrigitte MarianMuhammad Asif NawazBrînduşa Alina PetreMuhammad Asif Zahoor RajaBritt Marianna MaestroniMuhammad AzeemBruce A. McGregorMuhammad Bashir KhanBruce MilthorpeMuhammad Bilal SadiqBrun PaolaMuhammad Faisal ManzoorBruna CarbasMuhammad HarrisBruna LarattaMuhammad HumayunBruna SinjariMuhammad IbrahimBruno Bueno-SilvaMuhammad ImranBruno K. RobbsMuhammad Imran KhanBruno Leite SampaioMuhammad Imran TousifBruno Mattia BizzarriMuhammad Iqbal KhanBruno Oliveira Da Silva DuranMuhammad Junaid RaoBruno PaganoMuhammad Kashif ShahidBruno TassoMuhammad KhalidBruno TirilliniMuhammad Khalid KhanBryan BreyfogleMuhammad ManshaBryan FullerMuhammad MohsinBryan WeiMuhammad MujtabaBryan WongMuhammad NadeemBuck RogersMuhammad Nadeem HassanBuddhadev PurohitMuhammad NasirBuddolla Anantha LakshmiMuhammad NawazBudsaraporn NgampanyaMuhammad NomanBulat SaifutdinovMuhammad RaheelBundet BoekfaMuhammad RiazBunleu SungthongMuhammad SaleemBurcu OzdenMuhammad SalmanBushra UzairMuhammad SaqibBwalya A. WitikaMuhammad Shahzad AslamByung Seok MoonMuhammad Shahzad KamalByungheon LeeMuhammad SohailC. Michael GreenliefMuhammad Sohail ZafarCaio H. FrancoMuhammad Syafiq Hazwan RuslanCaisheng WuMuhammad TahaCălin CăinapMuhammad TaherCălin Gabriel FloareMuhammad UmairCalin SteindalMuhammad Usman HameedCallistus BvenuraMuhammad Wahab AmjadCamelia Elena LuchianMuhammad Waheed IqbalCamelia SmarandaMuhammad Waqas ZubairCamelia VizireanuMuhammad Waseem MumtazCameron MackerethMuhammad ZafarCameron WeberMuhammad Zubair IsrarCamila D. Madeira CamposMuhammad ZulfajriCamilla D. BuarqueMuhammad Zulhaziman Mat SallehCamilla Fabiano De FreitasMuhammed A. AcikgozCamilla MatassiniMuhammed KüpeCamilla NoèMuhammed Naseem AkhtarCamille LatoucheMuhammet Ali GundesliCamille MaloufMuhammet İrfan AksuCamillo La MesaMuhd Danish-DanielCamilo Dias Seabra PereiraMuhhamad SajidCandelaria Tejada-TovarMuhittin KulakCândida Teixeira TomazMükerrem KayaCanzhong LuMukesh KumarCarbone AnnaMukesh MeenaCarine MaalikiMukti Ram PaudelCarl ErbMukund SrinivasanCarl FisherMukund TantakCarl P. TrippMukundam VangaCarla GentileMunawar NaylaCarla Lucia EspositoMunehito AraiCarla MaregaMunish AhujaCarla NunesMu-Ping NiehCarla SardoMurali DamaCarles FoguetMuralikannan MaruthamuthuCarlo CamerlingoMurat BingulCarlo DiaferiaMuriel MathonnetCarlo GattiMurni Nazira SarianCarlo GonzatoMurray B. IsmanCarlo PuntaMurugesan VelayuthamCarlo Travaglini-AllocatelliMusa KamaciCarlos A. EslavaMuslum DemirCarlos A. Martínez-HuitleMustafa HititCarlos Aguilar-PérezMustafa SaltıCarlos Alberto CañasMustafa SevindikCarlos Alberto Lobato TapiaMustafa TurkyilmazogluCarlos AmenabarMustapha Cherkaoui MalkiCarlos AmeroMustapha Umar ImamCarlos Andres Galan-VidalMutee MurshedCarlos AppoloniMuthaiah AnnalakshmiCarlos AydilloMuthaiah ShellaiahCarlos CarlesiMuthiah ChellappandianCarlos CaroMuthu ThiruvengadamCarlos Correa-NettoMuthuchamy NallalCarlos E. S. BernardesMuthuramalingam PandiyanCarlos E. VelasquezMuthuramalingam PrakashCarlos Eduardo De Araújo PadilhaMuthusamy SanjivkumarCarlos Eduardo Molina-GuerreroMuthusankar EswaranCarlos EscottMuttalip GündoğduCarlos F. MartinoMuzamil KhatriCarlos F. TorresMuzzamal HussainCarlos FernandezMyeong-Cheoul ChoCarlos Gamarra-LuquesMykola ObushakCarlos GarcíaMyong Yong ChoiCarlos GeraldesMyoung Chong SongCarlos H. G. MartinsMyoung-Hwan ParkCarlos H. HirokiMyra VillarealCarlos HernandezMyriam RichaudCarlos Hernando GómezMyroslav HolovkoCarlos Iván Delgado-NieblasMyung-Bok WieCarlos J. CarrascoMyunggi YiCarlos J. MinahkMyungsoo JooCarlos LagosN. BhagyaCarlos LimaNa HuCarlos López-GómezNa NiuCarlos M. AfonsoNaaman Francisco Nogueira SilvaCarlos Martins GomesNabil ElsheeryCarlos MoyaNabil SaffajCarlos O. Castillo-AraizaNada ĆujićCarlos RosasNada M. MostafaCarlos Sierra FernándezNada SmigicCarlos VilaNada VahcicCarlota Gómez-RincónNadège Lubin-GermainCarlotta FigliolaNadejda HorchidanCarlotta FranciaNader ShehataCarmela TripaldiNadezhda A. GolubkinaCarmelo García PintoNadezhda BokachCarmelo La RosaNadezhda SachivkinaCarmelo SgarlataNadezhda VchisloCarmen BarbaNadhira Bensaada LaidaniCarmen Belén MolinaNadia AkramCarmen Cornelia GaidauNadia CalabrisoCarmen CuadradoNadia GuajardoCarmen DobreaNadia IntanCarmen Garcia-JaresNadia J. Jacobo-HerreraCarmen GherasimNadia LicciardelloCarmen Lidia ChitescuNadia Pellegrini-MoïseCarmen Martin CorderoNadiia I. GumerovaCarmen Méndez HernándezNadiia MamekaCarmen Molíns-LeguaNadine MartinetCarmen O. Meléndez-PizarroNadir BettacheCarmen RaclesNadja FreundCarmen Reyes MateoNadja HellmannCarmen SaezNae-Cherng YangCarmen SavinNaechul ShinCarmen TeránNagappagari Lakshmana ReddyCarmina SirignanoNagaraju KerruCarmine BuonocoreNagaraju SakkaniCarmine GaetaNagendra ChauhanCarola SchulzkeNagendra Kumar KaushikCarole AiméNagham Mahmood AljamaliCarolina ConstantinNaglis MalysCarolina FloresNagula ShankaraiahCarolina FontanaNahid KaisarCarolina Letícia Zilli VieiraNahla A. AyoubCarolina LorenteNahla AlamoodiCarolina MarquesNahla S. El-ShenawyCarolina MascayanoNahum Medellín-CastilloCarolina Pérez ReyesNa-Hyung KimCarolina S. VinagreiroNail M. ShavaleevCaroline Costa MoraesNaila BobyCaroline Weinstein-OppenheimerNaira SahakyanCarrie HouseNajam ShakilCarsten MüllerNajmeh KarimianCassamo Ussemane MussagyNak Cheon JeongCassandra E. Deering-RiceNalladurai KaliyanCassandra TerryNallani SatyanarayanaCassiano Felippe Gonçalves-de-AlbuquerqueNallaperumal NallamuthuCastejón NataliaNallely Rivero-PerezCastorena-Torres FabiolaNalok DuttaCatalin ConstantinescuNan LiCătălin MaximNana Kwadwo BiritwumCatalin TutaNana MaCatalin ZahariaNanae HarashimaCatalina Natalia Cheaburu YilmazNanang MasruchinCatalina Rivas-MoralesNancy S. YounisCatarina Dias de AlmeidaNanda Kumar GhoshCatarina Dos SantosNandakumar KalarikkalCatarina L. SilvaNandini SarkarCatarina SeabraNanjangud V. Anil KumarCaterina DuranteNanna Hjort VidkjærCaterina PeggionNaokazu IdotaCaterina RodríguezNaokazu KanoCaterina SturtzelNaoki HaraguchiCatharine EsterhuysenNaoko IkutaCatherine DavisNaoto YagiCatherine Kejia XuNaoufal El HachlafiCatherine Leigh BroadhurstNaoufel Ben HamadiCatherine M. CookNarayana NageshCathrine Sumathi ManoharNarayanankutty ArunaksharanCato BredeNarciso M. GarridoCecil StushnoffNarendra Pal Singh ChauhanCécile PolgeNaresh DuvvaCecilia B. DobreckyNaresh KumarCecilia BattistelliNareshbabu KamathamCecilia ColettiNarmina O. BalayevaCecilia FaraloniNarottam AchyaCecilia Lucia CaglieroNarsimha MamidiCecilia PozziNarumol JariyasopitCecilia RomanNashwa HagagyCecilia SpedalieriNasim Nsooudi NsooudiCeferino Carrera FernándezNasir KhanCelia BustosNasir RasoolCelia DuceNassim Ghaffari-Tabrizi-WizsyCelia Maria SoaresNatacha MerindolCélia Marisa SilveiraNatale PerchiazziCéline CholetNatale R. MussoCeline HenoumontNatalia A. ChumakovaCelso Dario RamosNatalia AlzaCemil AydoğanNatalia AtamasCerasela Elena GîrdNatalia BelosludtsevaCésar C. Leyva-PorrasNatália C. HomemCesar Leobardo Aguirre-MancillaNatália Cruz-MartinsCésar Mateo Flores-OrtízNatalia Di PietroCésar OliveiraNatalia GladkikhCesar SaldiasNatalia GudzCesare CamettiNatalia ManousiCesare FredaNatália MartinsCesare GrazioliNatalia Mayumi InadaCesare UmetonNatalia Mazur-PanasiukCezar ComanescuNatalia MiklášováCezary CzaplewskiNatalia Nowacka-JechalkeCezary CzosnekNatália ParoulChadin KulsingNatalia PorotnikovaChairul IrawanNatalia StanekChaisak ChansriniyomNatalia SzałajChaithanya I. N. KiranNatalia V. BelkovaChaiwat PrapainainarNatalia Viktorovna ZagoskinaChaiyavat ChaiyasutNatalia WilkeChakraborty AbhijitNatalija PolovicChakrit SriprachuabwongNataliya A. IvanovaChak-Shing KwanNataliya KolosovaChalida NiamnuyNataliya M. SelivanovaChamila GunathilakeNataliya MaksimchukChan Kyung KimNataliya SaninaChanat AonbangkhenNataliya Ye. StasyukChanchal Deep KaurNatallia V. DubashynskayaChandan SinghNatalya MirzoyevaChandra MadasuNatarajan ArunadeviChandra Sourabh AzadNatarajan SuganthyChandrabose SelvarajNatasa JokovicChandrasekhar YadavalliNatasa KalogiouriChandrashekhar VoshavarNataša NastićChang ChenNataša Poklar UlrihChangan GengNatasa SiminChangbo ZhuNataša Z. TomićChanggu LeeNatella EnukashvilyChanglei XiaNatércia BrásChangli ZhaoNatércia TeixeiraChangling HuNatesan ThirupathiChang-Myung OhNathalia PinheiroChangqing SuNathalie JullianChang-Shen LinNathan P. ManesChangsheng ZhangNathaniel GilbertChangsun KangNathaniel HepowitChangwei HsiehNathdanai HarnkarnsujaritChangwu ZhengNatsuo UedaChangzhou YuanNattapol TangsuphoomChan-Jung KimNattawut BoonyuenChan-Yen KuoNattawut OsakooChao DongNauman Rahim KhanChao LiNavadeep ShrivastavaChao LiuNavaneet ChaturvediChao NiuNavaz KharazianChao WangNaved MalekChao XieNaveed Iqbal RajaChao YanNaveen MahantiChao ZhanNavid Jubaer AyonChaobo HuangNavid OmidifarChao-Hui FengNavin KumarChao-Lien LiuNavin Kumar DevarajChaouiki AbdelkarimNavneet KaurCharalambos FotakisNavneet RaiCharalambos KanakisNawaf Al-MaharikCharalampos KarantonisNay Myo WinCharalampos SiristatidisNayra A. M. MoussaCharin TechapunNazanin MojtabaviCharise Dallazem BertolNazim HasanCharlène GadaisNazim NassarCharles BrennanNazim SekerogluCharles D. RiceNazli FarajzadehCharles F. ManfulNazmul KarimCharles HocartNdzondelelo BingwaCharles L. B. MacdonaldNeal HickeyCharles LochenieNebojsa IlicCharles OkpalaNebojša NedićCharlotte GaviardNebojsa PavlovićCharng-Cherng ChyauNebojsa PotkonjakCharu GuptaNecla PehlivanCharu Lata SharmaNecmi DegeChatchakorn EurtivongNectarios VidakisChathura AbeywickramaNeda MilinkovicChathura SuraweeraNeda O. ĐorđevićChatragadda RameshNeda S. GavarićChayanaphat ChokradjaroenNedasadat Saadati ArdestaniCheang KhooNedeljko ManojlovićChe-Chien ChangNeeladrisingha DasChedia AouadhiNeelima MahatoChee Kong YapNeena BediChen ChaoNeeraj JainChen Ching-TaiNeeraj Kumar SethiyaChen I. YangNeeraj Singh ThakurChen SongNeetu DayalChen ZhaoNeetu TalrejaCheng ChenNehal ElsherbinyCheng ChengNehru Viji SankaranarayananCheng HuangNeil FitzgeraldCheng JinNeith PachecoCheng LiuNejib KasmiCheng WangNela MalatestiCheng Wang (China)Neli Kinga OlahCheng Wang (USA)Nelson Pérez GuerraCheng YanNemanja P. TrišovićCheng ZhangNemanja RajčevićCheng ZouNemanja TeslicChengcheng LiNemat AliCheng-Chien WangNemesio Villa-RuanoChenghan LiNenad IgnjatovicCheng-Hsin ChuangNenad JoksimovićCheng-I LeeNenad L. VukovicCheng-Kang ChiangNerdy NerdyChengliang XiaoNerea Jiménez-MorenoChengpeng ChenNerilson Marques LimaCheng-Yang HuangNermeen YosriCheng-Yen KaoNeshat AbdollahCheng-Yu WangNesrein M. HashemCheng-Zhang LuNesrin FayekChengzhen LiuNestor Gutiérrez-MéndezChen-Hao WangNeven ZarkovicChennaiah AndeNevenka RajicChensheng MaNewman OsafoChenyang XieNg Eng PohCheonwoo JeongNgbolua Koto-te-NyiwaCherednichenko KirillNgoc N. NguyenCherng-Yuan LinNgoc Quyen TranCheryl ConoverNgu TrinhChetan PaliwalNguyen H. H. PhucChetna MohabeerNguyen Minh DucCheuk Lun LiuNguyen Quang TrungChhanda BoseNguyen Quoc Khanh LeChi Chiu WangNguyen Quoc KhuongChia Ming ChangNguyen Thanh LeChia-Che ChangNguyen Trong TuanChia-Chen ChangNguyen Van QuanChia-Ching LiawNhan T. NguyenChia-Hsien FengNiall M. H. McLeodChia-Hung LeeNiccolò Traverso ZianiChiaki KurodaNicha PuangmalaiChiaki Nagai-OkataniNicholas HengChian-Hui LaiNicholas J. PerainoChia-Ning YangNickolas D. CharistosChiara BorsariNiclas SolinChiara BrulloNicola BorboneChiara CarazzoneNicola D'AntonaChiara Da PieveNicola Di FidioChiara De LucaNicola MicaleChiara GioncoNicola SangiorgiChiara La TorreNicola SimoneChiara LambruschiniNicolai A. AksenovChiara Lo PortoNicolas Adrian ReyChiara MartinelliNicolas GischChiara PelosiNicolas LepareurChiara PortesiNicolas MuzzioChiara RinoldiNicolás OteroChiara Roberta GirelliNicolas PapaïconomouChiara SinisgalliNicolas RollandChiara ValoriNicole Remaliah Samantha SibuyiChia-Ying LiNicole TeuschChidambaram ThamaraiselvanNicoleta RaduChien-Chung YangNicoleta-Aurelia ChiraChien-Hsing LeeNicolò LagoChien-Jui HuangNicolò RiboniChien-Min ChiangNicolo TonaliChie-Shaan SuNidal JaradatChih Hung LoNidhal Ben KhedherChih Yuan KoNidhi SinglaChih-Chieh WangNidia Casas-ForeroChih-Feng HuangNidia Cristiane YoshidaChih-Liang WangNiël Van WykChih-Ming MaNiels Christian NielsenChih-Yao HouNiels HansenChi-Ming WongNihad AdnanChin Wei LaiNihal ÇetinChinedu Prosper AnokwuruNii Korley KorteiChing-Fa YaoNiina PiippoChing-Feng WengNika PavlovićChing-Hao TengNikiforos AlygizakisChing-Kuo LeeNikita ChaudharyChing-Yi TsaiNikita MoiseevChin-Kun WangNikita N. SviridenkoChinmay SatamNikita TsvetovChinmaya MahapatraNiklas PakkasjarviChinmaya MutalikNikola BurdzhievChin-Ping KungNikola GeskovskiChintan KapadiaNikola Hadzi-PetrushevChin-Yiu ChanNikola MajorChi-Ping LiNikola MarakovićChirag ChopraNikola MaravićChirag N. PatelNikola SakačChis Adriana AureliaNikola StojanovicChisato KinoshitaNikolai I. GeorgievChi-Tien ChenNikolai V. RostovskiiChittranjan SinhaNikolai V. TsvetkovChiwai KanNikolaos ChalmpesChi-Yu LuNikolaos LabrouChlodwig FranzNikolaos LougiakisChonghang ZhaoNikolaos N. LourosChoo Hock TanNikolaos NazirisChoon Young KimNikolaos NikolaouChris DouvrisNikolaos PistamaltzianChris J. AnagnostopoulosNikolaos PolitakosChris JonesNikolaos RemmasChristel NeutNikolay A. PyataevChristian BaillyNikolay GorshkovChristian BarbatoNikolay KozyrevChristian BleilevensNikolay MakishaChristian BrandNikolay Melik-NubarovChristian BüchterNikolay RodkevichChristian Chapa GonzálezNikolay TkachenkoChristian Espinosa BustosNikolay Ul'yanovskiyChristian HanselNikolay VassilevChristian JelschNikolett Kállai-SzabóChristian JungnickelNikoletta G. NtalliChristian KrambergerNikolina Maček HrvatChristian LehmannNikolina RusenovaChristian LudwigNikunjkumar PatelChristian Mück-LichtenfeldNilesh ChoudharyChristian R. Encina-ZeladaNilesh NirmalChristian RoumestandNilesh R. ChodankarChristian SonnendeckerNilesh SharmaChristian StockmannNileshi SarafChristian TantardiniNima SaeidiChristiana M. NeophytouNima SanadgolChristiane Fernandes HornNimbe TorresChristina S. VakhNina FilipczakChristina SakarikouNina FincurChristina ZalaruNina KravetsChristine ArbourNina PastorChristine EnjalbalNina RembiaŁkowskaChristine S. GibhardtNing GanChristine SaluzzoNing LiChristo SevovNing QuChristoph AlexiouNing ZhangChristoph KlenkNinganagouda PatilChristoph MüllerNingbo GongChristophe AmpèNing-Ning LiChristophe BliardNingning ZhangChristophe CurtiNiranjan ParajuliChristophe ErneuxNirmal MazumderChristophe FliedelNisar Ul KhaliqChristophe OgueyNishad Thamban ChandrikaChristopher C. KalteneckerNishant KumarChristopher FennellNitesh K. NandwanaChristopher HarrisonNitesh MittalChristopher J. ZerioNiti YongvanichChristopher PöhlmannNitin MehtaChristopher WaltonNitinkumar Satyadev UpadhyayChristopher WassonNives GalićChristos KontoyannisNiyaz MahmoodiChristos PappasNiyaz Mohammad MahmoodiChristos RitzoulisNiyazi BulutChrysanthi KalloniatiNizar B. AlsharifChryssoula DrouzaNobuaki HigashiChrystelle Bancon-MontignyNobuaki OkumuraChuan LiNobuhiro IshidaChuan-Fa ChangNobuyoshi MatsumotoChuanfeng ChenNobuyoshi MoritaChuang-Chieh LinNoe Baruch TorresChuangye YaoNoel PereraChuanlong GuoNoelia BajalesChuanqi PengNoelia Caballero-CaseroChuanxiong NieNoelia CasaresChuanyin XiongNoélia DuarteChuda ChittasuphoNoelia Geribaldi-DoldanChun HuNoelle S. WilliamsChun Kit Benjamin TongNoemí Echegaray SuárezChun Sang HongNoha El ZaharChun TangNoha F. SwilamChun-Chi ChenNoha I. ZiedanChunchia ChengNohad GreshChung Chen TsaoNoor Baity SaidiChung F. WongNoor WahabChung WongNoora BarzkarChung-Chih WuNooshin SohaniChung-Chun WuNoppawan MoralesChung-Fu HuangNor QhairulChung-Hsin WuNora MimouneChung-Jung LiuNorazlianie SazaliChung-Kuang LuNorbert JuxChung-Shin YuanNorbert LihiChunhong YangNorberto FarfanChunhuan ZhangNorhan K. Abd El-AzizChun-Hui ChiuNorhana JusohChunjie XiaNorihiro InagakiChunlei LiNorihisa KatoChunlin DengNoriko SaitoChun-Lin LeeNorio NakataChunxi ZhangNorio SogawaChunyi WuNoriyuki YamagiwaChunyu WangNoriyuki YamaotsuChutima LimmatvapiratNorizah RahmanChu-Young KimNorma Francenia Santos-SánchezCíntia SoaresNorma Salazar-LópezCinzia FabriziNorma-Aurea Rangel-VázquezCinzia GarofaloNorman H. L. ChiuCinzia La RoccaNorman RatcliffeCinzia PaganoNorrie PearceCinzia TavaniNorzita NgadiCiobanu Anne-MarieNosomi Taniwaki NoemiCiro AbbondanzaNoto Susanto GultomCiro IsidoroNour E. A. Abd El-SattarCiro MenaleNour Eddine Es-SafiCitlalli TiburcioNour F. AttaiaClaire AllenNoura DosokyClaire StockerNoureddine El AouadClara MarcoNoureddine ElboughdiriClara Maria Della CroceNoureddine IssaouiClara MeanaNourhéne Boudhrioua-MihoubiClara S. B. GomesNovriyandi HanifClarence T. T. WongNozomu SuzukiClarissa P. FrizzoNqobile MasondoClastinrusselraj I. SathishNripendra Vikram SinghClaude CharvetNugraha SuyatmaClaude DidierjeanNuno Dinis AlvesClaude JolivaltNuno NengClaudia Avitia-DomínguezNuno ValeCláudia BotelhoNunzia ScottiClaudia CapitiniNunzio CardulloClaudia ChilomNunzio TuccittoClaudia ContiNur CebiClaudia ContiniNur Hanisah AzmiClaudia Contreras-CeledónNur Izyan Wan AzeleeClaudia Di GiacomoNuray AtesClaudia FotiNurgul K BakirhanClaudia FuentealbaNurhasni HasanClaudia GenoveseNurhayat TabancaClaudia Giovagnoli-VicuñaNuria AceroClaudia HonischNuria Esturau-EscofetCláudia MajoloNuria FiolClaudia MancaNúria López-VinentClaudia Muhle-GollNuria SotomayorClaudia RibeiroNurkhalida KamalClaudia RiccardiNurlaila IsmailCláudia RochaNurul Shazini RamliClaudia ScatignoOana Andreea CojocaruClaudia Segal-KischinevzkyOana CadarClaudia SorbiOana Cristina ŞeremetClaudia Valentina CimpoiuOana Dragos-PinzaruClaudia YanezOana Emilia ConstantinClaudio A. TerrazaOana Viorela NistorClaudio AmovilliOana-Maria BolduraClaudio CaprariObaid AfzalClaudio CeconeOberdan FerreiraClaudio FerranteOctavian Tudorel OlaruClaudio FerrariOctavio Elizalde SolisClaudio LarosaOctavio RonceroClaudio LombardelliOdilia Pérez-CamachoClaudio MedanaOgadimma D. OkaguClaudio ParraOhad MazorClaudio SantiOinam Romesh MeiteiClaudio TrapellaOkio HinoClaudio VillaniOksana DudarkoClaudiu MorgovanOksana KomovaClaudiu N. LunguOksana V. SalomatinaClaus CornettOksana V. SytarClemens LamberthOksana ZininaClemens ZwergelOla A. TarawnehClementina ManeraOla SundmanCleofe PalocciOladipo FolorunsoCleverson Diniz Teixeira De FreitasOladipupo Odunayo OlatundeCodruta SoicaOlcay Kaplan InceColin C. SeatonOlcay Y. JonesColin CampbellOldrich LapcíkColin J. SucklingOle VangCömert Önder FerahOleg Ivanov KolodiazhnyiConcepción C. GonzálezOleg KovtunConcepción Pérez-LamelaOleg Lvovich PolyanskyConcetta De StefanoOleg M. DemchukConcetta Di NataleOleg N. StarovoytovConcetta ImperatoreOleg PetuhovConcettina La MottaOleg ShevchukCong PengOleg SilyukovCongjiang CaoOleg Vladislavovich LevinCongwei WangOlena IvashchenkoCongyue PengOlesya V. StepanenkoConrad RizalOlga A. BulavchenkoConstain H. SalamancaOlga A. MostovayaConstantine StalikasOlga A. RozentsvetConstantinos K. ZacharisOlga B. Alvarez-PerezConstantinos M. MikelisOlga D. ZakharovaConstantinos TsiafoulisOlga E. GlukhovaConstanza CardenasOlga EscuderoConsuelo CelestiOlga GornikCorina Anca SimionOlga GrygorievaCorina AramăOlga I. YarovayaCormac MurphyOlga JanouskováCornel BaltaOlga KaltsaCornel CobianuOlga Karolina KosakowskaCornelia MirceaOlga LebedevaCornelia WiegandOlga LodochnikovaCorneliu CojocaruOlga LuzinaCorneliu Ioan OpreaOlga M. SharonovaCorneliu TanaseOlga MartinCornelius KraselOlga MokhodoevaCorrado PelaiaOlga MysiukiewiczCorrado RizziOlga N. SolovjevaCosimo CardellicchioOlga PavlovaCosta BachasOlga ProtchenkoCostantino CiallellaOlga Rojo-PovedaCostas TsioptsiasOlga TarasovaCristian FurauOlga TsivilevaCristian M. O. LéporiOlga V. KaravaiCristian MartinezOlga WesołowskaCristian MunteanuOlga Yu BakulinaCristian PetcuOlgica NedicCristian PîrvuOlha MykhailenkoCristian SandovalOlimpio MonteroCristian ScheauOliver KühnCristian SimionOliver TrappCristian VicentOliver TuševskiCristiana GonçalvesOliver ZerbeCristiana RadulescuOlivera DjuragicCristiane AguiarOlivera GalovićCristiane CananOlívia R. PereiraCristiane KalinkeOlivier GalangauCristiane Yumi Koga-ItoOlivier GoncalvesCristiano E. Rodrigues ReisOlivier LaprévôteCristiano J. De AndradeOlivier LebelCristina AchimOliviero CarugoCristina B. HebedaOlja ŠovljanskiCristina Burrola-AguilarOlubanke Olujoke OgunlanaCristina CalejaOluwatoyin A. AdelekeCristina CantaruttiOmar Guzman-QuevedoCristina CapatinaOmar M. SabryCristina CarvalhoOmar SarheedCristina Cebrián ÁvilaOmayma A. EldahshanCristina Cebrián-TarancónOmbretta RepettoCristina CortiOmid AkhavanCristina ForzatoOmolola E. FayemiCristina Juan GarcíaÖmür AydınCristina MaccalliniOndrej CehlárCristina Mariana UrituOndrej JurcekCristina MartinezOndrej KurkaCristina Martin-SabrosoOndrej VanekCristina MihaliOnur GuvenCristina MinnelliOnur YilmazCristina Montiel-DuarteOon Ein ChernCristina NastasaOriana Lo ReCristina OlgasiOriana TrubianiCristina OlivaroOriele PalumboCristina P. R. XavierOrnella AbollinoCristina Pérez-RiveroOrsolya Zsirka-FónagyCristina PrietoOsama Gomaa MohamedCristina SchreinerOsama SaberCristina SoaresOsama YounisCristina TodaşcǎOsamu KanieCristina TortoliniOsamu SatoCristina TruzxiOsamu TakahashiCristina TubaroOsamu UrakawaCristina-Maria TrandafirescuOscar A. Lenis-RojasCristobal MirandaOscar ForeroČrtomir PodlipnikOscar Herrera-CalderonCruz Miguel CendanOscar NunezCrystal S. ShinOscar RomeroCsaba HetényiOscar VerhoCsaba MagyarOskars PlatnieksCsaba VáradiOsman ErkmenCui LiOsmar Antonio Jaramillo-MoralesCulleré LauraOsmindo R. Pires JúniorCustódia GagoOsvaldo Aquines-GutiérrezCvijeta Jakobusic BralaOtilia BobisCybelle FutalanOtilia CulicovCyril ColasOttorino OriCyril FersingOussama El-KadriCyril GobinetOvidiu Cristian OpreaCyril NicolasOvidiu OnigaCyrille SabotOvidiu TitaCzesław PuchalskiOxana SerovaDa SunOzair AlamDa Wei TengOzohanics OliverDaeha JoungPablo BlancoDaeho ParkPablo Breogán Sánchez VázquezDae-Shik SeoPablo EstevezDae-Sung LeePablo FuentealbaDagmara BaczyńskaPablo García-ManriqueDagmara StefańskaPablo HegelDagmara Wróbel-BiedrawaPablo Lopez-AlbarranDa-Hua WeiPablo MirallesDaiana ÁvilaPablo NogaraDaiki KuzuharaPablo PinachoDaisuke IshiiPablo Simón MarquésDaisuke MiyoshiPagadala Damodaram Kamala JayanthiDaisuke TodakaPäivi Mäki-ArvelaDaiva BaltriukienePál SiposDaizy R. BatishPalak PatelDalia Anwar HamzaPalanisamy RavichandiranDalia M. KopustinskienePalaniyandi KaruppaiyaDalia UrbonavičienėPalash SanphuiDalibor ŠatínskýPallabita ChowdhuryDalibor TiteraPaloma Álvarez-MateosDaliborka Koceva KomlenićPaloma Sánchez TorresDamián Álvarez PaggiPan PantziarkaDamian GawelPan WangDamien BoscPan ZhangDamien BourgeoisPanagiota-Kyriaki RevelouDan Cristian VodnarPanagiotis PoulopoulosDan GamrasniPanagiotis ZoumpoulakisDan HuPandiaraj ManickamDan Jae LinPandurangan RamarajDan QiPaniz JasbiDan RosuPankaj BharmoriaDan YuanPankaj KumarDana BejanPankaj Kumar SinghDana Carmen ZahaPankaj PandeyDana CucuPankaj SharmaDana DvoranovaPankul DhingraDana Luca MotocPaola AngeliniDana MarcinčákováPaola BarrajaDanail PavlovPaola CartaDancho DanalevPaola CeteraDandan GuoPaola ChecconiDanh C. VuPaola CicatielloDania OlmosPaola CostanzoDanica Bajuk-BogdanovićPaola De LucaDaniel A. HorkePaola De PadovaDaniel A. KleierPaola Di MatteoDaniel Alexandre NeuwaldPaola FaraoniDaniel ArandaPaola FiniDaniel ArcanjoPaola FossaDaniel Asunción-AlvarezPaola GrenniDaniel BaeckerPaola RappelliDaniel BarragánPaola RognoniDaniel CosanoPaola SemeraroDaniel CozzolinoPaola Serena GinestraDaniel DemarquePaola StoriciDaniel ErrandoneaPaola UngaroDaniel Fernández-GonzálezPaolo BertoncelloDaniel Fernández-QuirozPaolo ConflittiDaniel GarbePaolo GuglielmiDaniel GeisslerPaolo PellegrinoDaniel Glossman-MitnikPaolo SgarbossaDaniel H. González MaglioPaolo ZardiDaniel J. V. A. Dos SantosPapp Lajos AttilaDaniel KamińskiParameswara Rao VuddandaDaniel Kam-Wah MokParamita BasuDaniel KracherParandaman ArathalaDaniel M. KaminskiParaskev T. NedialkovDaniel M. KetchaParaskevas D. TzanavarasDaniel M. KirganParaskevi HeldinDaniel MagnoneParaskevi MitliagkaDániel NemesParasuraman PavadaiDaniel ObenchainPardeep SinghDaniel Ortega-CuellarParmar AmbikaDaniel P. OttoParracino AntoniettaDaniel PaynePartha BasuDaniel Q. TanPartha Sarathi AddyDaniel RamsbeckParthasarathi RamakrishnanDaniel RealParthasarathi SubramanianDaniel SalamangoParvaneh PakravanDaniel SzulczykParvesh WadhwaniDaniel TietzePascal ColosettiDaniel VertedorPascal DegraceDaniel WerzPascal MarchandDaniel ZałuskiPascal MartinDaniela Amidžić KlarićPascal PuechDaniela AntonovaPascal QuernerDaniela BordaPaško KonjevodaDaniela BuchaimPasquale D'AngeloDaniela Cajado-CarvalhoPasquale LincianoDaniela Caldeira CostaPasquali Matheus Augusto De BittencourtDaniela CarlisiPasqualina D'UrsiDaniela Cristina BergerPasquina MarzolaDaniela DragomanPászti ZoltánDaniela Elena PopaPatcharee PripdeevechDaniela GiacomazzaPatrice A. MarchandDaniela HanganuPatrice VanelleDaniela IstratiPatricia Álvarez-FitzDaniela Joyce Trujillo SilvaPatricia ConcepciónDaniela KunecováPatricia E. EdemDaniela MarascoPatricia García HerreraDaniela Maria TanasePatricia J. LardoneDaniela MeloPatricia Jovičević-KlugDaniela MiricescuPatricia LeglerDaniela PerronePatricia LemaDaniela PredoiPatrícia M. ReisDaniela R. TruzziPatrícia MáximoDaniela RagoPatricia MoralesDaniela RussoPatricia Rayas-DuarteDaniela Simina StefanPatrícia Ribeiro PereiraDaniela UbialiPatrícia SeverinoDaniela ValensinPatrícia Vitorino MendonçaDaniela Valeria MinieroPatricio Orellana-PalmaDaniele ContiniPatricio RamosDaniele FranchiPatrick BradshawDaniele GarcovichPatrick CarberryDaniele MantionePatrick D. FischerDanielle Duanis-AssafPatrick DansetteDanielle TwilleyPatrick HerrDaniil A. LukyanovPatrick LegembreDaniil IvanovPatrick LévêqueDaniil OlennikovPatrick VideauDanijel D. MilinčićPatrizia BovolinDanijela BarićPatrizia FormosoDanijela Bursac-KovacevicPatrizia MessiDanijela KrsticPatrizia RossiDanijela Ristić-MedićPatrizia ScafatoDanijela SavicPatrizia StefanelliDanil DybtsevPatrizia TrovalusciDanila B. VasilchenkoPatroklos VareltzisDanilo Escobar-AvelloPatrycja GodlewskaDanilo Grünig Humberto Da SilvaPatrycja KleczkowskaDanilo Justino CarastanPatrycja MakośDanilo StojanovićPau Loke ShowDanilo Trabudo Do AmaralPaul B. SavageDanilo VonaPaul Baruk Zamudio-FloresDanish IqbalPaul BogdanDanka BukvickiPaul DalhaimerDanmeng LuoPaul Fleurat-LessardDanny J. J. WangPaul G WentholdDanny ManorPaul G. HiggsDanuta BranowskaPaul H. M. HarrisonDanuta Kołożyn-KrajewskaPaul JanssenDanuta MiedzińskaPaul JoyceDanuta NowickaPaul KirkbrideDanuta Rusinska-RoszakPaul KlineDanuta SilukPaul KosmaDanyu YaoPaul RöschDaozong XiaPaul ScheierDapeng ChenPaul ThomasDapeng WangPaul ValleDarcey WaymentPaul VasosDarcy BurnsPaula AranazDaria GrzywaczPaula BorrajoDaria MelnikovaPaula BrancoDarija Lušić VukićPaula BrandãoDarinka FakinPaula C. B. PaígaDario AchaPaula C. CastilhoDario KremerPaula DietrichDario MizrachiPaula Fernandes De AguiarDarius KviklysPaula Fraga-GarcíaDarius MilčiusPaula GarcíaDariusz FydrychPaula Homem De MelloDariusz JedrejekPaula LudovicoDariusz KarczPaula M. FerreiraDariusz KruszkaPaula M. MarcosDariusz KulusPaula NabaisDariusz Maciej PisklakPaula OssowiczDariusz PiesikPaula PereiraDariusz ŚwietlikPaula PintoDariusz SzczepanikPaula RodriguesDariusz T. MlynarczykPaula SilvaDarko ModunPaulina KoczurkiewiczDarko P. AšaninPaulina Latko-DurałekDarko VelićPaulina MertowskaDarón I. FreedbergPaulina MisztakDarren RiddyPaulina NowickaDarya KlyamerPaulina ŻelechowskaDarya NovopashinaPaulina ŻeliszewskaDarya TarbeevaPaulo Alexandre Mira MourãoDarya TishkevichPaulo AlmeidaDas PoushaliPaulo CarvalhoDasha MihaylovaPaulo De Tarso Cavalcante FreireDasheng LuPaulo José Gomes CoutinhoDasiel Borroto-EscuelaPaulo PiresDave C. GarnerPaulo Ribeiro-ClaroDavid BotequimPaulo Riceli RibeiroDavid BrookPaulo Roberto H. MorenoDavid ChatenetPaulo SantosDavid Chávez-FloresPaulo Sérgio CerriDavid ChelazziPaulo Victor Sgobbi De SouzaDavid ClosePavan KumarDavid DixonPavel A. DemakovDavid ElorriagaPavel AbramovDavid EvansPavel Anatolyevich NikolaychukDavid GaulPavel AvramovDavid H. CalhounPavel B. DrašarDavid HaložanPavel BanerjeeDavid HarmanPavel DemakovDavid HerzogPavel KhramtsovDavid HunterPavel MokrejšDavid J. BrayPavel NesterenkoDavid J. LundyPavel PadnyaDavid KinderPavel PolischukDavid Lee PhillipsPavel ReitermanDavid LinPavel S. GribanovDavid M. BarberPavel SpirinDavid Medina-CruzPavel UrbánekDavid NelsonPavel V. KomarovDavid OwensPavel Vladimirovich DolganovDavid P. SmithPavel VolynskyDavid PadrosaPavel ZelenikhinDavid PamiesPavle TančićDavid PuettPavlína BasařováDavid R. CalabresePavlina DolashkaDavid RamonetPavlos PandisDavid Rodríguez San MiguelPavol TisovskýDavid S. P. CardosoPawan Kumar DubeyDavid Salinas-TorresPawan Kumar RaghavDavid Sancho-CantúsPawan MalhotraDavid SchultzPaweena PrapainainarDavid SemerilPaweł ChmielarzDavid Shan-Hill WongPawel JarkaDavid ŠilhaPaweł KafarskiDavid StegnerPawel KonieczynskiDavid T. StuartPaweł KowalczykDavid Vicente-ZurdoPawel KrupaDavid W. WoodPawel KwiatkowskiDavide BarrecaPaweł LenartowiczDavide ClematisPaweł LisDavide De AngelisPaweł ŁukowskiDavide DeodatoPaweł M. WróbelDavide LauroPawel MozejkoDavide MoiPaweł OlczykDavide PapurelloPaweł P. WłodarczykDavide RaineriPawel PohlDavide RavelliPaweł PomastowskiDavide RovinaPawel PopielarskiDavit JishkarianiPaweł RamosDavit PipoyanPawel RodziewiczDavood KharaghaniPaweł ŚliwaDavor MargetićPaweł ŚwitDawid PrzystupskiPayam ZahediDawid StefaniukPayam ZarrintajDawid ZychPedram AzariDaxin LiangPedro Aguilar-ZarateDayong ShiPedro AraujoDayun YanPedro BrugarolasDe GongPedro CarvalhoDeana B. AndrićPedro Cerezal-MezquitaDebabrata MandalPedro Cosme RedondoDebabrata SenguptaPedro D. VazDebanjana GhoshPedro Damián Loeza-LaraDebasish BandyopadhyayPedro De Sousa GomesDebasish PaulPedro De-la-TorreDebasmita SahaPedro E. RamirezDebipreeta BhowmikPedro F. ArceDebora Decote-RicardoPedro FernandesDebora FontaniniPedro Ferreira-SantosDebora NapoliPedro GuiraoDebora ParisPedro Henrique FerriDeborah QuaglioPedro J. S. B. CaridadeDechao HuPedro LebreDeeb TaherPedro M. E. ManciniDeep KalitaPedro M. MartinsDeepak KumarPedro Martín-AcostaDeepak LokwaniPedro MateusDeepanwita BanerjeePedro Nuno Sousa SampaioDeependra SinghPedro Ortega-GudiñoDeepika AwasthiPedro Raposeiro Da SilvaDeepika SinghPedro SilvinoDeepti SharmaPedro TavaresDeiana RomanPedro Y. S. NakasuDeisy Alessandra DrunklerPedzisai MakoniDejan AgićPegah JohanssonDejan JeremicPei LiangDejan MilenkovićPeifeng SuDejan PljevljakušićPeigen RenDejan StojkovićPeihua MaDejun ZhangPei-Hui LinDelia HorhatPeijiang LiuDelia Mihaela RaţăPeilong LuDelia MunteanPeipei HanDelia TitPeirong ChenDemeter TzeliPeisheng ZhangDemetra GiuriPeiwu QinDemetres D. LeonidasPeiyi WuDemetrio Jackson Dos SantosPeiying ShiDemetrios ArvanitisPeiyu CaiDemetrios KouretasPeiyuan QuDenan WangPeizhou LiDenis A. BabkovPenelope Merino-MontielDenis BaranenkoPenelope PelczarDenis BazhinPeng ChenDenis FuentealbaPeng DuDenis KarimovPeng GaoDenis S. KudryavtsevPeng LiDenis V. ChachkovPeng TaoDenis V. SudarikovPeng XieDenis VinnikPeng XueDenise Fernandes CoutinhoPeng ZhangDenise M. SelegatoPeng ZhaoDenitza ZgurevaPengchong XueDeniz Tanıl YücesoyPengfei DingDennis BrownPengfei MaDennis P. CloughertyPenghao WangDeping LiPenghua ShuDequan ZhangPengpeng LiuDerek BussanPengxiang ChangDerenik KhachatryanPensak JantrawutDer-Sheng ChanPer Amstrup PedersenDerval Dos Santos RosaPer Bendix JeppesenDerya OsmaniyePer Jr GreisenDer-Yuan ChenPer MalmbergDer-Yuh LinPerihan Yolci ÖmeroğluDesire Molina AlcaidePeriyasami GnanaprakasamDesirée LeistenschneiderPeriyasami GovindasamiDesislava Georgieva TenevaPerla D. MaldonadoDevegowda V. GowdaPershina OlgaDevendra SinghPerumal VivekanandhanDevi Prasan OjhaPetar KassalDevin W. McBridePetar T. TodorovDewi Selvia FardhyantiPeter A. AjibadeDeyanira Ángeles-BeltránPeter Achunike AkahDeyao LiPeter AntalDezhong YangPeter B. StathopulosDezsö HorváthPeter BaiDhananjay YadavPeter BališDhananjayan VasuPeter BencsikDhanasekaran SugapriyaPeter BerdonosovDhanasekaran VikramanPeter BrizaDhaneswar PrustyPeter C. GainesDharmendra Kumar MeenaPeter ColterDharmendra Kumar YadavPeter DrogeDharmeshkumar PatelPéter FodorDhiraj SrivastavaPeter GorelkinDhiraj VyasPeter HammerDhruba PathakPeter HauserDi LiuPeter J. ChoiDi Zhang (China)Péter KeglevichDi Zhang (USA)Peter KubatkaDiana AmanteaPeter M. TolstoyDiana Andreia Tavares PintoPeter MccarthyDiana AntalPeter O'ConnellDiana B. Muñiz-MárquezPeter OelschlaegerDiana Balogh-WeiserPeter ParsonsDiana BecerraPeter PascoeDiana Camelia NuţǎPeter PolčicDiana CheshmedzhievaPeter RoseDiana CiolaciuPeter S. SherinDiana Cláudia PintoPéter SiposDiana DiasPeter Sol ReinachDiana DonatellaPeter ŠtackoDiana LopesPéter SzentesiDiana MironovaPeter TolgyessyDiana ObistioiuPeter TomčíkDiana RabaPeter Van WalsumDiana SilvaPeter VeranicDiana V. AleksanyanPetia DatcharyDiandou XuPetko AlovDíaz-Real Jesús AdriánPetr ČeslaDíaz-Reval IrenePetr KozlíkDibya PandaPetr KubáňDidem KartPetr PrausDidem ŞöhretoğluPetr S. FedotovDidier BoquetPetr StadlbauerDidier DesmaëlePetr TomekDidier TouraudPetr TůmaDiego AntonioliPetr YeletskiDiego CaprioglioPetr ZacekDiego F. TiradoPetra DunkelDiego Fernando Garcia-DiazPetra HellwigDiego Fernando Paladines-QuezadaPetra ImhofDiego GarciaPetra KosutovaDiego La MendolaPetra LoveckáDiego Martin BustosPetra MatićDiego MoralesPetra SchlingDiego OlivieriPetra VaskoDiego PaschoalPetras PrakasDiego QuirogaPetre IonitaDiego Romano PerinelliPetri KilpeläinenDiego SampedroPetro E. PetridesDiego TesauroPetro Onys'koDievart PascalPetros GiastasDigamber RanePetros P. PetrouDijana JelićPetrova BoryanaDijana Žukovec TopalovićPetru JitaruDijendra Nath RoyPetru NegreaDikdik KurniaPeyman EbrahimiDilbar AibibuPeyman GholamiDileep KumarPhei Er SawDilep Kumar SigalapalliPhila RaharivelomananaDiletta AmiPhilipp HoneggerDiletta BalliPhilipp OrekhovDilip DepanPhilippe LecomteDilshad HussainPhilippe RocheDilyana PanevaPhilippe TrensDimitar BojilovPhitchayapak WintachaiDimitra Hadjipavlou-LitinaPhiwayinkosi V. DludlaDimitra SazouPhuong H. NguyenDimitra Z. LantzourakiPhuong Mai LeDimitri BogdanovskiPiedad Gañán RojoDimitrina Zheleva-DimitrovaPier Carlo RicciDimitrinka NikolovaPier Franco LattanziDimitrios A. AnagnostopoulosPier Giorgio SchiaviDimitrios KanelisPiera ValentiDimitrios StagosPierluigi RevegliaDimitrios TsimogiannisPiero BiancoDimitris GorpasPiero FranceschiDimitris P. MakrisPierpaolo ScaranoDina AhmedPierre ChaurandDina DeynekoPierre Le GendreDina MurtinhoPierre Van AntwerpenDinesh BarakPierre Van CutsemDinesh BhalothiaPierre-Adrien PayardDinesh C. SabarirajanPierre-Antoine BonnetDinesh IndurthiPierre-Eric CamposDinesh KumarPierre-Yves RenardDinesh Mani TripathiPierre-Yves SacréDinesh MittalPiersandro PallaviciniDinesh PatelPięt MateuszDinesh PathakPiétrick HudhommeDinesh RokayaPietro BalloneDineshkumar SengottuveluPietro GalinettoDing WangPietro Paolo UrgegheDing WeiPietro RussoDingbo LinPietro ScicchitanoDingtao WuPietro TedescoDini IrenePilaiwanwadee HutamekalinDinithi PeirisPilar Campíns-FalcóDino AquilanoPilar Carranza-RosalesDionizy CzekajPilar ZafrillaDionysia PapagiannopoulouPimonsri Mitraparp-ArthornDioselina Álvarez BernalPinaki P. MisraDipak Kumar SahooPinalysa CosmaDipak MaityPınar ŞenDipankar AshPing DongDipankar RoyPing LiDipti KanabarPing SuDirk BenderPing ZhuDirk H. OrtgiesPing-Chung KuoDirk J. E. M. RoekaertsPing-Hsun WuDirk MontagPing-Jyun SungDirk SchaumloffelPinglin LiDirk TischlerPingrong WeiDirk W. LachenmeierPingyuan WangDitta UngorPiotr A. GaudenDivya UtrejaPiotr BełdowskiDivyesh VaradePiotr BorowikDjenisa RochaPiotr BorysiukDmitrii KalininPiotr CyganikDmitrii L. ObydennovPiotr CysewskiDmitrii MazhukinPiotr F. J. LipińskiDmitrii S. BolotinPiotr GaudenDmitriy MakarovPiotr KamedulskiDmitriy ShcherbakovPiotr KaszynskiDmitriy ShurpikPiotr KulinowskiDmitriy YambulatovPiotr KuryloDmitry A. LoginovPiotr KuśDmitry A. ShulgaPiotr KuśtrowskiDmitry A. TikhonovPiotr LukasiakDmitry FursaPiotr LulińskiDmitry GruzdevPiotr MakDmitry I. SharapaPiotr MatczakDmitry Mikhailovich NazarovPiotr MinkiewiczDmitry NamgaladzePiotr OkińczycDmitry NoldePiotr Oskar CzechowskiDmitry PashchenkoPiotr PanekDmitry PelageevPiotr SalachnaDmitry S. KosyakovPiotr SkurskiDmitry ShulgaPiotr StefanowiczDmitry TsymbarenkoPiotr SugierDmitry VainchteinPiotr ŚwiątekDmitry VlasovPiotr SzcześniakDmitry YakimchukPiotr SzwedaDmytro NesterovPiotr WlaźDo Yeung YoonPiotr ZygmanskiDoga BilicanPiroska Szabó-RévészDoha AboubakerPiumi LiyanageDolores HernanzPius Oziri UkohaDomenica CapassoPiya TemviriyanukulDomenica D'EliaPiyatip KhuntayapornDomenica MangieriPiyush GuptaDomenica MarabelloPiyusha PagareDomenico FrancoPlacido MineoDomenico GadaletaPlacido NeriDomenico IacopettaPlamen StoyanovDomenico MajolinoPloenthip PuthongkingDomenico MontesanoPo-An ChenDominic AugustinePo-Chih YangDominic E. L. OngPo-Hsien LiDominico A. Guillén-SánchezPol Ribes-PleguezueloDominik CinčićPolavarapu Bilhan Kavi KishorDominik FuhrmannPoliana Macedo SantosDominik KmiecikPolina A. DeminaDominik KoszelewskiPolina TyubaevaDominik LungerichPolonca TrebseDominika LewandowskaPompilia Ispas-SzaboDominika SzkatułaPonlapat RojnuckarinDominique AgustinPonmalai Gounder KolandaivelDominique KouaPonnulakshmi RajagopalDominique ManikowskiPoonam AgarwalDominique Serge Ngono BikoboPooya LahijaniDomitilla MandatoriPornchai RojsitthisakDonald SikazwePornsiri PitchakarnDonatas ŠneiderisPosa SudhakaraDonatella BulonePo-Shen PanDonatella Degl'innocentiPostek EligiuszDonatella LoruPoul Erik HansenDonatella PietrellaPo-Wen ChenDonato Di VenerePrabhat SinghDong ChenPrabodh SatyalDong Hyuk ChunPrabu Kumar SeetharamanDong June AhnPrachurjya DuttaDong WangPradeep KumarDong Wook ShinPradeep RaiDong XinPradhumna Mahat ChhetriDongcheng ChenPradip Kumar SukulDongdong Wang (Singapore)Prafull SalviDongdong Wang (USA)Pragneya DemeDongfang ZhouPragya TiwariDongfeng ZhangPragya VermaDongHao WangPrakash G. KshirsagarDongheon LeePrakash KarmakarDonghwa KimPrakash LingasamyDongkuk LeePrakash PanduranganDongliang QiuPramit ChowdhuryDongrong XiaoPramod K. PrabhakarDongsheng LeiPramod Kumar SinghDongting YuePramod YadavDongwei ZhangPramodkumar P GuptaDongwoo LimPramote ChumnanpuenDonovan ArguetaPranav NawaniDóra K. MenyhárdPranay SrivastavaDora MehnPranjali NaikDora MelucciPrasad ChavanDora ValenciaPrasad ParchuriDoreen PalsgrovePrasad RasaneDoretta CuffaroPrasad TrivediDorian HanaorPrasanna Kumar SanthekadurDorian MigońPrasanna NeelakantanDoriana TedescoPrasanna RamaniDoriano FabbroPrasanna VaddiDoriano LambaPrasannavenkatesh DuraiDoris Mangiaracina BenbrookPrasanth PuthanveetilDorota Cais-SokolińskaPrashant KumarDorota ChudobaPrashant Kumar GuptaDorota DerewiakaPrathama MainkarDorota GałkowskaPrathap SomuDorota Grabek-LejkoPratibha KumariDorota KrasowskaPratibha PandeyDorota LatekPratibha RaniDorota NieoczymPratik SenDorota OlenderPratim Kumar ChattarajDorota PiotrowskaPraveen P. SinghDorota PorowskaPrawez AlamDorota TumialisPredrag KojićDorota WarmińskaPredrag PetrovićDorota WianowskaPreecha PhuwapraisirisanDorota WojniczPreeyanuch SangtrirutnugulDorota WrześniokPrem Chandra PandeyDorota ZającPrem KushwahaDorota ŻelaszczykPrem Pratap SinghDorota ŻyżelewiczPřemysl LubalDorrain Yanwen LowPrimerova OlgaDouglas Henrique PereiraPrince ChawlaDouglas Maya-MilesPriscila GubertDouglas RayniePriscila Pereira Silva-CaldeiraDouglas S. AuldPriscilla Barbosa Sales De AlbuquerqueDouglas Vieira ThomazPriscilla Rocio-BautistaDouglas W. BousfieldPritam GangulyDoungporn AmornlerdpisonPritam Kumar DikshitDragana D. Vasić AnićijevićPritam SadhukhanDragana DabićPritika RamharackDragana I. TamindžijaPriya BharateDragana Mutavdžić PavlovićPriya MurugasenDragana Vasic AnicijevicPriya Ranjan SahooDragica SpasojevićPriyadarshini KachrooDrago BesloPriyanka BhattDragos HorvathPriyanka RayDragos Paul MihaiPriyanka SrivastavaDrashya SharmaProdromos SkenderidisDriss CherqaouiProkopios GeorgopanosDriss OusaaidPrzemysław CzeleńDror MalkaPrzemysław DanekDuangjai TungmunnithumPrzemysław DopieralskiDuc Huy NguyenPrzemysław DorożyńskiDuclerc Fernandes ParraPrzemyslaw GagatDung Nguyen TrongPrzemysław KołodziejDunja SamecPrzemysław KosobuckiDunya L. Al-DuhaidahawPrzemysŁaw ł. ZalewskiDunyaporn TrachoothamPrzemysław NiedzielskiDuong NguyenPrzemyslaw PlonkaDuraipandiyan VeeramuthuPrzemysław PłonkaDusan ChorvatPrzemyslaw SiejakDušan DimićPrzemyslaw SitarekDušan MalenovPu DuanDusan RuzicPukar KhanalDusan VelkovicPulak BhushanDushyant BarpagaPullapanthula RadhakrishnanandDusica SimijonovicPurushothaman NatarajanDzintra AtstājaQaisar MaqboolEbenezer AnnanQamar AbbasEbenezer C. NnadozieQamar Abbas SyedEbube OyekaQamar AhmedEder RomeroQazi Mohammad Sajid JamalEdgar JacobyQi CaoEdgar O’RearQi ChenEdgar R. Pastene-NavarreteQi CuiEdgars ElstsQi QiaoEdi SyafriQi ShenEdina AvdovićQi WangEdina PandurQi YuEdirisingha Dewage Nalaka Sandun AbeyrathneQian GouEdison OsorioQian LiEdit CsapóQian WangEdit É. FarkasQian XuEdith HochhauserQian ZhangEdith Ponce-AlquiciraQianchun ZhangEdivaldo Herculano Correa De OliveiraQiang FuEdna Regina AmanteQiang LiEdoardo LongoQiang WangEdson SantanaQiang XuEdson SilvaQiang YuEduarda MendesQiang ZhangEduard-Marius LungulescuQiang ZhuEduardo BonavitaQiangqiang LiEduardo Borges De MeloQiangsong WangEduardo Carvalho LiraQiangzhong ZhaoEduardo CostaQianmin WangEduardo Costa De FigueiredoQianqian WangEduardo De Jesus OliveiraQiao YangEduardo E. MiróQiaosheng PuEduardo F. FrancaQicheng FengEduardo Fernández-MartínezQichun ZhangEduardo Festozo VicenteQien YangEduardo Fuentes-LemusQifeng FuEduardo García-VerdugoQihao WuEduardo González-ZamoraQihui WangEduardo GuzmánQiliang LuEduardo Molina-JijonQilin ZhangEduardo Morales-SánchezQin FuEduardo Rivadeneyra-DomínguezQinfeng ZhuEduardo ValarezoQing ChenEdurne Avellanal ZaballaQing JinEdvaldo Vasconcelos Soares MacielQing YangEdward ChernQingbin CuiEdward H. SharmanQinghe GaoEdward HadaśQinghe ZhangEdward J. FoxQinghui WangEdward LoukopoulosQingli DongEdward MunteanQinglin ShengEdward SachetQingming HuangEdwin PalacioQingrong PengEdyta KucharskaQingsheng WangEdyta MakuchQingsheng ZhaoEdyta MałachowskaQingtong ZhouEdyta Nalewajko-SieliwoniukQingwen ZhangEdyta SłupekQingxin YaoEdyta SymoniukQingyan XuEfat JokarQingye ZhangEfigenia Montalvo GonzálezQingyi TongEfraím ReyesQingyu ZhouEftichia KritsiQingzhong LiEgidio ViolaQinheng ZhengEgoitz AstigarragaQinru SunEgor VerbitskiyQinzhe WangEhab El-HarounQishi SongEhsan Bahojb NoruziQiu YingkunEhsan KarimiQiubao OuyangEhsan Ullah MughalQiucheng ChenEhsan ZeimaranQiufeng HuangEiji KubotaQiuhui PanEike ReichQiujin ZhuEiko Nakagawa ItanoQiumin LuEitoyo KokubuQiushi YinEka PrasedyaQixuan ZhengEkaterina A. KnyazevaQiyin ChenEkaterina AstrovaQiyuan HeEkaterina BartashevichQize ZhangEkaterina D. ObluchinskayaQuaiser SaquibEkaterina Fedorovna KolesanovaQuan HongquanEkaterina GeorgievaQuan JinEkaterina KozlovaQuan-Bin HanEkaterina NaumenkoQuang Minh NgoEkaterina S. MenchinskayaQuanguo HeEkaterina ShchegravinaQuanqing ZhaoEkaterina Stepanova-KhramtsovaQuentin K. KaasEkaterina SukhovaQuintas-Granados Laura ItzelEkaterina Yu BezsudnovaQun CaoEkaterina YurchenkoQun TangEkram AliasQun ZhouEkram HossainQurban AliEkta MenghaniR. Daniel LittleEl Hassan SakarR. Martin VabulasEl Hassane AnouarR. N. V. Krishna DeepakEladia M Peña-MéndezR. Rama SureshEladl EltanahyRaanan CarmieliElaine Cruz RosasRabab MohammedElba MaurizRabbani SyedEldrin D. ArguellesRabia AyubEleftherios K. AlissandrakisRachela G. MilazzoElena A. Kral’kinaRachid SkoutaElena AlekseevaRachid TouzaniElena B. AverinaRachna KapoorElena BeloglazkinaRachna VermaElena BonciuRadek MalikElena BorodinaRadek PohlElena CanellasRadi JishiElena CariatiRadim HavelekElena ChikhirzhinaRadim KučeraElena ChugunovaRadivoj ProdanovićElena CicheroRadmila JankovićElena CiniRadomir JasinskiElena Coyago-CruzRadosław BarczakElena De MarchiRadoslaw KitelElena DilonardoRadoslaw KujawskiElena F. ShevtsovaRadosław MrówczyńskiElena Falqué LópezRadosław PodsiadłyElena FilonovaRadosław StarostaElena G. VolkovaRadostina GeorgievaElena GiordanoRadovan BilekElena GirichRadovan BurešElena GolubevaRadu C. RacovitaElena I. RyabchikovaRadu Silaghi-DumitrescuElena KirilovaRadu TamaianElena LenciRadu-Cristian MoldovanElena LeshchenkoRaees KhanElena LeychenkoRaelene CowieElena LilkovaRaewyn TownElena LucentiRafael Baltiérrez-HoyosElena M. Peña-VázquezRafael BaptistaElena M. Sánchez-FernándezRafael Camacho-CarranzaElena Maria VaroniRafael ChinchillaElena Martínez-CarballoRafael CuestaElena MilanesiRafael Das ChagasElena MilevaRafael DefendiElena PiniRafael Guillén-BejaranoElena RomanoRafael J. BorgesElena TomsikRafael LucenaElena V. GrachovaRafael NunesElena V. KolobkovaRafael R. CastilloElena V. NikitinaRafael Torres-MendietaElena V. ProskurninaRafał FrańskiElena V. SvirshchevskayaRafał JanuszewskiElena V. UspenskayaRafal KozdrachElena Vladimirovna BoldyrevaRafał MilanowskiElena Yu TupikinaRafał NadulskiElena ZelepugaRafal RakoczyElena-Luiza EpureRafal StrekowskiEleni A. RekkaRafał Tytus BrayEleni BozinouRafał WawrzyniakEleonora MarianRafał WienchEleonora MazzuccoRafał WysokińskiEleonora PalaganoRafeeya ShamsEleonora SocoRaffaele FrazziEleonóra SpekkerRaffaele MarroneEleonora TruzziRaffaele RiccioEleonora VatamanRaffaella CatalanoEleonora VirgilioRaffaella ComitatoEleuterio ÁlvarezRaffaella MancusoElfi KrakaRaffaello PapadakisElhassan AmaterzRafi UllahElia GruesoRafig GurbanovEliana Dell'olmoRafik SaddikEliana Fortes GrisRaghavendhar R. KothaEliana Sousa Da SilvaRaghavendra KikkeriEliane CollaRaghavendra Rao PasupuletiEliane De Oliviera SilvaRaghunath O. RamabhadranEliane NoronhaRaghunath Reddy AnuguElias SakellisRaghunath SinghElias ZagattoRaghvendra Pratap SinghElif YildizRagnar BjornssonElina PeltomaaRagul GowthamanElina Vuorimaa-LaukkanenRahul D. JawarkarElisa AleRahul K. GuptaElisa BiazziRahul KavtheElisa BindaRahul ShingareElisa BrasiliRahul Shubhra MandalElisa CairrãoRai Campos SilvaElisa NutiRaimund WinterElisa RobottiRainer SchmidtElisabet Navarro-TapiaRaj KarthikElisabeta BotezRaj Kishori LalElisabeta-Irina GeanaRaj KumarElisabeth JacobsenRaj Kumar AryaElisabeth PinartRaj Kumar MongreElisabetta CanettaRaja Asad Ali KhanElisabetta FinocchioRaja VeerapandianElisabetta GiudiceRajalakshmanan EswaramoorthyElisabetta MariniRajapandiyan PanneerselvamElisabetta OrlandiniRajasekhara NerimetlaElisabetta TorregianiRajat BhattacharyaElise Des LignerisRajat WaliaEliseo HernandezRajeev KumarEliud Avier HernándezRajeev SinglaEliyahu DremencovRajeev YadavEliza BlicharskaRajender BoddulaEliza WolskaRajendra BhadaneElizabeth Carvajal-MillanRajendran SaravananElizabeth ErasmusRajendran SatheeshkumarElizabeth FernandesRajendran SelvakesavanElizabeth GilchristRajesh GanesanElizabeth HillardRajesh HaldharElizabeth NeumannRajesh P. ShastryEl-Kazafy Abdou TahaRaji GovindanEllas SpyratouRaji Paul GrewalElliot R. BernsteinRaji SundararajanElma VukoRajib Kumar MitraElmsellem HichamRajinder SinghElmuez A. DawiRajith Singh MananElnur E. GarayevRajiv PeriakaruppanÉlodie BlancoRajkumar BandiElodie ChoqueRajkumar SrinivasanEloisa SardellaRajkumar Sunil SinghElsa F. VieiraRajni VermaElsa Margarida GonçalvesRajnibhas Sukeaw SamakradhamrongthaiElsa SultanovaRaju Senthil KumarEl-Saadony MohamedRakesh Barani KumarElsayed Mohammed ElsayedRakesh GangulyEl-Sayed Ramadan El-SayedRakesh K. SindhuElsherbiny ElsherbinyRakesh K. TiwariElson Santiago De AlvarengaRakesh Kumar BachhetiElumalai SanniyasiRakesh PuttreddyElvira E. ShultsRalf BlosseyElvira Kovač-AndrićRalph PuchtaElwira ChrobakRaluca StanElyahb Allie KwizeraRam DattElzalina R. SoaresRam Hari DahalElżbieta Bartoszak-AdamskaRam Swaroop BanaElżbieta BrzezińskaRama Chandra PradhanElżbieta BudziszRama Kant DubeyElżbieta GrządkaRamachandra Reddy DonthiriElżbieta KarlińskaRamachandran PrakasamElzbieta KlewickaRamachandran SrinivasanElżbieta Łodyga-ChruścińskaRamachandran VinayagamElżbieta Lorenc-KociRamadhansyah Putra JayaElzbieta PachRamakanta LamichhaneElżbieta Radzymińska-LenarcikRamalingam AnantharajElżbieta Studzińska-SrokaRamalingam GopalElzbieta SzaconRaman PachaiappanEl$\overset{`}{z}$bieta TomaszewiczRamanaskanda BraveenthElżbieta WojaczyńskaRamar ThangamEmad A. AhmedRamasamy Prem KumarEmad Abdul-Hussain YousifRamchandra B. PodeEmad F. NewairRamesa BhatEmad RadwanRamesh ChingleEmad S. GodaRamesh KatariaEman E. MahmoudRamesh KumarEman M. OthmanRamesh MudududdlaEman MehannaRamesh MukkamalaEman S. NossierRamesh PudakeEmanuel Hernández-NúñezRamesh S. YadavaEmanuela BerrinoRameshthangam PalanivelEmanuela Calcio-GaudinoRami MhannaEmanuela EspositoRamin Rahmani AhranjaniEmanuela TrovatoRamin YousefiEmeka B. OkekeRamith RamuEmel OzRamón Esteban-RomeroEmerson José VenancioRamón Pedro ArtiagaEmil PaluchRamón Peña-GarciaEmil RudolfRamon Róseo Paula Pessoa Bezerra De MenezesEmili CidRamona D'AmicoEmilia DrozłowskaRamona DanacEmilia FisicaroRamona IseppiEmilia FuriaRamona SuharoschiEmilia KlimaszewskaRamos PatricioEmilia MiraRamune ZurauskieneEmilia P. CollarRan ChenEmilia Pers-KamczycRan HongEmiliano BediniRan WangEmiliano LaudadioRana Abdul ShakoorEmilija SvircevRana Muhammad AadilEmilija ZdravevaRandal S. StahlEmilio Álvarez-ParrillaRandall MaplesEmilio San FabianRandy NelsonEmilio Viñuelas ZahínosRanganathan BabujanarthanamEmilios K. DimitriadisRani BushraEmiliya V. NosovaRanier Enrique SepúlvedaEmily BaileyRanjeet DhokaleEmily CaponeRanjeet Kumar MishraEmily GraffRanjit DeEmily PadhiRanjith Kumar RajamaniEmina SherRaphaël E. DuvalEmirhan NemutluRaphael HorvathEmma Adriana OzonRaphaël LabruèreEmma SolaRaphael PlassonEmmanouil D. TsochatzisRaphael StollEmmanouil P. BenisRaquel BridiEmmanouil TsochatzisRaquel De Cássia Dos SantosEmmanuel AubertRaquel De Melo BarbosaEmmanuel BourdonRaquel EchavarriaEmmanuel M. PapamichaelRaquel G. SoengasEmmanuel RichaudRaquel GarciaEmmanuel Silva MarinhoRaquel TorrijosEmmanuelle BraudRasa PauliukaiteEmoke PallRasangi WimalasingheEmre KarakusRasha AwniEnade Perdana IstyastonoRasmané SemdeEncarnación Ruiz RamosRatana BanjerdpongchaiEncina M. González MartínezRataya LuechapudipornEndang LukitaningsihRatchadawan AukkanimartEndarto Y. Wardhono‬Rathi Devi-Nair GunasegavanEndler BorgesRathinam BalamuruganEnéas KonzenRathinam RajaEneli HaerkRathinamoorthy RamasamyEner SemihRathinasabapathi PrabhakaranEngy ElekhnawyRatna Sudha MadempudiEnnio CoccaRatul DasEnnio ProsperiRauf AbdurEnnio ZangrandoRaul ChiozzoneEnoch A. MensahRaúl Colorado-PeraltaEnol LopezRaúl Díaz-MolinaEnrica RosatoRaul FigueroaEnrico BodoRaúl López FernándezEnrico CarusoRavan A. RahimovEnrico MarsiliRavi Chand BollineniEnrico RagniRavi K. ShuklaEnrico Vito PerrinoRavi KumarEnrique BlázquezRavi Kumar CheedaralaEnrique Domínguez-ÁlvarezRavi Kumar SharmaEnrique González-VergaraRavi S. LankalapalliaEnriqueta AnticóRavi YadavEnsanya Ali Abou-NeelRavichandran KollarigowdaEnshirah Da'naRavikumar K. JimmidiEnzo CadoniRavikumar PatelEnzo SpisniRavindra ThakkarEpameinondas LeontidisRaviraj ShindeEphrem EngidaworkRaviraj VankayalaErben JakubRavish ChoudharyErcan BursalRay LuoErdal YabalakRaya-González JavierEren ŞahinerRayudika Aprilia Patindra PurbaErgun KayaRazieh RazaviEri MatsubaraRaziyeh AkbariEric AbenojarRazmaitė VioletaEric AndrianasoloRazvan BoiangiuEric DefrancqReaz ChowdhuryEric GloaguenRebar AbdulwahidEric HayRebeca AlvariñoEric Jim ThomasRebeca Betancourt-GalindoEric JohnsonRebeca Garcia-VarelaEric LabbeRebeca González-PastorEric MetzenRebecca J. WhelanEric W. PriceRebeka RudolfErica GentilinRecep GoekErica Georgina WilsonRecep LimanErich KleinpeterReda Ben MridErich MüllerReda El-ShishtawyErick César López-VidañaReddy Ramireddy ThrinathErick Cuevas-YañezReena V. SainiErick Gutiérrez-GrijalvaRegina Ngozi UgbajaErick Martínez HerreraRégis GuieuErick Paul Gutiérrez-GrijalvaRehab Mahmoud Abd El-BakyErick Sierra-CamposReham Hassan MekkyEricsson Coy-BarreraReiazul RehmanErik GoormaghtighReinaldo GeraldoErik VijgenboomReinhard DeneckeErika A. TaylorReinhard KremerÉrika C. AlvarengaReinhold P. LinkeErika CioneReinier HernandezErika Estrada CamarenaReisel MillánErika Liktor-BusaRemigijus IvanauskasErika López-ArribillagaRemigius ChizzolaErika Mariana PalmieriRemigiusz BąchorErika Ramos-TovarRemigiusz KowalikErika RangelRemigiusz ŻurawińskiErika RibechiniRemington PoulinErin G. Reed-GeaghanRemy DurandErin H. SeeleyRenan DanielskiErin M. LeitaoRenata CiccarelliÉriton Egídio Lisboa ValenteRenata Coelho Rodrigues NoronhaErjin ZhengRenata Jurišić GrubešićErman Salih IstıflıRenata Kelly MendesErmelinda MaçôasRenata ŁyszczekErna KaralijaRenata Maria SumalanErnesto Chigo-AnotaRenata MikstackaErnesto MataRenata Nurzyńska-WierdakErnesto QuesadaRenata OdžakErnesto ReverchonRenata Pietrzak-FiećkoErnesto ScoppolaRenata Salgado FernandesErnesto SotoRenata SiedleckaErol YildirimRenata TisiErsilia AlexaRenata Toczylowska-MaminskaErsin GülerRenata VadkertiovaErwei HuangRenata VardanegaEryk WolarzRenata WłodarczykErzsébet MernyákRenate GriffithErzsébet SándorRenato B. PereiraEsam Abdel-HadyRenato E. F. BotoEsam YahyaRénato FroidevauxEsaú Bojórquez-VelázquezRenaud GueroultEsin AkiRené BuchetEsko OksanenRené CsukEslam El-SawyRene HuberEsmaeel SharifiRené Massimiliano MarsanoEsmaeil Doustkhah HeraghRenee J. ChosedEsmerilda G. DelicadoRengasamy BalakrishnanEspadinha Margarida FlorindoRenhao DongEspen HansenReni KalfinEspérance DebsRenjie LiuEsraa M. BakhshRenjing HuangEsrat Jahan RupaRenjith ThomasEssa M. SaiedRenwang JiangEssam Mohamed ElsebaieRenyer CostaEstatira SepehrReshma B NambiarEsteban E. Urena-BenavidesRex FitzGeraldEsteban SáezRey David Vargas-SánchezEsteban UrriolabeitiaReynier SuardiazEstefanía Burgos-MorónReza ArjmandiEstela Cuevas-RomeroReza AzizianEster TriguerosReza HaghbakhshEsther Del OlmoReza KiaEsther Gomez MejiaReza Taheri-LedariEsther J. OcolaRhimi MoezEsus Antonio Morlett ChavezRiadh BadraouiEswar ShankarRiaz UllahEszter NemeskériRic De VosEszter SzabadosRicardo Costa CalhelhaEtelka TombáczRicardo DiasEtienne DucrotRicardo Fernández EscobarEtna Aída Peña-RamosRicardo González-RezaEttore VarricchioRicardo M. F. FernandesEugene Aleksandrovichn SalganskyRicardo Mallavia MarinEugene KatlenokRicardo Martins RamosEugene V. RadchenkoRicardo Massarico SerafimEugenia Fagadar-CosmaRicardo MendesEugénia PintoRicardo N. PereiraEulicio De Oliveira Lobo-JuniorRicardo Pinheiro De Souza OliveiraEulogio CastroRicardo Reyes-DíazEun Jeong ParkRicardo SantosEun Ju ShinRicardo SilvaEunho LeeRicardo Teobaldo CuyaEurico LimaRicardo Vivas-ReyesEva AltrockRicaurte RodriguezEva BaranovičováRiccardo AigottiEva DortaRiccardo LeardiEva FrankRiccardo NifosìEva GonzalesRiccardo PelosoEva M. Santos LópezRiccardo SalvioEva María García-FrutosRichard A. BunceEva PertileRichard BaybuttEva PluhařováRichard BodnarEva Ramón GallegosRichard ClarkEva RamosRichard D’ArcyEva Raudonytė-SvirbutavičienėRichard E. DavisEva RentschlerRichard HartshornÉva SzökőRichard HeynEva Tejedor-CalvoRichard HildnerEva VaňkováRichard HorrocksEva Von DomarosRichard HrabalÉva Zámboriné NémethRichard J. P. BrownEvagelos GikasRichard LivingstonEvaggelos KaselourisRichard LuduenaEvan TerrellRichard M. BeteckEvangelia TaraniRichard P. FahlmanEvangelia TzanetouRidha HorchaniEvangelia VouvoudiRigers BakiuEvangelos AlmpanisRigoberto Rios-EstepaEvangelos AxiotisRiham El-ShiekhEvangelos DaskalakisRihui CaoEvans N. NyabogaRijuta GaneshEvgenia GlukhovRika Indri AstutiEvgenia Korzhikova-VlakhRiku KawasakiEvgenii M. PlissRima D. AlharthyEvgeniia VikulovaRima HajjoEvgeniya BurkovaRimadani PratiwiEvgeniya I. KnerelmanRina PatramanonEvgeny ChupakhinRintu ThomasEvgeny ErmakovRio BoothelloEvgeny KundelevRita CarrottaEvgeny LodyginRita Cássia Superbi SousaEvgeny TrofimovRita CelanoEvgeny V. TretyakovRita De ZorziEvzen BouraRita HannisdalEwa BrzozowskaRita KadikovaEwa CiszkowiczRita Kartika SariEwa GlowinskaRita KissEwa HuszczaRita MarulloEwa JaskiewiczRita NastiEwa Juszyńska-GałązkaRita PachecoEwa KarwowskaRita Payan-CarreiraEwa KozłowskaRita SerranoEwa MajewskaRitesh RajuEwa Olchowik-GrabarekRitin SharmaEwa Olewnik-KruszkowskaRitu BarthwalEwa Ostrowska-LigȩzaRitu RavalEwa RaczyńskaRituraj PurohitEwa RutkowskaRivaldo NieroEwa SewerynRivka OfirEwa SkwarekRiyuan TangEwa SzymańskaRizwan WahabEwa TomaszewskaRobert A. F. M. ChamuleauEwa Żurawska-PłaksejRobert A. HarrisEwelina CichońRobert B. RuckerEwelina DondajewskaRobert CarleerEwelina HallmannRobert ElmesEwelina KowalczykRobert ForbesEwelina PiątczakRobert H. MachEylem Kulkoyluoglu CotulRobert J. BrownEylon YavinRobert J. ComitoEzequiel M. Vázquez-LópezRobert J. MishurEziuche Amadike UgboguRobert KawęckiEzzat KhanRobert Klaus HofstetterEzzat MarzoukRobert KołosEzzeldin MetwalliRobert LevensonFabian EzemaRobert M. ChristieFabián Fernández-LuqueñoRobert M. LafrenieFabian S. MengesRobert MückeFabian SchumacherRobert MusiołFabiane Valentini Francisqueti-FerronRobert NashFabiano S. RodembuschRobert P. HunterFabiao YuRobert P. SheridanFabio AricòRobert PowersFabio Arturo IannottiRobert R. LuedtkeFabio CarniatoRobert RusinekFabio CavaliereRobert Steven EsworthyFábio D. A. Aarão ReisRobert VianelloFábio De Souza DiasRobert VíchaFabio GasparriRobert WeinzierlFabio GosettiRobert ZaleśnyFabio Granados-ChinchillaRoberta BudriesiFabio MencarelliRoberta CazzolaFabio MiraldiRoberta DordoniFábio Neves Dos SantosRoberta GalariniFábio PassettiRoberta GaleazziFábio R. P. RochaRoberta IbbaFabio RizzoRoberta MoschiniFábio Rodrigues Ferreira SeivaRoberta RoccaFabio SalafiaRoberta SalaroliFabio SciubbaRoberto A. BotoFabio VaianoRoberto CampagnaFabio ZobiRoberto CastiglioneFabiola Sandoval-SalasRoberto ChiocchettiFabrício G. MenezesRoberto CiccorittiFabricio Maestá BezerraRoberto ContestabileFabrizio BarberisRoberto FabianiFabrizio CartaRoberto Fernandez-LafuenteFabrizio MachettiRoberto GomesFabrizio MontecuccoRoberto Gonzalez RodriguezFabrizio MurgiaRoberto GottiFabrizio VeticaRoberto LemusFabrizio VincenziRoberto LigroneFadia Victoria Cervantes DomínguezRoberto Lo GiudiceFadia YoussefRoberto Lo ScalzoFadime Aydin KoseRoberto MandrioliFadis F. MurzakhanovRoberto Martínez BeamonteFahad A. AlhumaydhiRoberto NegroFahad KhanRoberto RomeoFahad M. AlmutairiRoberto Romero-GonzálezFahad RashidRoberto SalcedoFaham KhamesipourRoberto ScaffaroFahd NasrRoberto ScatenaFahim AhmedRoberto SimoniniFai-Chu WongRoberto StellaFaisal AlzahraniRoberto VerucchiFaisal MukhtarRoberto Zamora BustillosFaisal RazaRoberts EglitisFaiyaz ShakeelRobin JoshiFaizul AzamRobin KinnelFakher ChabchoubRobin P. SagarFakir Shahidullah TareqRobin RajanFaliang LuoRobin WilganFang LiuRobinson Cortes-HuertoFangfang ZhongRobson Carlos AlnochFangrui ZhongRobson De FariasFangyuan ZhangRobyn L. PrueittFanica CimpoesuRoccaldo SardellaFanny MousseauRocchina PietrafesaFantin MarcoRocio Alejandra Chavez-SantoscoyFaraz SoltaniRocio B. DominguezFarid KhalloukiRocio Borges-ArgaezFarid MenaaRocío Meza-GordilloFarid MoeinpourRocío RedónFarid OrudzhevRodica Maricela AnghelFarida El-DarsRodica OlarFarman Ali KhanRodion V. BelosludovFarshad SohbatzadehRodney Edwin ShackelfordFarzan GholamrezaRodolfo Abarca-VargasFarzaneh KordbachehRodrigo Alves Soares CruzFarzaneh MahmoudiRodrigo BarderasFarzaneh MohamadpourRodrigo CasasnovasFarzaneh SabbaghRodrigo CruzFarzaneh TafviziRodrigo Herrera-MolinaFateh Mebarek-OudinaRodrigo Ligabue-BraunFatema BhinderwalaRodrigo MartinsFatemeh Farjadian FarjadianRodrigo RazoFatemeh Kazemi-LomedashtRodrigo Rollin-PinheiroFatemeh MirzaeiRodrigo Sávio PessoaFatemeh MokhtariRodrigo TorresFatemeh MovahediRodrigo ValenzuelaFatemeh RajabiRodrigues Chaves AndreaFatemeh RezaieRogelio Sánchez-VegaFatih DemirRoger AlbertoFatih SonmezRoger ChongFatiha BrahmiRogério Luis CansianFatima BentoRogerio R. Sotelo-MundoFatima CerqueiraRohan D. ErandeFátima De Cássia Oliveira GomesRohit MaharFatima El KhalloufiRok FinkFatima RivasRoksana MarkiewiczFatima Zahra MahjoubiRoland FischerFatimah KhalafRoland GoversFatin JannusRoland TengolicsFatma M. Abdel BarRolffy Ortiz-AndradeFatma Nil ErtaşRomain EychenneFausto Rubio AlonsoRomain ParentFauze AnaissiRoman B. LesykFauziah MarpaniRoman B. VasilievFavián TreulénRoman BegunovFawaz AldabbaghRoman GancarzFawzi Khoder-AghaRoman K. PuzanskyFawzi MahomoodalRoman LangFayaz AliRoman LysiukFayyaz Ur Rehman MuhammadRoman PaduchFazal RahimRoman PopielarzFazlin Mohd FauziRoman ShendrikFederica AureliRoman SvetogorovFederica BalzanoRoman SzostakFederica BlandoRoman V. MoiseevFederica BrunoRomana VulturarFederica ChiapporiRomeli BarbosaFederica CirilloRomeo RomagnoliFederica Federica CiuchiRomeo Theodor CristinaFederica FlamminiiRomualdo TroisiFederica GuffantiRonald A. LubetFederica ManninoRonald AndersonFederica MoracaRonald B MooreFederica PisaneschiRonald GarnerFederica RaucciRonald GustFederica SodanoRonald NelsonFederica TonoloRonaldo CozzaFederico BertiRonaldo Nascimento De OliveiraFederico CorelliRonan BureauFederico IacovelliRong ChenFederico Maria RubinoRong HuangFederico OlivieriRong XiangFederico PalazzettiRongguo RenFederico TottiRongjin SunFedor V. RyzhkovRoni NugrahaFedor V. SubachRonny MartínezFehmi DamkaciRoobanvenkatesh ThirumalaiFei HuangRoohallah Saberi-RisehFei LiuRoong Jien WongFei MaRoozbeh Abedini NassabFei PengRosa Anna MilellaFei WangRosa BellavitaFei YeRosa CalvelloFeilong LiRosa CarballoFeipeng YangRosa Colucci CanteFei-Ting HsuRosa Estrada-ReyesFelice GrandinettiRosa FontanaFelicia Gabriela GligorRosa GiuglianoFelipe ArretcheRosa Lebrón-AguilarFelipe FantuzziRosa M. De LederkremerFelipe López-SaucedoRosa Martha Pérez-GutiérrezFelix DitzingerRosa PerestreloFelix IacobRosa PurgatorioFélix Javier Jiménez-JiménezRosa RomeoFelix NchuRosa SuadesFelix PlamperRosa TundisFelix PlasserRosa VitielloFelix ZulhendriRosalin MishraFeng GuoRosalva Mora-EscobedoFeng JianfangRosalynn NankyaFeng QiuRosamaria CapuanoFeng XuRosamaria PennisiFeng YinRosângela Da Silva LaurentizFengchao CuiRosanna Di PaolaFenghai GuoRosanna MaccariFenghua ZhangRosaria AcquavivaFenglei YangRosaria Anna PiccaFenglin HuRosaria BrunoFenglin ZhangRosaria CostaFengping YiRosaria SchettiniFengqi GuoRosário AnjosFengqing YangRosario DonatoFengzhi LiRosario OlivaFerdinando CostantinoRosemar AntoniassiFerdinando FebbraioRoshan KumarFerenc BudanRoshan M. BorkarFerenc GallyasRoshan PrabhakarFerenc JoóRosilda Mara MussuryFerenc OroszRosita DianaFerhan TümerRositsa ShumkovaFerhunde AysinRoslan IsmailFerman ChavezRoslyn BathgateFernanda DiasRoss C. LarueFernanda GomesRossana MorabitoFernanda PeyronelRossella De MarcoFernanda RaffinRostislav BukasovFernanda VilarinhoRoto RotoFernando C. BaltanásRouba NasreddineFernando CalzadaRoumiana P. StatevaFernando CardonaRoussin Lontio FomekongFernando CardosoRoxana FolescuFernando Cordeiro RaposoRoxana GhiulaiFernando CortésRoxana LucaciuFernando EcheverriRoxana MedinaFernando García TelladoRoxana Mitzayé Del CastilloFernando J. PereiraRoxana RacoviceanuFernando Lopitz OtsoaRoxana-Maria AmărandiFernando Martínez-MoralesRoya R. R. SardariFernando MonroyRoya ZandiFernando PablosRozangela Curi PedrosaFernando Padovan-NetoRozina KhattakFernando RamosRu HeFernando Rivera-LópezRu JiFerran Acuña-ParésRuangdaj TongsriFerreira Nelson FerreiraRubem Mário Figueiró VargasFerruccio PetrucciRubén AgregánFestus Adeyemi AdejoroRubén CaballeroFevzi BardakciRubén Francisco González-LaredoFezah OthmanRubén González-NúñezFilbert TotsinganRubén López-FonsecaFilip AndrićRuben Soto-AcostaFilip DujmićRuchika BhawalFilip ŠupljikaRuchira ChatterjeeFilipa A. VicenteRuchira NandasiriFilipa MandimRüdiger HellFilipa S. ReisRudy LuckFilipe BuarqueRuengwit SawangkeawFilipe Hobi Bordón SosaRui A. LimaFilipe PenteadoRui CostaFilipe Pereira-DutraRui HouFilipe PintoRui HuangFilipiak Marcin SzymonRui LiuFilippo BasagniRui M. B. CarrilhoFilippo BiamonteRui M. S. CruzFilippo De CurtisRui OliveiraFilippo FratiniRui YanFilippo M. SantorelliRui YangFilippo PernaRui YuFilippo RossiRuihao LiFilomena NazzaroRuihua DongFiona LimanaqiRuijie D. ZhangFiore Pasquale NicolettaRuijie MaFiorenzo Piccioli CappelliRuijing LiangFioretta AsaroRuilin LiuFirdos Alam KhanRuilong ShengFiroz AkhterRuimao HuaFirzan NainuRuiqing FanFitria RahmawatiRuisheng GuoFlavia Carla MeottiRuiwu LiuFlávia SousaRuixia GaoFlavia ZacconiRuixia LiuFlavio RizzolioRuixue SunFlavio TidonaRuiyang BaoFleming Martinez RodriguezRuizhi HanFlorence Djedaïni-PilardRukang ZhangFlorence McCarthyRukiya MatsidikFlorenci V. GonzálezRumana RanaFlorencio SantosRumiana TzonevaFlorent BarbaultRune SlimestadFlorent CalvoRuojun WangFlorentina GateaRuoyu WangFlorentina Laura ChiriacRupanjali PrasadFlorentina Monica RadulyRupesh DeshmukhFlorentina-Daniela MunteanuRuslan DmitrievFlorian GambusRuslan E. AsfinFlorian GembardtRuss HilleFlorian H. H. BrillRuta MucenieceFloriana D’AngeliRutaiwan TohtongFlorin CiolacuRute Gonçalves MatosFlorin OanceaRuth A. SchwalbeFlorina BranzoiRuth Dudek-WicherFlorina DumitruRuvanthi N. KularatneFlorina M. BojinRuwei LinFlorine CavelierRuxandra StefanescuFnu SurakshaRyan CoppageFohad HusainRyan SengerFoivos PerakisRyo YazakiFong-Fu HsuRyosuke SaijoFong-Yu ChengRyota InokuchiFortunato B. SevillaRyszard AmarowiczFotis BaltoumasRyszard JakubasFotouh R. MansourRyszard RafałowskiFrabrizio OlivitoRyszard RezlerFrady GouanyRyu OkumuraFranca Maria FlorisRyuichiro SuzukiFrancesc PuiggròsRyuta IshikawaFrancesc TeixidorS. Balakrishna PaiFrancesc Xavier AvilésS. G. Eswara ReddyFrancesca AccioniSaad El-Din HassanFrancesca AielloSaad ShaabanFrancesca Alessandra AmbrosioSaadi SamadiFrancesca AnnunziataSaba GillFrancesca ArfusoSabina GaliniakFrancesca BianchiniSabina Lachowicz-WiśniewskaFrancesca BonominiSabina LicenFrancesca BotSabina PodlewskaFrancesca CianiSabine BothaFrancesca DegolaSabine GröschFrancesca FerrarisSabine Sator-KatzenschlagerFrancesca FontanaSabine WindhorstFrancesca GallivanoneSabino PachecoFrancesca GhirgaSabira SultanaFrancesca IemmaSabiruddin MirzaFrancesca IommelliSabna KottaFrancesca LeonelliSabrin IbrahimFrancesca MaffeiSabrina BossioFrancesca ManciantiSabrina DallavalleFrancesca MartuzziSabrina Gensberger-ReiglFrancesca OlivieroSabrina PacorFrancesca RiganoSabrina PalantiFrancesca SciandraSabyasachi ChatterjeeFrancesca SistoSachchida Nand RaiFrancesca UbertiSachiko KoyamaFrancesca VasileSachiko Toma-FukaiFrancesco ArmettaSachin Kumar SinghFrancesco BalestriSachio AsaokaFrancesco BiancoSadaf GilaniFrancesco BimboSadaf JahanFrancesco CacciolaSadafara A. PillaiFrancesco CaddeoSadanand PandeyFrancesco CaponioSadao AdachiFrancesco CaridiSaddam SaqibFrancesco CreaSaeed MozaffariFrancesco D. LofaroSaeed Saber-SamandariFrancesco De NuccioSaeid JafariFrancesco De RosaSafaa KaderFrancesco Della MonicaSafeer AhmedFrancesco Di GennaroSagar PardeshiFrancesco FrecenteSagar RoyFrancesco GaleottiSagar SouravFrancesco LambertiSagarika BiswasFrancesco LopezSagheer OnaiziFrancesco MaioneSahar AhmedFrancesco Maria RaimondoSai Reddy DodaFrancesco MeneguzzoSai Sathish RamamurthyFrancesco OrtusoSaid GharbyFrancesco P. BusardóSaid I. BehiryFrancesco Paolo FanizziSaida OrtolanoFrancesco PiacenteSaied SolimanFrancesco RuffoSaikat DuttaFrancesco Saverio Robustelli Della CunaSaikat Kumar JanaFrancesco SessaSaikat MitraFrancesco SianoSaima ImranFrancesco TaddeoSaiyang ZhangFrancesco TavantiSajad Ahmad SofiFrancesco TornabeneSajad Majeed ZargarFrancesco ZerbettoSajad WaniFrancis BougieSajal Kumar DasFrancisc BodaSajal SenFrancisca Pereira De MoraesSajal ShrivastavaFrancisca RodriguesSajesh ThomasFrancisca VicenteSajid AliFrancisco AlvesSajjad Hussain SumrraFrancisco ArriagadaSajjad U. AhmadFrancisco B. C. MachadoSajjad UllahFrancisco Bolás-FernándezSaju JosephFrancisco Cortés-BenítezSakander HayatFrancisco Cruz-SosaSaket PatelFrancisco Fabio Oliveira De SousaSaketh ChemuruFrancisco FoubeloSakineh AbbasiFrancisco Gabriel Vázquez-CuevasSakineh Kazemi NoureiniFrancisco Gracia-CanovasSalah Raza SaeedFrancisco Humberto Xavier-JuniorSalah S. MassoudFrancisco J. Aguirre-CrespoSalam IbrahimFrancisco J. BlancoSałdyka MagdalenaFrancisco J. Fernández-ÁlvarezSalem ElkahouiFrancisco J. R. MejíasSalihu IbrahimFrancisco J. Salafranca LázaroSalim AlbukhatyFrancisco Javier Alarcon-AguilarSalim MorammaziFrancisco Javier Leyva-JimenezSally FreemanFrancisco Javier López-TenlladoSalmabanu LuharFrancisco Javier LuqueSalman A. KhanFrancisco Javier Otero-EspinarSalman Afroze Azmi MohammedFrancisco José Fernández-GómezSaloua BiyadaFrancisco José González-MineroSalud Pérez-GutiérrezFrancisco José Tôrres De AquinoSalvador E. Maestre PérezFrancisco LemosSalvatore AdinolfiFrancisco LeónSalvatore Antonino RaccuiaFrancisco LesSalvatore BaldinoFrancisco Lopez-LinaresSalvatore BarrecaFrancisco MeijideSalvatore ChirumboloFrancisco MendezSalvatore DanieleFrancisco MonteSalvatore GrassoFrancisco MouraSalvatore GuarinoFrancisco SautuaSalvio Suárez-GarcíaFranck CosteSam Al-DalaliFranck RataboulSam HayFranco CervellatiSam PeringFranco FurlaniSaman GhahriFranco MutinelliSamanta RaboniFrançois CoudercSamantha E. WilnerFrancois DehezSamantha MicciullaFrançois DelattreSamarjit KarFrancois GanachaudSamarpan MajumderFrançois NoelSambantham MuthuFrancois RaultSambit MishraFrançois RopitalSameer JoshiFrançois Xavier PerrinSameh A. AhmedFrancoise M. Amombo NoaSameh A. KormaFrank FronczekSameh ElhadyFrank H. QuinaSamer GozemFrank KrumeichSamer HaidarFrank StahlSamet ÖzdemirFranko BurculSamet PoyrazFrantišek KačíkSami AkbulutFrantišek KrčmaSami I. AliFrantisek TrejtnarSami Ullah KhanFranz BracherSami Ullah RatherFranziska HoffmannSamia Aci-SècheFranz-Josef Meyer-AlmesSamia ElseginyFrédéric CastetSamik NandaFrédéric DumurSamir AhmadFrederic HarbSamir ChtitaFrederic PaulSamir El HankariFrederick A. LuzzioSamir F. De A. CavalcanteFrederick C. FelkerSamir H. BarghoutFrederick SarpongSamir Kumar BandopadhyayFrederico PontesSamir Kumar SarkarFrederik DenormeSamira MalekmohammadiFrédérique DucroquetSamiullah KhanFredric M. MengerSamo SmrkeFredrik BjorklingSampa GuptaFredy A. SilvaSampa SahaFredyc DíazSamson KheneFreeborn RwereSamuel DagorneFreija De VleeschouwerSamuel Fernández-ToméFu Wa LeeSamuel GarciaFufeng LiuSamuel GuieuFuhang SongSamuel K. KwofieFuhua HaoSamuel M. SilvestreFui YongSamuel PitaFuji JianSamuele MaramaiFujun ZhangSamuil UmanskyFulvio CiriacoSamy Mahmoud ElmegharbelFuming TsaiSan Ming WangFumitaka HayashiSana AshrafFumiyuki KiuchiSanaa BerdaweelFunda Karbancioglu-GulerSanaz AlamdariFung-Fuh WongSanbo QinFupin LiuSánchez-Nieves JavierFuquan YangSanchita KunduFurong TianSanda AndreiFushuang LiSandeep KaushalFuyu ChenSandeep Kumar Reddy AdenaGabin GbabodeSandeep Kumar SinghGabin Thierry Mbahbou BitchagnoSandeep MalampatiGabirla KratosovaSandeep SankpalGabor DogossySandesh PanthiGábor KatonaSandhya MishraGábor KozmaSandile Phinda SongcaGábor PethőSandip M. VibhuteGábor TuruSandipan DattaGábor VasváriSándor BenyheGabriel BernardoSándor BeszédesGabriel Del RioSándor GóbiGabriel EstrelaSandor HosztafiGabriel HancuSándor KékiGabriel IwonaSándor ValkaiGabriel MerinoSandra BarbalhoGabriel MoncaliánSandra CasimiroGabriel Navarrete-VazquezSandra CastilloGabriel PlavanSandra D. AdamsGabriel Ramos-OrtizSandra DavernGabriel VasilieviciSandra Flinčec GrgacGabriel YbarraSandra GemmaGabriela Aguilar-TipacamúSandra HilárioGabriela BuemaSandra M. Martin GuerreroGabriela FeresinSandra MendozaGabriela GuillenaSandra Oramas-RoyoGabriela HrckovaSandra OsésGabriela IonitaSandra PinaGabriela Medina-PérezSandra RebeloGabriela Olimpia IsopencuSandra RistoriGabriela Pastuch-GawolekSandra SimõesGabriela RosaSandra Vezmar KovačevićGabriele CarulloSandrine BouquillonGabriele Loris BeccaroSandrine ChiffletGabriele MancaSandrine CojeanGabriele RocchettiSandrine DourdainGabriella SavianoSandrine LorimierGabrijela Tavcar KalcherSandro José GrecoGaëtan L. A. MislinSandro Jose Ribeiro BonattoGaetano AngeliciSandro NavickieneGaetano MarvertiSang Gil LeeGaetano PandinoSang Moon KimGaetano ZuccaroSangbum ParkGagan FloraSanggil HanGagandeep Kaur GahlaySang-Han LeeGagandeep SagguSanghee LeeGaganpreet KaurSang-Hyup LeeGajanan GhodakeSangita GangulyGalbada Arachchige Sirimal PremakumaraSangkyu LeeGalina A. KovalenkoSang-Nam KimGalina LikhatskayaSangwon ChaGalina NikolovaSangwoo LeeGalip AkaySanja BabićGalya TonchevaSanja BosnarGamal MohamedSanja GlisicGanesh C. NikaljeSanja J. ArmakovicGanesh KoyyadaSanja Kostadinović VeličkovskaGanesh KumarSanjay GuptaGanesh MurhadeSanjay KumarGanesh SableSanjay PatelGanesh YadaigiriSanjay SainiGaneshlenin KandasamySanjay SinghGang DingSanju GuptaGang Seob JungSankar SekarGang WeiSankarprasad BhuniyaGangadhar AndaluriSanket MishraGangarapu KiranSanna ManuelaGangli ZhuSantanu GhoshGanglong CuiSantanu PanjaGao LiSanthana Krishna Kumar AlagarsamyGao-Yi TanSanti MandalGarbine AguirreSantiago CasadoGareth MorganSantiago Diaz-OltraGareth R. EatonSantiago Garcia-GrandaGarry OsthoffSantiago Garcia-VallvéGartzen LopezSantiago MadrugaGaspard HuberSantiago P. AubourgGaulthier RydzekSantiago ReinosoGaurav GoelSantiago RodríguezGaurav SharmaSantiago UrrejolaGautam SethiSantina ChiechioGavino SannaSantosh A. MisalGaweł SołowskiSantosh BashyalGayong ShimSantosh Kr. KarnGbemisola FadimuSantosh Kumar SinghGeciane Toniazo BackesSapna LangyanGediminas NiauraSaqib AliGeert HaarlemmerSaqib HassanGeeta Devi LeishangthemSara Alonso-TorreGeetha BollaSara BaldassariGellert KarvalySara CrucianiGelson PerinSara Della TorreGemilang L. UtamaSara HedayatiGemma TriolaSara KhanGencay SariisikSara La MannaGengjun ChenSara Morales RodrigoGeni Rodrigues SampaioSara NocentiniGennadii Georgievich MikhailovSara PalmieriGennady EvtugynSara RealistaGennady M. PoletaevSara Sáez-OrvizGennady SemisotnovSara SattinGeorg VoelckerSara SaviniGeorge A. MousdisSara SilvaGeorge AngelovSara Tariq A. Al-RashoudGeorge C. StewartSarah Cirilo GimenesGeorge FleetSarah J. PurdyGeorge J. StathasSarah LaiGeorge KenanakisSarah MazzottaGeorge LambrinidisSarana SommanoGeorge MagoulasSaranya PoapolathepGeorge TsogkasSaravana KumarGeorge TzotzosSaravanan MuthupandianGeorge TzvetkovSarfaraz Hashemkhani ZolfaniGeorge VarvounisSarfaraz HussainGeorge W. GokelSarfraz ShafiqGeorge ZachariadisSarita ForsbackGeorges MaestroniSariya MapoungGeorges MassiotŠárka PaušováGeorgi Atanasov KostovŠárka ŠtěpánkováGeorgi BeevSarkar M. Abe KawsarGeorgia-Eirini DeligiannidouSarkyt KudaibergenovGeorgiana CodinaSarmento MazivilaGeorgiana NitulescuSarmistha TalukdarGeorgieva KatyaSaroat RawdkuenGeorgios P. MastrotheodorosSarojini ShankarGeorgios PampalakisSarpietro Maria GraziaGeorgios PapadopoulosSartaj Ahmad BhatGeorgios TsaniklidisSaša ĐurovićGeorgios VogiatzisSaša FrankGeorgiy GamovSaša KazazićGeovanna Quiñonez-BastidasSaša PrđunGera MeetaSascha RohnGéraldine GouhierSatheesh ChandranGerard DumancasSathish DyawanapellyGerard Lee L. SeeSathiyaseelan AnbazhaganGerardo BykSatish Chander BhatlaGerardo Casanola-MartinSatish GandhesiriGerardo Cebrián-TorrejónSatomi NiwayamaGerardo CorzoSatoru TamuraGerardo Díaz-GodínezSatoshi KomatsuGerardo Fernández BarberoSatoshi YamauchiGerardo Leyva-GómezSatya Murthy TadinadaGerardo Santos-LópezSatyendra KumarGerardo Vázquez-MarrufoSatyendra MishraGerardo ZepedaSaud A. AlfayezGerasim Vladimirovich KrivovichevSaúl Gómez-ManzoGerd KnuppSaurabh DubeyGergana MihailovaSaurabh GautamGergely FarkasSaurabh JhaGergő TóthSaurav Kumar JhaGerhard GröbnerSavini SaraGerhard KönigSaviour A. UmorenGerry LushingtonSayandeep GhoshGerui RenSayed Haidar Abbas RazaGevany Paulino De PinhoScott A. JarmuchGeza BujdosoSean ChiaGhada BouzSean L. StokesGhada HadadSean O’KeefeGhadha Ibrahim FouadSebastiaan WertenGhareb SolimanSebastian BonarddGhasem AlaviSebastian EhrlingGheorge IliaSebastian GranicaGheorghe BorodiSebastian PomplunGheorghiţa MitranSebastian TancoGhislain Wabo FotsoSebastiano CampisiGhizlane EnaimeSebastiano GarroniGhodrat MahmoudiSebastião PratavieiraGholamhossein SodeifianSebastien AlphonseGholamreza DaryaborSebastien Z. CausseGhulam Mustafa KamalSeda KoyuncuGi Ok KimSee Mun LeeGiaan Arturo Álvarez-RomeroSefater GbashiGiacomo LuciSegula MasaphyGiacomo MusileSehrish MananGiampietro ViolaSeiichi MatsugoGian Paolo BarzantiSeiji MiyauchiGiancarlo AldiniSe-Jae KimGiancarlo CravottoSejoon LeeGiancarlo PascaliSekar VijayakumarGiancarlo RanalliSekelwa CosaGianella FacchinSelma BeyziGianfranco FaviSelmin FrancescaGianina DodiSemen N. KlyamkinGianluca BaldanziSenay UstunelGianluca CatucciŞenay YurdakulGianluca OttolinaSengul Alpay-KaraogluGianluca TripodiSenka PopovićGianluigi AlbanoSenthil NagappanGianluigi De GennaroSeok-Ho KimGianluigi LauroSeong Beom ChoGiannantonio BattistuzziSeong Jun ParkGianpaolo PapaccioSeongkyu YoonGianpaolo ZerbiniSepalika Swarnamali Baththana MudiyanselageGianpiero GarauSerban C. MoldoveanuGiau VoSerena ArnaboldiGigliola LusvardiSerena De SantisGihan KamelSerena OrlandiniGijs A. KleterSerena ReggiGil FraquezaSerge Alain Fobofou TanemossuGil SalvadorSerge BottariGilbert BellangerSergei A. MoshkovskiiGilberto Dantas SaraivaSergei Andreevich DzubaGilberto Velázquez-JuárezSergei BronnikovGilles MarcouSergei N. PiskunovGina MendezSergei VatsadzeGintarė KručaitėSergej PirkmajerGioele PagotSergejs GaidukovsGiorgia BrancoliniSergey A. AdoninGiorgia ContaSergey A. DenisovGiorgia MoschettiSergey A. KarpushenkovGiorgia NovelloSergey BaykovGiorgio CapuaniSergey BelyakovGiorgio FacchettiSergey DyshlovoyGiorgio OlivoSergey G. ZlotinGiorgio RicciSergey GudkovGiorgio SmaldoneSergey KichanovGiorgio SoldaniSergey LarinGiorgio TofaniSergey M. IvanovGiovana JamarSergey MakarenkoGiovani Carlo Veríssimo Da CostaSergey N. KonchenkoGiovanna Barbarini LongatoSergey PasechnikGiovanna BaronSergey TikhonenkoGiovanna EspositoSergey TimofeevGiovanna LonghiSergey V. KolotilovGiovanna RigilloSergey V. SemikolenovGiovanna RomanoSergey VatsadzeGiovanna Rossi-MarquezSergey VereshchaginGiovanna SimonettiSergi MaicasGiovanni BartolomeoSergio A. BucareyGiovanni BatignaniSergio AbbateGiovanni Battista Di PierroSergio Antonio Marques LimaGiovanni Battista GiovenzanaSergio E. RodriguezGiovanni BertoldiSérgio F. SousaGiovanni BottariSergio FerlitoGiovanni CaminoSergio GalembeckGiovanni CavalloSergio Galindo RodriguezGiovanni ChiappettaSergio GiuffridaGiovanni Freitas GomesSergio Gómez-CornelioGiovanni GhigoSergio M. AcuñaGiovanni GrannucciSergio MartíGiovanni GraziosoSergio OrtizGiovanni GriecoSérgio P. C. AlvesGiovanni La PennaSergio Pérez-BurilloGiovanni MarzaroSergio RossiGiovanni MessinaSergio Ruffo RobertoGiovanni Nicola RovielloSérgio SilvaGiovanni RaugeiSergiy Mykolayovych KovalenkoGiovanni RibaudoSergiy V. RosokhaGiovanni RicciSerhat Sezai ÇiçekGiovanni RollaSerhii VakalGiovanni RovielloSerkan Serkan SelliGiovanni SansoeSerkos A. HaroutounianGiovanni TafuroSerrano SaludGirish KumarSetshaba D. KhanyeGirish PattappaSeung Ho LeeGirolamo CasellaSeung Myung MoonGiulia AbateSeunghwan BaeGiulia BivonaSeverine BoyerGiulia DowgierSeverino Matias De AlencaraGiulia FioravantiSevgi PolatGiulia LunghiSevil Savaşkan YilmazGiulia MarroneSevim AkyüzGiulia MoroSevki CesmeciGiulia RussoŞeyda BostancıGiulia SelvoliniSeydi YıkmışGiulia TuciSeyed Ali SajadianGiulia VantiSeyed Fakhreddin HosseiniGiuliana CangemiSeyed Khosrow TayebatiGiuliano RamadoriSeyed Mohammad MazloomiGiulio PoliSeyyed Alireza HashemiGiulio VernaSeyyed Mehdi KhoshfetratGiulio VistoliSeyyed Mojtaba MousaviGiuseppa Di BellaSezer OkayGiuseppa GraceffaShaaban Abd El AalGiuseppe ArenaShadab MdGiuseppe CassoneShadeed GadGiuseppe CirilloShadia A. ElsayedGiuseppe De VitoShadma W. AbdulwahabGiuseppe ErmondiShadma WahabGiuseppe FerrandinoShafi MahmudGiuseppe FlorestaShafiq Ur RahmanGiuseppe GrazianoShafiq Ur RehmanGiuseppe LazzaraShafiqur RahmanGiuseppe MancusoShagufta PerveenGiuseppe ManninoShah RukhGiuseppe MarcìShahenda MahgoubGiuseppe MartelliShahid AdeelGiuseppe MartinoShahid BashirGiuseppe MazzeoShahid KhanGiuseppe MicalizziShahid M. IqbalGiuseppe MuscasShahram MahmoudiGiuseppe OrsomandoShahriar KhanGiuseppe PalmieriShahzad IqbalGiuseppe PappalardoShahzad SarwarGiuseppe PellicaneShahzad Zafar IqbalGiuseppe QueroShahzada Qamar HussainGiuseppe SancesarioShaida Fariza SulaimanGiuseppe SimoneShaik Gouse PeeraGiuseppe SuariaShailendra S. GuravGiuseppe Trusso SfrazzettoShakeel Ahmad KhandayGiuseppe ZagottoShakhawan AlzanganaGiuseppe ZanottiShalini S. AryaGiuseppina Anna CorrenteShamil LatypovGiuseppina LucianiShamir CassimGiuseppina Pinuccia CerratoShams TabrezGiuseppina SabatinoShangfeng DuGiustino OrlandoShan-Hui HsuGizem Gulsoy ToplanShanmuga S. MahalingamGleb TselikovShanmugam HemaiswaryaGleison Antônio CasagrandeShanna Bastiaan-NetGleiston DiasShan-Shue WangGloria CastellanoShan-Ying WuGloria Huerta-AngelesShaofei JiGloria ManzottiShaohua ChenGloria MazzoneShaohua JiangGloria MoleroShaoping LuGloria NombelaShaowei ChenGloria Uccello-BarrettaShaowei WangGloria Yepiz-PlascenciaShao-Wen HungGodvin Sharmila VincentShaoyong LuGohar KhachatryanSharafaldin Al-MusawiGökçe Şeker KaratoprakSharanabasava GanachariGökhan AcikSharanabasava V. GanachariGomaa Abdelgawad Mohammed AliShariful IslamGomaa SanadSharon HilarydossGon Sup KimSharon J. Nieter BurgmayerGoncagül SerdaroğluSha-Sha ChengGonçalo CarreraShashank PathakGonçalo J. JustinoShashanka RajendrachariGonçalo TiagoShashi Kant ShuklaGongli TangShashi Shekhar SinghGonzález-Veloso IvánShaukat AliGonzalo IzaguirreShawn ChristensenGonzalo Martínez BarreraShawna L. McMillinGonzalo Ortiz De Elguea-CulebrasShayon BhattacharyaGonzalo ScaleseShaza Al-MassaraniGopakumar GopalakrishnanShazid Md. SharkerGopal Lal KhatikShehzad HussainGopal PatelSheikh Ahmad Izaddin Sheikh Mohd GhazaliGopal SinghSheikh MansoorGopalsamy Rajiv GandhiSheng FangGopichand GuttiSheng GuoGopinath KrishnasamySheng LiangGopinath VenkatramanSheng YinGorakhnath JachakSheng ZhangGoran BaranovićShengbao CaiGoran GajskiShengbo GeGoran MitulovicShengcai ZhengGordana ĆetkovićSheng-Da ZhangGordana GajicSheng-Heng ChungGordana MendašShengli ZhaiGordon W. IrvineSheng-Nan ChangGorica IvanišSheng-Nan WuGórnicka MagdalenaShengxing MaGosteva AlevtinaShengyu DaiGottfried J. PalmShensheng ChenGouse Peera ShaikSher Singh MeenaGoutam MukherjeeShereen MowakaGovind PathakSherif MoussaGovindan Suresh KumarSherif T. S. HassanGovindasamy ManiShian-Ren LinGovindasamy RajakumarShiao-Wei KuoGowhar AliShiba DandpatGozde DumanShibdas BanerjeeGrace PatlewiczShichao DingGracia Merino PeláezShichao LvGraciela MahlerShifa WangGraciela MoralesShifeng ChuGraham N. NewtonShigeki KuwataGraham SaundersShigeki SetoGrant CampbellShigemi SeoGrant HillShigenori KumazawaGrazia CottoneShigenori TanakaGraziella MalandrinoShigeyuki YamadaGrazielle Náthia-NevesShih-Chieh HsuGrażyna LewandowiczShih-Hsun HungGrażyna WójcickaShih-Min HsiaGregorio Hernández CocoletziShih-Yen LoGregorio PeronShikui TuGregory A. BardingShilin CheungGregory CaputoShilong ZhengGregory DudleyShiming LiGregory S. YablonskyShiming PengGregory SmithShimon Ben-ShabatGreta AdamczykShin EnosawaGreta FaccioShin KimGreta VolpedoShin YasudaGrigory V. ZyryanovShin-Hun JuangGrzegorz AdamczukShini FengGrzegorz BartoszShin-ichi KayanoGrzegorz ChladekShin-Ichi MiyatakeGrzegorz CholewinskiShinichiro OgawaGrzegorz ChrzanowskiShining LooGrzegorz CieslarShinji OhtaGrzegorz GladyszewskiShinobu SatoGrzegorz GrynkiewiczShintaro KawamuraGrzegorz JozefaciukShintaro KodamaGrzegorz KłosowskiShinya HasegawaGrzegorz KowalskiShinya MatsuzakiGrzegorz LesnierowskiShinya TakahashiGrzegorz LitwinienkoShiqiang GongGrzegorz Nałęcz-JaweckiShiro HikichiGrzegorz SchroederShiro KoizumeGuadalupe García-AlcocerShiru LinGuangci LiShisong RenGuangde JiangShisuo FanGuangfu WuShiuh-Tzung LiuGuangjin HouShiva GuduruGuangle NiuShiva MohajerniaGuanglei WuShivananda KandagallaGuangming LiShivani DixitGuangqiang XuShivankar AgrawalGuangrong MengShivatharsiny RasalingamGuangyi MaShivkanya FuloriaGuangyu ZhuShi-Xia LiuGuangzhi ShanShiyuan LiuGuan-Jhong HuangSho TsukamotoGuanjie HeShofarul WustoniGuanwu WangShogo NobukawaGudrun E. KoehlShoji MatsumotoGudrun KoehlShoji OdaGuedmiller OliveiraShoji TakenakaGuendalina ZuccariShokouh HaddadiGugan KothandanSholpan AskarovaGuido J. ReissShoshana AradGuido PanzarasaShota FujiiGuiguang ChengShoufeng TangGui-Guang ChengShoukui HeGuilhem ArrachartShowkat DarGuilherme Lanzi SassakiShozo TomonagaGuillaume BouvierShravan MorlaGuillaume J. BilodeauShreesh K. OjhaGuillaume NonglatonShreyans Kumar JainGuillaume StirnemannShreyas KuddannayaGuillem Campmajo´Shrikanth GadadGuillermo AhumadaShu XuGuillermo CásedasShuai MaoGuillermo Cristian Guadalupe Martínez ÁvilaShuai RenGuillermo Díaz-SainzShuai WangGuillermo Moreno-AlcantarShuai ZhangGuillermo PetzoldShuaihang PanGuillermo TejadaShuang LiuGuillermo Tellez-IsaiasShuang SongGuining LuShuangjiang LuoGuiping RenShuangqiang ChenGuishan LiuShuangyang ZouGuiyang HaoShubai LiuGuizhao LiangShubha RaoGul RahmanShubhangi ShuklaGulamnabi L. VantiShubhankar BhattacharyyaGulay OzkanShubhankar Kumar BoseGulcin YildizShugeng CaoGuliz ArmaganShuhei YamadaGulsu SimsekShu-Hui ChenGulyaim N. SagandykovaShuiquan DengGunendra Prasad OjhaShujaat AhmadGunnar AntoniShuji AkaiGunter KuhnleShujian ZhengGünter MüllerShujie YangGünter SchwarzShujing LiuGuobin XueShujuan YuGuocai TianShujun DongGuocheng HuangShujun LiGuohua MaShuling HsiehGuohua XieShunbo LiGuohui LiShunichiro ItoGuojin LiangShunji ItoGuoli TuShunji NatsukaGuo-Liang LuShunsuke OhtaniGuoqing WangShunsuke YajimaGuor-Jien WeiShuntaro TakahashiGuosheng ChenShuntaro TsubakiGuosheng LiShun-Xing LiangGuowei ShuShuo BaiGuowen MengShuo ShiGuoxu MaShuqing DongGuoyuan ZhuShusaku AsanoGurdeep MarwarhaShuvadeep MaityGurpreet SinghShuxun LiuGuru KarthikeyanShu-Yao TsaiGuru Raghavendra ValicherlaShu-Yen LinGurudeeban SelvarajShuzhen JiangGurunathan JayaramanShveta BathlaGururaj Kudur JayaprakashShwet KamalGustavo A. González-ÁguilarShyam Kishor SahGustavo Alberto ChiabrandoShyamalava MazumdarGustavo Cabrera-BarjasShyh-Jiun LiuGustavo Desire Antunes GastalShyi-Long LeeGustavo Gonzalez-NevesShyi-Tien ChenGustavo Moreno-MartínSi LiGustavo SalinasSian DuttonGutru RambabuSıbel YağcıGuy G. PoirierSibhghatulla ShaikhGuy JacobSichi LiGuy MechrezSiddabasave Gowda B. GowdaGuy SchmerberSiddesh V. HartimathGuy ZuberSiddharth GuptaGwenaelle Lavison-BompardSiddharth MatikondaGwon Woo ParkSiddharth PandeyGynendra Kumar RaiSiddhartha PatiGyöngyi GyémántSiddheshwar Kisan ChautheGyörgy BaloghSidharth MehanGyörgy KeglevichSidi ZhuGyorgy SzalokiSidney BeckerGyörgy Tibor BaloghSidney CreutzGyörgy TrencsényiSidra AslamGyörgyi VáradiSiemowit MuszyńskiGyo-Woo LeeSijin WuGyudo LeeSikandar I. MullaGyula BattaSikandar KhanGyula HalasiSilvana AlborésGyungsoon ParkSilvana AlfeiH. N. Aswatha RamSilvana CasatiH. Q. Nimal GunaratneSilvana PedatellaHa Bich TrinhSilvano GeremiaHa RadhaSilvano Junior SantiniHa Vinh Lam NguyenSilvestre Bongiovanni AbelHabibullah KhalilullahSilvia AngelovaHadi HadshemzadehSilvia BordoniHafiz M. N. IqbalSilvia BorsacchiHafiz Muhammad Abd Ur RahmanSilvia CarlottoHafiz Muhammad Salman AjmalSilvia CarvalhoHafiz Muzzammel RehmanSilvia ChichiarelliHafiz-Muhammad-Umer FarooqiSilvia DeiHaggeo DesirenaSilvia FischerHai DongSilvia Laura Guzmán-GutiérrezHaibing ZhouSilvia Marquina-BahenaHaibo YuSilvia MarracciHaibo ZhaoSilvia MironeasaHaibo ZhouSilvia MottaHaichuan WangSilvia Ortega-GutierrezHaider RazaSilvia P. Tsanova-SavovaHaidong LiSilvia RinaldiHaifeng DuSilvia RizzatoHaifeng HeSílvia SimonHaifeng SunSilvia Simon RabassedaHaigang WuSilvia VasiliuHaihui Joy JiangSilvia ZorrillaHailan ChenSilvina CervenyHaiLi LiuSimion KreimerHailong LiSimon DuvalHainan SunSimon HinkleyHaipeng ZhangSimon PfistererHaiqing ZhaoSimon SieberHairul AbralSimon StephanHairul Islam Mohamed IbrahimSimon StrobbeHaisheng HeSimona BroncoHaishu LinSimona CavaluHaisu ZengSimona CeccarelliHaitao WuSimona ConcilioHaitian FangSimona CrognaleHaiyan HuSimona De De VitaHaiyan PeiSimona FabroniHaiyan TanSimona FellettiHaiyang WangSimona GavrilasHaizhen Andrew ZhongSimona MartinottiHaizhou ZhuSimona NoveanuHajime KojimaSimona OanceaHajra ZafarSimona PergolizziHak Jun KimSimona SagonaHakan ArslanSimona ViolinoHala Mohamed El HefnawySimone CantamessaHalil I. CiftciSimone CarradoriHalina JurkowskaSimone CavaleraHalina KrawiecSimone KönigHamdoon MohammedSimone Monteiro SilvaHamdy Abdel-Gawaad ShaabanSimone RonsisvalleHamdy F. M. MohamedSimone SabbionedaHamed I. AliSimone SavinoHamed NosratiSimone ZorzanHamid Bakhsheshi-RadSina NaserianHamid OsmanSina Shaffiee HaghshenasHamidreza BagheriSingh RajenderHamilton B. NapolitanoSinha IndrajitHamilton CabralSinosh SkariyachanHamish S. SutherlandSin-Yeang TeowHammad UllahSio-Hong LamHamza MechchateSipho MdandaHamzah Abdulrahman SalmanSirajuddin AhmedHamzeh GhorbaniSirajudheen AnwarHan HanSirajul HaqHan RichouSiraprapa BrooksHan ZhangSirinya ChantarakHana UjčíkováSiriporn OkonogiHanae Naceiri MrabtiSirivan AthikomkulchaiHanan AatiSiseth Martínez-CaballeroHanane Boutaj‪Siska AdityaHanbin HuangSitanshu S. SinghHandoo RheeSitesh Chandra BacharHang HuSithisarn PongtipHang QiSiti Aqlima AhmadHangzhen LanSiti BaidurahHani El MollSiti Hamimah Sheikh Abdul KadirHani M. HafezSiti KamalHani N. NaseefSiti Kholijah Abdul MudalipHani Nasser AbdelhamidSitthivut CharoensutthivarakulHan-Jun KimSittinan ChanaratHanki LeeSiva Kumar NadavalaHanmant GaikwadSiva Lahiri Kanth NanduriHanna BandarenkaSiva MurruHanna BaranowskaSiva S. PandaHanna BarrigaSivamani SelvarajuHanna Dams-KozlowskaSivapriya KirubakaranHanna SzaeferSivasankara Rao EdeHanna SzczepanowskaSivashanmugam AmirthalingamHannah PohlitSiwapon SrisonphanHanne DiliënSiwaporn BoonyasuppayakornHanoch SenderowitzSixun ZhengHanquan SuSkorjanc TinaHans A. Eyeghe BickongSlađana ĐurđićHans G. DrexlerSladjana JevremovicHans H. HendriksSladjana KrivokapićHans Heinrich LimbachSladjana ŠobajićHans LeemhuisSlaven JurićHans Van LeeuwenSlaveya PetrovaHans-Christian SiebertSlavica MaleticHanwen WangSlavko RadenkovićHany Abd El-LateefSławomir GonkowskiHany Ezzat KhalilSławomir Józef KrzebietkeHany G. Abd El-GawadSławomir KociraHany H. ArabSławomir MakowiecHany Hamdy ArabSlawomir NeffeHao JiangSławomir SułowiczHao LiSlawomir WybraniecHao MeiSlim SmaouiHao NieSlimane MerouaniHao PengSmaoui SlimHao WangSmarak BandyopadhyayHao YangSnežana Č. JovanovićHao YuSnežana D. ZarićHaofan YangSnezana Ilic-StojanovicHaohua DengSnežana M. MiloševićHaoling SunSnežana Zdravković KoraćHaolong ZhouSnježana P. KazazičHaomin LiuSobhi DanielHaomin WangSobhi M. GomhaHaoming BaoSobia Ahsan HalimHaosheng LinSodio C. N. HsuHao-Wen ChengSofia CanolaHarada HiroyukukiSofia LimaHaralambos E. KaterinopoulosSofia MitakouHaralambos StamatisSofia Moran-RamosHarald ClausSofia ReisHarald KolmarSofia RhizopoulouHarald W. PlattaSofia SoaresHarant HannaSoha AhmadiHarbinder SinghSohailHardeep Singh TuliSohail Anjum ShahzadHardik AminSoheil Abbaspour-RavasjaniHari Datta KhanalSoheil SobhanardakaniHari Krishna NamballaSoichiro NakamuraHari Krishna RajanSoji ShimizuHari Prasad DevkotaSokhee JungHarichandra TagadSolange MagalhaesHarish KumarSoledad MateoHarley Da Silva AlvesSoliman KhatibHarold PhilipsenSolmaz MalekiHarri H. HakovirtaSom DevHarri LönnbergSomasundaram SivachandiranHarshad LadeSomayeh TaghaviHartmut KuhnSomdet SrichairatanakoolHaruaki KitagawaSomdutt MujwarHaruhisa KikuchiSomnath PandeyHaruyasu AsaharaSompop BencharitHasan DemirciSona MelikovaHasan HashemiSonani Ravi RaghavHasan SadeghifarSondavid NandanwarHashem AlsaabSongang PengHasi Rani BaraiSonglin WuHasim KelebekSongsong LiHasmadi MamatSongxue WangHasmah AbdullahSongya ZhangHassaan ElahiSoni DhruvKumarHassan AlbarqiSonia BañuelosHassan DastsoozSonia BruñaHassan EbrahimSonia Di GaetanoHassan MehboobSonia Escandón-RiveraHassan Mubarak IshqiSonia FantoneHassan OuachtakSonia LaneriHassan RasouliSonia Losada BarreiroHassan SeradjSônia N. BáoHassan YousefniaSonia Navas-MartinHatem FouadSónia P. MiguelHatem MajdoubSonia PiacenteHatice Kalkan YildirimSonia ScarfìHatice YıldırımSonia SentellasHaya KornweitzSonia SirisiHaya Lorberboum-GalskiSonia TassoneHayrettin O. GulcanSonia TrombinoHayriye GidikSonja Herres-PawlisHazem ElkadySonja Jakovetić TanaskovićHe GuoSonja M. EhlersHe Nucleus XuSoo Yee LeeHeather Jane WalkerSoon-Jung ParkHeba A. GadSophia BorisevichHeba AhmedSophia ChatziantoniouHeba ElgizawySophia LetsiouHeba M. AboudSophia PavlovaHeba SahyonSophie BombardHebah Al UbeedSophie MompelatHéctor C. GoicoecheaSophie VincentiHéctor CarrascoSoraya PazHéctor García-OrtegaSorel Sagu TchewonpiHector M. Mora-MontesSorin HostiucHector Melendez-OrtizSorin Marius AvramescuHéctor Ricardo López GonzálezSorina DinescuHector TsangSotiris AmillisHedvig BölcskeISoudamani SinghHee-Joon KimSouichi OeHee-Sung ChaeSoukaina HilaliHefei ZhaoSoumalya SinhaHefeng ZhangSoumen GhoshHeike LorenzSoumi MitraHeiko LangeSoumitra GuinHeinrich RoderSoumitra PoulikHeinz C. SchröderSounak SahuHeinz Friedrich SchölerSourav ChatterjeeHeitor DaguerSourav Kr. SahaHelber Enrique Balaguera-LópezSourav MondalHelen AspinallSourav RejHelen OsbornSouren PaulHelena AmaroSouzan Vergkizi-NikolakakiHelena Cristina VasconcelosSowbiya MuneerHelena DanielsonSpandana Rajendra KopalliHelena DominSpiro M. KonstantinovHelena FelgueirasSpiros GardelisHelena NetoSpiros ParamythiotisHelena Otmačić ĆurkovićSpyridon MourtasHelena RitchieSpyridon PetropoulosHelena SilvaSpyros PapageorgiouHélio AlbuquerqueSpyros PerlepesHélio SantosSrabanti GhoshHelle WangensteenSravan Gopalkrishnashetty SreenivasmurthyHemanth NoothalapatiSravani AdusumalliHend Abdel BarSravendra RanaHendrik BrinkSrecko StopicHeng ZhangSreed Sharma KanakkillamHenri Pierre Jacquot De RouvilleSreepoorna UnniHenrik OttossonSrihari KonduriHenrique E. TomaSrihari PabbarajaHenrique FernandesSrikanth Kumar GangamHenry ChermetteSrikanth VuppalaHenryk GrajekSrinath PashikantiHenryk JeleńSrinivas ChamakuriHerbert Júnior DiasSrinivas Niranj ChandrasekaranHerbert Michael HeiseSrinivas Reddy PallerlaHerbert Ryan MariniSrinivas SistlaHeribert ReisSrinivasan KrishnanHerman LutterodtSrinivasrao GanipisettiHerman PotgieterSripada S. V. RamasastryHermann WätzigSriram GanesanHermona SoreqSriram VidyarthiHernando S. SalapareSrivani VeeranarayananHerwig PeterlikSrjit DasHesham HaffezStamatios GiannoukosHeui-Soo KimStamatis BoyatzisHeung Joo YukStanislav A. NikolaevskiiHeveline SilvaStanislav GobecHewa Yaseen AbdullahStanislav GroysmanHicham AbououalidStanislav K. PetrovskiiHicham BenabdelkamelStanislav KafkaHicham HarharStanislav S. TerekhovHicham Mahfoz KotbStanislav SukhikhHidayat HussainStanisław KaliszHideaki FujiiStanisław KowalskiHidehiro UekusaStanisław KrompiecHidekazu HiroakiStanisław MazurHideki HayashiStanislaw OłdziejHideki MasudaStanisław WołowiecHideki NakajimaStanislaw ZajaczkowskiHideki OkamotoStavros PoulopoulosHidemasa HikawaSteeve RousselotHidenori TanakaStefan BergerHideo TsukadaStefan Cord-LandwehrHidetaka TorigoeStefan Cristian VesaHideyuki SatoStefan DüsterhöftHiep Thuan LuŠtefan HardoňHikaru SotomeStefan HeunHilal ZaidStefan JoppHilario VidalStefan KuhnHilda María Hernández-HernándezStefan NaumannHileia SouzaStefan RöderHilton DeethStefan StolteHimanshu GoelStefania ButiniHimanshu KathuriaStefania CometaHimanshu Sekhar JenaStefania ContiHirendra Nath BanerjeeStefania GessiHiroaki TaguchiStefania LamponiHirofumi InoueStefania MagdaHirohito IshigakiStefania MazziniHiroki MatsubaraStefania PorcuHiromi HirataStefania PragliolaHiromichi EgamiStefania SutHiromichi SakaiStefania VitaleHiromu KondoStefania ZanettiHironori YoshinoStefania ZappiaHirosato MonobeStefania-Felicia BarbuceanuHiroshi HirataStefanie GerbigHiroshi ImagawaStefanie KleinHiroshi InomataStefano BellucciHiroshi KikukawaStefano BoschiHiroshi NakagawaStefano D’ErricoHiroshi SasamotoStefano De BenedettiHiroshi TakemoriStefano DugheraHiroto TachikawaStefano FornasaroHiroyasu SakaiStefano GianniHiroyasu YamaguchiStefano MarchesiHiroyuki AonoStefano NisiHiroyuki FurutaStefano ParisottoHiroyuki KataokaStefano PieracciniHiroyuki KoshinoStefano RavaioliHiroyuki YamakoshiStefano SainasHisaaki MiharaStefano SuperchiHisahiro HagiwaraStefano TomassiHisashi DoiStefanos ChaitoglouHisashi MatsudaStefanos LeontopoulosHitendra PatelStefka AtanassovaHitesh BorkarStela DimitrovaHitler LouisStela T. DragomanovaHo Yu Au-YeungStelian Sergiu MaierHoang Quoc AnhStelian VladHohyoun JangStepczynska MagdalenaHojjati-Najafabadi AkbarStephan KabasciHolger RuckdäschelStephan LudwigHolly HughesStephan ReshkinHomero Reyes De La CruzStephan WalchHomira BehbahaniStephan WuestHong Duc PhamStéphane FlamentHong HeStéphane HumbelHong Heng SeeStephanie LhezHong JiangStephanie LongetHong WangStephen A. MorrisHong WuStephen AdamsHong ZhaoStephen B. CarrHongbin LiangStephen C. DreskinHongbing LiuStephen C. HardiesHongcun BaiStephen CanhamHongda CaoStephen ChmelyHong-Duck KimStephen FullerHongfei ZhaoStephen HardingHong-Jhang ChenStephen HillHongjian ZhouStephen InbrajHongjuan LiStephen StanfillHongjun JinSteva LevicHongjun ParkStevan ArmakovićHongliang BaoSteve HolroydHongliang HeSteve ScheinerHonglue ShiSteve ShnyderHonglun WangSteven GoreHongming SuSteven Paul NisticòHongnan CaoSteven Sheng-Shih WangHongping ChenSteven W. BucknerHong-Ting Victor LinSteven WhittenHongwei YangStojanovska VioletaHongwei ZhangStoyan GutzovHongyan LiStrahinja KovacevicHongyi QiStrzemski MaciejHongying ShenStuart G. CollinsHongyu RenStuart SnowdenHongzan SongSu Datt LamHooi-Leng SerSubaer JunaediHoon KimSubashani ManiamHooshyar HossiniSubba Rao CheekatlaHortencia Gabriela Mena-ViolanteSubhabrata MaitiHosam A. SaadSubhajit ChakrabortyHosam O. ElansarySubhamay PramanikHossain Mohammad Arif UllahSubhash Dattatraya TanpureHossam DonyaSubhasis ChattopadhyayHossam El-BeltagiSubhasish SahaHossam M. HassanSubodh DubeyHossein AhangariSubrat Kumar PattanayakHossein BaniasadiSubrata BanikHossein BaraniSubrata JanaHossein EsmaeiliSuchata KirdponpattaraHossein GhafouriSuchithra Ashoka SahadevanHossein Maleki-GhalehSu-Der ChenHossein MolaviSudhir Kumar PaidesettyHossein ZarrinfarSudipta PanjaHouda BenhelliSueli Coelho Da Silva CarneiroHouda BerradaSugandh KumarHouhua LiSugey Sinagawa GarcíaHouliang TangSuguna PerumalHoussem BoulebdSuhail KhanHowaida Abd-AllaSuhail MohdHoward E. KatzSuhaili ShamsiHoyos-Leyva JavierSuhasHristiyan A. AleksandrovSuhyun ParkHristo P. PenchevSui Kiat ChangHrvoj VančikSu-Il InHrvoje PavlovićSuisui HaoHsiaowei LiaoSujan KarHsieh-Fu TsaiSujin LeeHsien-Tsung YaoSukanya SereenonchaiHsin-Chun ChenSukhvinder SinghHsin-Yi HungŞükrü KalaycıHsiu-Mei ChiangSüleyman AşırHsiu-Wen ChienSüleyman Fatih ÖzmenHsuanping ChangSuling HuangHsueh Yun LeeSuman AlishettyHsusheng TsaiSuman ChaudharyHsyueh-Liang WuSuman MukhopadhyayHu LiSuman RanjitHua MenSuman SamantrayHua ShaoSumaporn KasemsumranHua YuSumbal SabaHuai-An ChenSumet KongkiatpaiboonHuaiwen YangSumit Kumar PanjaHuaizhe YuSumit SarkarHuali ZuoSumit Sinha RayHuang Chung-HsiungSun MengtaoHuang QiuSun Young ParkHuan-Ming XiongSunah KimHuanyu JinSuncica BeluhanHua-Sheng ChiuSunčica RocaHua-Tao WuSunday Nwankwo OkaforHuayan YangSung Gu KangHubert ZiółkowskiSung Ho LeeHudson C. PoloniniSung Won KimHugh ByrneSungjin ParkHugo Alexandre Oliveira RochaSungjun JungHugo Ariel AlvarezSungmin KimHugo FragaSung-Woon OnHugo José Nogueira Pedroza Dias MelloSungyul LeeHugo M. OliveiraSunhapas SoodvilaiHugo Morales-RojasSunil K. SharmaHugo Navarro-ContrerasSunil TripathiHugo Pliego-CortésSunisa AkkarasamiyoHui HuangSunny Kumar SharmaHui LuSunshin ChaHui WangSunwoo LeeHui YanSupab ChoopunHui YaoSupriya Shet TilviHui YeSuraj UmdaleHui ZhuSurbhi SharmaHuibin ZouSurender KumarHui-Chi HuangSurender Reddy SalkutiHuie ZhuSurender SinghHuige WeiSuresh ElumalaiHuijing LiSuresh GorleHuijuan ZhengSuresh K. VermaHuilan YueSuresh Kumar KailasaHuilong DongSuresh N. NairHuilong WangSuresh NarvaHuiping ZengSuresh Ponnayyan SulochanaHuiqin WangSuresh TharejaHuisheng WuSuresh V. AmbudkarHui-Woog ChoeSuresh ValiyaveettilHuixian HongSuresh VeeraperumalHuixiao HongSuriya RehmanHuiyu ChenSurjendu BhattacharyyaHui-Yun ChengSurya V. S. R. K PulavartiHulya CakmakSuryadi IsmadjiHumberto BizzoSusai RajendranHumberto De FreitasSusan RichardsonHumberto Hernández SánchezSusan SergeantHumberto PeraltaSusana Anahi DandlenHumberto Rodríguez-RochaSusana IbáñezHung-Chi ChengSusana M. CardosoHung-Chuan PanSusana M. M. LopesHung-Ju LiaoSusana R. SousaHung-Lung ChouSusana RibeiroHung-Tse HuangSusana Rodrigues ChavesHung-Vu TranSusana V. GonzalezHung-Yi ChenSusanne KaeslerHung-Yin LinSusanta Kumar BhuniaHunter MoseleySushant BangruHusam YounesSushil ChanganHusanu ElenaSushil ChaudharyHüseyin CanSushil MiddhaHusni HusinSushma YadavHwajin KimSushmitaa DasHye Jin YouSusmitnarayan ChaudhuryHyeMee KimSusumu KawakamiHyeonggon KangSusumu YanagisawaHyesun KimSutirtha ChowdhuryHyeyoung ShinSuvanjan BhattacharyyaHyoeun ShimSuveen KumarHyoung-Geun KimSuvendu PaulHyuk Soo EunSuvik AssawHyuk-Woo KwonSuvimol CharoensiddhiHyun Joong KimSuwat NananHyun Woo KimSuwimol WongsakulphasatchHyundeog YooSuyong ReHyung Min KimSuzana ApostolovHyung-Sik WonSuzana M. AndradeHyunil JoSuzete Maria CeruttiHyun-Jae ShinSven StadlbauerHyun-Jeong ChoSven T. StrippHyun-Joon HaSvetlana BatashevaHyun-Ouk KimSvetlana BratskayaHyunsik ImSvetlana C. GagievaIan IngramSvetlana GorelovaIan JenkinsSvetlana KamenevaIan Scott RamseySvetlana KlementevaIanasi CatalinSvetlana R. JeremićIbrahim A. NaguibSvetlana SenikIbrahim AbdellahSvetlana SimovaIbrahim Ahmed ShaikhSvetlana SolovievaIbrahim AlghoraibiSvetlana SushkovaIbrahim AlsohaimiSvetlana V. GusakovaIbrahim EissaSvetlana V. SelivanovaIbrahim El-SayedSvetlana V. SmirnovaIbrahim F. RehanSvetlana VorobyovaIbrahim KhalifaSvyatoslav GadomskyIda PalazzoSwapnil SinghIda PoljanšekSwati JainIdan CohenSwati MoreIddo PinkasSwati PundIdoia PostigoSwati SharmaIdolo IfieSwati TanwarIdolo TedescoSwee Keong YeapIdris Adewale AhmedSwelm WagehIdris ArslanSweta C. BalchandaniIdris RajiSyarida Hasnur SafiiIdris YazganSybille HasseIfedayo Victor OgungbeSyed A. A. RizviIffat AhmadSyed A. QuadriIftikhar HussainSyed Ali Raza NaqviIgnacio JessopSyed Amir AshrafIgnacio Peñalosa-CastroSyed Arif Hussain RizviIgnacio Rodríguez-GarcíaSyed Hani AbidiIgnacio Vayá PérezSyed Haris OmarIgnacio VioqueSyed Ibrahim Gnani Peer MohamedIgnacy CukrowskiSyed KhasimIgnasi BarbaSyed Lal BadshahIgnazio BlancoSyed MahmoodIgnazio BruscaSyed Mohd Danish RizviIgnazio RoppoloSyed Muhammad ImranIgor A. FedorovSyed Muhammad UsamaIgor Alexandre Da Silva DiasSyed NabilIgor B. SivaevSyed Najmul Hejaz AzmiIgor G. ZenkevichSyed NaqviIgor GolubSyed Nasir Abbas BukhariIgor IvanovSyed NazreenIgor JakovcevskiSyed Salman ShafqatIgor JerkovićSyed Shams Ul HassanIgor KirilyukSyeda Abida EjazIgor LukićSyeda ShehzadiIgor OpsenicaSylvain AchelleIgor PaštiSylvain CaillolIgor Piotr TurkiewiczSylvester OmoruyiIgor PravstSylvia BoshraIgor PrikhodkoSylvia SoldatouIgor PrudovskySylwester MazurekIgor S. KovalevSylwester ŚlusarczykIgor V. AlabuginSylwia Belica-PachaIgor V. KhudyakovSylwia StudzińskaIgor V. ShishkovskySylwia TyrkalskaIhosvany CampsSylwia Zielińskaİhsan ÇalışSylwia ŻołądekIhsanullah IhsanullahSzabo Zoltan-IstvanIhtisham BukhariSzczepan ZapotocznyIkechukwu P. EjidikeSze Shin LowIkenna AnugwomSzénási TiborIk-Soo LeeSzilvia KlebertIkuo NakanishiSzu-Hao KungIkuya ShibataSzu-Yuan ChenIlaria D’AcquaricaSzymon BocianIlaria De PasqualeSzymon BudaIlaria Elena Lena PalamàSzymon ChorazyIlaria IacobucciSzymon DziombaIlaria SerafiniSzymon ŁośIlario LositoSzymon PlewaIldikó SzabóSzymon SękowskiIldiko SzantoSzymon StarzonekIldinete Silva PereiraT. M. SridharIleana CocanTabassum KhanIleana RãdulescuTabussam TufailIleana RauTada-aki KudoIlenia TinebraTadaaki SatouIlhami YukselTadashi SatohIl-Hoon ChoTadayoshi BesshoIlia IlyinTadesse Fikre TeferraIlian BadjakovTadeusz GóreckiIliana Medina-RamírezTadeusz MalewskiIlija CvijetićTadeusz MuziolIlir ShehuTae Gyu HwangIliyan IvanovTaejong PaikIlker BayerTaekyung KimIllarion DovhyiTae-Woon KimIllimar AltosaarTaha MehanyIlona BednarekTahereh Rohani-BastamiIlona SadokTahir FarooqIlona WandzikTahir RasheedIlsiya M. DavletbaevaTahira PirzadaIlya A. ProkopievTahmid RupamIlya G. ShenderovichTahmineh MokhtariIlya KhodovTai-Chia ChiuIlya KlyukinTaihong LiuIlya Nifant’evTaiki UmezawaIlya ZavodnikTairong KuangImaël Henri Nestor BassoléTaisiya KozlovaIman Dindarloo InalooTaisiya SukhikhIman RoqanTai-Ying ChiouImgon HwangTaiyo YoshiokaImran AliTakaaki HirotsuImran ImranTakafumi ItohImre BakóTakafumi KitazawaImre BoldizsárTakafumi MizushigeImtiaj HasanTakahiko BanIn Jun YangTakahiro ArakawaIn Koo HwangTakahiro KusukawaIna JasutieneTakahiro NagatakeIn-Ah LeeTakahiro YamauchiIndika HerathTakahito OhshiroIndranil ChatterjeeTakakazu MitaniInes BrunoTakanari NakanoInes CastangiaTakao TsunedaInes ELBini-DhouibTakashi HirasawaInès Esma AchouriTakashi IshidaInês GodetTakashi IwasakiInes M. ValenteTakashi KumagaiInes ManciniTakashi KurikiInes NikolićTakashi MatsuoInes PrimožičTakashi MinoInga KwiecienTakashi MisawaInga MatulytėTakashi TanikawaIngmar PerssonTakashi TsunoIngrid CipakovaTakashi WatanabeIngrid Rodríguez-BuenfilTakashi YoshimuraInhee ChoiTakayuki MiyamaeInho NamTakehiro NishidaInmaculada Jénnifer GómezTakehiro YamagishiInna MelnykTakemi OtsukiInna P. KuranovaTakeru ItoInna TolpeshtaTakeshi IshikawaInnokentii E. VishnyakovTakeshi UemuraIn-Soo YoonTaketo YamadaIntan Rosalina SuhitoTakuhiro OtosuIoan StamatinTakuji TanakaIoana CabaTakumi IshizukaIoana GhiutaTakuya KitaokaIoana GomoiuTakuya ShigaIoana Maria CorteaTakuya ShinbayashiIoana StanculescuTal Noy-PoratIoanna NtaikouTalal MallahIoanna-Katerina AggeliTalha Bin EmranIoannis GiavasisTaliane Leila SoaresIoannis KanakisTalib M. AlbyatiIoannis KarabagiasTamaeh Monteiro-AlfredoIoannis KostakisTamaki EndohIoannis KourkoutasTamao IshidaIoannis Mylonas-MargaritisTamar ZelovichIoannis N. TsimpanogiannisTamara I. PajpanovaIoannis NikolakakisTamara JurinaIoannis P. GerothanassisTamara Lazarevic-PastiIoannis PartheniadisTamara LlanoIoannis PetrakisTamara R. TodorovićIoannis S. PappasTamas BanyaszIoannis SavvasTamás HofmannIoannis TsivintzelisTamas KoszegiIoannis VagelasTamás LetohaIoannis ZabetakisTamer ElsakhawyIogann TolbatovTamer M. SakrIolanda De MarcoTamer ShehataIolanda LázaroTamerlan T. MagkoevIolanda Midea CuccoviaTamsyn MontagnonIolanda VendrellTan DunxianIon GrosuTana TandarićIon NedaTanatorn KhotavivattanaIone Parra Barbosa-TessmannTanay KesharwaniIonel MangalagiuTanay KunduIonut LuchianTaner SarIoulia SmonouTangpo YangIppolito CameleTania Maria Grazia SalernoIqrash ShafiqTânia MartinsIram LiaqatTânia MonizIram MurtazaTania PenchevaIrena BarukcicTânia S. MoraisIrena Brčić KaračonjiTaniya PurkaitIrena H. MaliszewskaTanja IlićIrena IvaniševićTanja PušićIrena IvshinaTanja ZidaričIrena MajerzTanmay KulkarniIrena NalepaTanmay SarkarIrena PulkoTanmoy ChakrtbortyIrena RotermanTanmoy SanyalIrene AraTanpreet KaurIrene ChetschikTanushree DangiIrene DiniTanveer Ahmad WaniIrene Gómez-CruzTanveer FaridIrene LingTao FengIrene LorraiTao HuangIrene PanderiTao LanIrene ValasiTao LiIrene VillaTao LiuIreneusz KapustaTao SongIreneusz SowaTao TongIrfan AnsariTao WangIrina A. ZverevaTao YiIrina AnimitsaTao YinIrina D. KonstantinovaTao ZhangIrina FierascuTaotao DaiIrina IelciuTaoufiq BenaliIrina M. Le-DeygenTapan Kumar PalIrina NaletovaTapas ChakrabortyIrina Petrovna BeletskayaTara Chand YadavIrina PopescuTara L. HaasIrina RusakovaTaranpreet KaurIrina TiganovaTarek A. YousefIrina V. KasyanovaTarek MohamedIrina V. SterkhovaTarik A. MohamedIrina YaninaTarik HaritIrina Yu PetrushankoTarik Sqalli HoussainiIrini DoytchinovaTariq AbdulKarim AlalwanIriny AyoubTariq AzizIrlon FerreiraTariq MukhtarIrmanida BatubaraTarique HussainIrshad SharafutdinovTaro UedaIruthayapandi Selestin RajaTarun Kumar DuaIrwin Rose Alencar MenezesTarun Upadhyayİsa SıdırTasuku NakajimaIsaac Uzoma AsuzuTatiana A. KorolenkoIsaac Yves Lopes MacêdoTatiana DomratchevaIsabel AfonsoTatiana GolubevaIsabel MarzoTatiana Maria Souza-MoreiraIsabel RomeroTatiana N. MyasoedovaIsabel S. Naarmann-de VriesTatiana OtroshchenkoIsabel SeiquerTatiana PereiraIsabella PanfoliTatiana S. KalininaIsabella RimoldiTatjana KošmerlIsac Almeida De MedeirosTatjana M. MajkićIsam SalihTatjana P. StanojkovićIsao KadotaTatjana R. StevićIsao MizotaTatjana SimićIsao YamaguchiTatjana ŠoštarićIshtiaque AhmadTatsufumi OkinoIsidora RadulovTatsuhiro HisatsuneIsidro M. PastorTatsuo KandaIslam HusainTatsuo MichiueIslam IbrahimTatsuo NehiraIslam MinisyTatsuro WatanabeIslamiyat Folashade BolarinwaTatsuya MoriyamaIslamy Rahma HutamiTatsuya TakeshitaIsmael León-RiveraTatu KumpulainenIsmael PellejeroTatyana GodovikovaIsmail AlmanassraTatyana PashirovaIsmail Naci CangulTatyana Petrovna KukinaIsmail O. IsholaTatyana Popovaİsmail ÖçsoyTatyana V. AbramovaIsmail YenerTatyana V. VolkovaIsopencu GabrielaTavaszi-Sárosi SzilviaIsrael AlshanskiTawanda ZiningaIsrael Castillo-JuárezTawfik KhattabIsrael SilmanTawheed HashemIsrafil KucukTeerapol SrichanaIssei NakamuraTeerasak E-kobonIstván CsarnovicsTejas BarotIstván FábiánTejas M. DhameliyaIstvan JanossyTejeshwar RaoIstván KonczTejinder PalIstvan NagyTeleky Bernadette-EmokeIstvan PelczerTengfei QiuIstván SzatmáriTengfei SongIstvan ZupkoTeobald KupkaÍtala M. G. MarxTeodor RusuItamar GonçalvesTeodora Emilia ColdeaItzel López-RosasTeodora IvanovaItzik CooperTeodorico C. RamalhoIulia Gabriela DavidTeodorico De Castro RamalhoIulia ZoicasTeoh Seong LinIuliana AproduTeralı KeremIuliana MarinTeresa AdityaIuliana PopescuTeresa Ayora-TalaveraIuliana-Mihaela DeleanuTeresa Del Castillo-SantaellaIunia PodoleanTeresa DiasIurii SemenovTeresa EstevesIva LakićTeresa Fina MastropietroIva PashkulevaTeresa KowalskaIva SovadinovaTeresa MougaIvan A. BoldyrevTeresa OlszewskaIvan A. YaremenkoTeresa RussoIvan BarvikTeresa TropeaIván CarreraTeresa ŻołekIvan D. GrishinTereza KauerovaIvan DimitrovTereza PadrtováIvan DimovTerrone RosenberryIvan I. StoikovTerry RissIvan KodrinTerry Z. H. GaniIvan M. SavicTersilla VirgiliIvan MikheevTeruyoshi TanakaIvan NemecTesfalem WelearegayIvan RajkovicTe-Sheng ChangIván SciscenkoTeshome YehualaeshetIvan ShtepliukTetsuaki FujiharaIvan ŠošaTetsufumi ItoIvan TorresTetsuo IshikawaIvan UzunovTetsuya KonishiIvan V. AnanyevTetsuya MatsukawaIvan V. NechepurenkoTewodros MulugetaIvan VokřálThaigarajan ParumasivamIvan Zapata GonzalezThais Leite NascimentoIvana AleksićThais MilessiIvana BiljanThaíse Gonçalves AraújoIvana CabarkapaThamina ActerIvana CarevThanayut KaewmarayaIvana DrvenicaThangamuthu Mohan DasIvana IvićThangavelu S. RajanIvana MarováThanh Lam NguyenIvana NovakovicThanh Nhut DoIvana PerkovicThanh-Danh NguyenIvana ProdićThanigaivelan ArumughamIvana Savic GajicThapanee SarakonsriIvana ŠkrlecTharun Teja PonduruIvana Škugor RončevićThashree MarimuthuIvana Sredović IgnjatovićThavaree ThilavechIvana StanimirovaTheeraphap ChareonviriyaphapIvana TomacThemistoklis KabanosIvana Z. KuzminacTheocharis KostantinidisIvanka B. SemerdjievaTheodora NikouIvanka SpassovaTheodora PapamitsouIvanka TenevaTheodora-Venera ApostolIvanka TsakovskaTheodore TseliosIvano ViglianteTheodoros VarzakasIvayla DinchevaThi Thao Linh NguyenIvayla Pantcheva-KadrevaThi Thi NgeIvaylo DimitrovThi Tuong Vy PhanIvelina DessevaThiago André Moura VeigaIveta CabalovaThiago Belarmino De SouzaIveta Y. YotovaThiago F. Da ConceicaoIvica AvianiThiago Henrique NapoleãoIvica BlaževićThiago Lopes RochaIvo PiantanidaThibault GallavardinIvona Djurkin KušecThibault ViennetIvor LoncaricThien NguyenIwan ZimmermannThierry AussenacI-Wen SunThierry BessonIwona BryndalThierry MagnaldoIwona Budziak-WieczorekThierry PrangéIwona Cichowska-KopczyńskaThierry RabilloudIwona GabrielThies Henning BüscherIwona KomanieckaThilahgavani NagappanIwona KonopkaThilina U. JayawardenaIwona KowalskaThirugnanasambandan TheivasanthiIwona RutynaThirumaran ThanabaluIwona RykowskaThirupathi RavulaIwona ŚcibiszThiruventhan KarunakaranIwona Wojtasik-KalinowskaThomas BartnikasIwona ZarzykaThomas C. ThannickalIza F. Peréz-RamírezThomas CauchyIzabela Barszczewska-RybarekThomas DippongIzabela GrzegorczykThomas E. KuhlmanIzabela MaduraThomas E. MürdterIzabela Muszalska-KolosThomas EllingtonIzabela Nawrot-HadzikThomas HacklIzabela ŚliwaThomas J. J. MuellerIzabela WysockaThomas J. SimpsonIzabella BrandThomas J. TuckerIzwan BharudinThomas LavergneJ. Michael SorrellThomas LetzelJaber Keyvan RadThomas M. HarrisJace JonesThomas M. KlapötkeJacek Adam SorokaThomas MarshJacek BanaśThomas MavromoustakosJacek GębickiThomas MawhinneyJacek KorchowiecThomas MüllerJacek KujawskiThomas NetticadanJacek ŁyczkoThomas PolasekJacek NowakThomas RuppertJacek NyczThomas ScattolinJacek OwczarekThomas SciorJacek PiosikThomas SeppererJacek SkarżewskiThomas VielJacek SłupskiThorsten KoslowskiJacek W. MorzyckiThuan-Chew TanJacek WawrzykowskiThuc-Huy DuongJacek WojaczyńskiThumnoon NhujakJacinta SerpaThyago QueirozJacob C. LutterTiago A. FernandesJacob Lorenzo-MoralesTiago Bueno MoraesJacobo Hernández-MontelongoTiago CruzJacqueline TembuTiago NazarethJacqueline WiesnerTiago Severo PeixeJacques CurélyTiago VenâncioJacques FantiniTian QiangJacques Romain NjimouTian WangJademilson C. SantosTianliang LuJadwiga Jodynis-LiebertTianlong LiJadwiga ManiewskaTianmiao OuJadwiga SołoduchoTianqiong MaJae Hee ParkTiansheng ZhaoJae Kwang KimTianshi WangJae Won ChangTianyu LiJae Wook LeeTianyu YanJaebong JangTianyuan ChenJaeheung ChoTianyuan ZhangJae-Hong KoTiberiu Paul NeaguJaehong SeoTibo PasinszkiJaemin JeongTibor KibediJaeson CallaTicuta Negreanu-PirjolJaewon JangTie YangJaewoo ChoiTien-Yuan WuJaeyoung KwonTierui ZhangJagabandhu PatraTilen ZamljenJagar A. AliTill OpatzJagdish Kumar JaiswalTillman WeberJagdish SinghTilmann HarderJagoda Kępińska-PacelikTilo LuebkenJagpreet SinghTim HubinJahangir MasudTimir TripathiJai P. RaiTimo SorsaJai PrakashTimofey PylaevJai Singh PatelTimothy D. VeenstraJaime C. GrunlanTimothy HamerlyJaime E. CharrisTimothy J. BoyleJaime EscalanteTimothy J. PriorJaime MataTimothy SnowdenJaime Pérez-VillanuevaTimothy TseJaime PortillaTimur M. MirzoevJaime RodríguezTina KaussJaime Rubio-MartinezTina KeglJaime Santoyo-SalazarTina MaverJaison JeevanandamTina SabelJaker HossainTing HanJakir HossainTingwei RenJakkrawut MaitipTingxiang YanJaksuma PongsetkulTirayut VilaivanJakub DrozakTirtha Das BanerjeeJakub GrzesiakTito R. Sant’Anna Cadaval, Jr.Jakub KaminskyTitus SobischJakub KawalerczykTiziano MarzoJakub KosteckiTiziano TuccinardiJakub M. GacTobias HuefnerJakub MokrzyckiTobias Michael HedisonJakub RokTobias ReiffJakub SzlękTofayel AhmedJakub ZdartaTohru WadaJakub ŻurawskiTo-Hung TsuiJalal GhilaneTokeer AhmedJalal TaneeraTom CollinsJaleel KizhakkayilTom HuxfordJamal HassanTomás Galicia-GarcíaJamal RafiqueTomas HerraizJames A. GreenTomáš KučeraJames A. PlattsTomás Madera-SantanaJames BruceTomas PospisilJames C. WilliamsTomás Rocha-RinzaJames CalvaTomáš TobrmanJames E. Jr. HassellTomas TorrobaJames HarbertsonTomasz BogielJames Kumi-DiakaTomasz CebulakJames La ClairTomasz CzapikJames M. GruschusTomasz DaszkíewiczJames P. C. CoverdaleTomasz DembiczakJames SherwoodTomasz FlorowskiJamshed HaneefTomasz GrabowskiJan AnderssonTomasz GubicaJan BilskiTomasz JelińskiJan BocianowskiTomasz JóźwiakJan BredehöftTomasz KalakJan ChambersTomasz KliśJan FranzTomasz KobielaJan HonzíčekTomasz KoczorowskiJan MirTomasz KosmalskiJan MuschiolTomasz KrawczykJan NisarTomasz KubrakJan NovákTomasz LesiowJan PalackýTomasz LigorJan PetersenTomasz M. KarpińskiJan PetrTomasz MroczekJan S. JaworskiTomasz Norbert KołtunowiczJan SchripsemaTomasz Pawel CzajaJán ŠtěrbaTomasz SawickiJan TkacTomasz SkrzypekJan TrnaTomasz TarkoJan ZidekTomasz WróbelJana CopikovaTomasz ŻelazińskiJana FrankováTomasz ZokJana HeldTomaž PolakJana OklešťkováTomica HrenarJana PiskTominari ChoshiJana Sedlakova-KadukovaTomislav BosiljkovJana SedlaříkováTomislav MašekJana Šic ŽlaburTomislav TostiJana Sopková-De Oliveira SantosTommaso FelicettiJana ŠtefánikováTommaso LomonacoJanah ShayaTomoaki MiyagawaJanakiram R. VangalaTomohiro MizobataJanani ParameswaranTomonari SumiJanar JenisTomonari WakabayashiJandeep SinghTomoya SuzukiJane S. MurrayTomoya YokoyamaJanet R. MorrowTomoyuki MiyaoJanet SoleimannejadTomoyuki MurakamiJanez CerarTong FeiJanez IlašTong WangJanez MavriTongjie LiuJanez MravljakTongliang HuJanina AdamusTongsai JamnongkanJanire Peña-BahamondeTonhajzerová IngridJanja BabičToni GrellJanna ShainskyToni PonsJanne KoivistoTonni Agustiono KurniawanJannis SamiosTony A. RodriguesJános A. OsánTony ReglinskiJanos B. NagyToru ItagakiJános BorbélyToshiaki MuraiJános KissToshiaki NakanoJanusz SmulkoToshiaki TsukudaJanusz ZakrzewskiToshihiko HanaiJapareng LalungToshihiro MurataJaqueline BazioliToshihiro ShimadaJaqueline D. SenraToshimitsu YamaokaJarett WilcoxenToshinori ShimanouchiJarlei FiamonciniToshio NaitoJaromir MichałowiczToshio NorikuraJaroslav FilipToshiro TakaoJaroslav KristofToshishige YamadaJarosław ChojnackiToshitsugu FujitaJarosław ChwastowskiToshiyuki ItohJarosław L. PrzybyłTracy A. BrooksJaroslaw PanekTran Dang XuanJarosław RomańskiTran Duy BinhJarosław WidelskiTran VinhJarosław ŻmudzkiTrapella ClaudioJaroslawa RutkowskaTravis T. DentonJar-Yi HoTrevor ChiwesheJasmina SulejmanovićTrey RonnebaumJasmina VitasTriantafyllos DidangelosJasmine Hadj SaadounTrias ThireouJasminka TalapkoTrina E. TalleiJasna Čanadanović BrunetTrinadh KaicharlaJasna JablanTrinidad Ruiz TéllezJasna Prlić KardumTristan A. ReekieJason A. SmithTristan ZimmermannJason G. TaylorTroy D. WoodJason M. BelitskyTruong Ngoc MinhJason McCallumTruong-Giang VoJason NewtonTruongngoc MinhJason P. SchwansTsan-Chi ChenJason StewartTshimangadzo MunondeJason T. TzenTso-Chou LinJason Thomas DuskeyTsuchida SachioJason W. SidabrasTsukasa IwashinaJatinder SinghTsukui TakayukiJatish A. KumarTsuneaki SakuraiJaume AmengualTsung-Chuan HoJaved AhmadTsung-Han TsaiJavid Ahmad ParrayTsun-Thai ChaiJavier CerezoTsutomu HatanoJavier Eduardo Garcia CastañedaTsutomu InokuchiJavier EspinozaTsuyoshi FukushimaJavier Fernández-RuizTsuyoshi ShiraiJavier Ferreiro-CabelloTsuyoshi YamadaJavier Frontiñán-RubioTsz Him ChowJavier Germán Rodríguez-CarpenaTsz Tin YuJavier González-SálamoTuba BaygarJavier Iglesias-SigüenzaTuba Çiğdem OğuzoğluJavier Murciano-CallesTuba EsatbeyogluJavier OrozTudor Claudiu SpînuJavier PeñaTufan MukhopadhyayJavier Saiz-PoseuTuĝba Kök-TaşJavier Santos-AberturasTugba Taskin-TokJavier SaurinaTuğrul Tolga DemirtaşJavier UrracaTulin ArasogluJavier. CampaniniTullio MonettaJavlon RayimbaevTumey NathanJay BrownTunde AlapiJay P. McLaughlinTung On YauJay TrivediTuo ShaoJayanta Kumar PatraTuomo LaitinenJayaraman MuthukumaranTurgay SeçkinJayaraman SelvarajTushar Kanti DasJayaseelan Benjamin FranklinTuzz-Ying SongJayasri DassharmaTylor LewisJayasudhan Reddy YeraboluTz Chuen JuJayeshbhai ChaudhariTze Ning HiewJaysukh H. MarknaTzi Bun NgJean Marie RuysschaertTzu Hsuan ChiangJean Michel MaixentTzuwei WangJean RodgersUdishnu SanyalJean-Christophe DaigleUgo CarraroJean-Christophe GarriguesUgo CarusoJean-Claude DesfontisUgur CakilciogluJean-Daniel MartyUğur ŞenJean-François HaletUjjwal LayekJean-François HernandezUlf NorinderJean-Luc MontchampUlf PruesseJean-Luc PutauxUlises AmadorJean-Marc CampagneUlises Páramo-GarcíaJean-Marc NuzillardUllrich ScherfJean-Raymond GavarriUlrich FischerJean-Yves Le QuestelUlrich PilatusJeasmin AkterUlrich Severin DischingerJebiti HaribabuUlrich WalterJeelan N. BashaUlrike AlexievJeella AcedoUlrike BrüningJeetendra Kumar GuptaUlrikke VossJeevithan ElangoUma ChatterjeeJeffrey D. EinkaufUma Devi PalanisamyJeffrey LakritzUma K. AryalJekaterina Mazina-ŠinkarUmair KhurshidJelena Antić-StankovićUmair QaziJelena BožunovićUmaporn UawisetwathanaJelena DumancicUmberto BasileJelena GorbatsovaUraiwan PanichJelena KruljUrban BrenJelena LađarevićUrmi RoyJelena MaksimovićUroš ČakarJelena Marković FilipovićUroš M. GašićJelena MilovanovicUroš PecikozaJelena MiocinovicUrsa Pecar FonovicJelena Osmanović BarilarUrška Vrabič BrodnjakJelena PoljarevicUrszula Gawlik-DzikiJelena Popović ĐorđevićUrszula HohmannJelena RoganovicUrszula HubickaJelena T. PetrovićUrszula JankiewiczJelena TamulieneUrszula KarczmarczykJelena TomićUrszula Krupa-KozakJelena TošovićUrszula LisieckaJelena TrifkovićUrszula UlkowskaJelena ViderUsama AwanJelena VladićUsama Ramadan AbdelmohsenJen-Chang YangUsmanJen-Chieh TsaiUtami Dyah SyafitriJen-Chih TsengUtoomporn SurayotJen-Kit TanUtpal NandiJennie M. GardnerUtthapon IssaraJennifer GubitosaUwe BrauchJennifer Martins NoroUwe MonkowiusJennifer MytychÜyesi Hale DemirtepeJennifer R. FlemingUzma HaseenJennifer SchaeferV. L. MangeshJenny DesantisV. ManjuladeviJenny RenautVaclav KasickaJen-Ray ChangVadanasundari VedarethinamJens Laurids SørensenVadde RamuJens TräncknerVadim P. BoyarskiyJeny ShkloverVadivel SethumathavanJeong Eun YooVahid DehghaniJeong HeoVahid FarzanehJeong Tae LeeVahideh RabaniJerald James NairVaibhav A. MantriJer-An LinVaishali AggarwalJeremy TameVaitsis ChristosJer-Ming ChangVaiyapuri Subbarayan PeriasamyJernej StareVakare MerkyteJerome GougeValber PedrosaJerry O. AdeyemiValdir Florencio Da Veiga JuniorJerry ZhuValdir VeigaJer-Yiing HoungValentin A. SemenovJerzy AdamskiValentín HornillosJerzy DátkaValentin RomanovskiJerzy J. LangerValentin V. NovikovJerzy WisniewskiValentin Yu. DoludaJesper T. N. KnijnenburgValentina BoscaroJesse RumboValentina BudaJessica BridouxValentina CitiJessica GluckValentina Di LibertoJéssica Leite GarciaValentina LacivitaJessica LiberlesValentina LatinaJessica MaiuoloValentina MadiaJessie NedrowValentina NigroJesús A. CastroValentina NikolićJesús Adrián LópezValentina PagliaraJesús Antonio Luque-UrrutiaValentina RussoJesús ArrietaValentina SerraJesus Baldenebro-LopezValentina StranieroJesús Fernando Ayala-ZavalaValentina UivarosiJesús Francisco García GavilanValentina UtochnikovaJesús García RubianoValentina ZhurikhinaJesús García-ColungaValentine P. AnanikovJesús Jiménez-RuizValer MicleJesús Manuel De Los SantosValeri V. MossineJesús Marín-SáezValeria AllizondJesús Martín-GilValeria AnconaJesús Mejía-SaavedraValeria CalifanoJesús Muñiz-SoriaValeria D'auriaJesus Olivero-VerbelValéria Regina de Souza MoraesJesús R. ProcopioValeria RighiJesus TresguerresValeria SecchiJesús-María García-MartínezValéria Stefania Lopes-CaitarJeyan MosesValeria TafintsevaJeyashelly AndasValeriano CorbelliniJey-R VenturaValeriano Méndez FuentesJhon Castaño-OsorioValerie CarpenterJhonny Villarroel-RochaValerie E. RymanJhy-Der ChenValerie MeilleJia RongValerie R. WiersmaJia XiongValérie SimonJiachen HuangValerija DunkićJiacheng HeValerio AzzimatoJiafeng DingValerio Flavio GiliJiafu CaoValeriu V. CoteaJiahai ZhangValery ZinchenkoJiahao HuangValeryia KasnerykJiaheng TanVallampati Ramachandra PrasadJiahui ChenValle PalomoJiahui ZhangVan Bon NguyenJiajie LongVan Giau VoJialei DuanVan Khanh NguyenJia-Ling RuanVan-Chuc NguyenJialong JieVanda SerraJian ChenVandana VinayakJian LiVandita KakkarJian LuVanessa CastelliJian PengVanessa MaybeckJian SunVanessa Meier-StephensonJian WangVanessa MoreiraJian YinVanessa RubioJian YuVanessa TagliattiJianan WangVanessa UzonwanneJianbo TongVanessa VieiraJianbo WangVânia Vilas-BoasJiancheng LaiVanish KumarJiandong WuVanisree MulabagalJianfei SunVanja IvkovićJianfeng LiVanja M. TodorovićJiang ZhouVanja ŠeregeljJiangchuan ShenVanja TadićJiangnan PengVan-Nghia NguyenJiangqi WenVanoye LaurentJiangsheng XuVanya KurtevaJiangtao LiuVaromyalin TipmaneeJianguo HuVartika TomarJianguo WangVarun Kumar SinghJianhan ChenVarvara TvorogovaJianhua ZhouVasi Uddin SiddiquiJianhua ZhuVasil SimeonovJianing HanVasile ChisJianing LiVasile LavricJianing MiVasile SimulescuJianlin WuVasilica PopescuJianming KangVasiliki E. FadouloglouJianping CaoVasiliki GkretsiJianqiang XuVasilios AthanasiadisJianxin WangVasilios StavrosJianxiong ZhuVasily SukhorukovJianxun DingVasko JovanovskiJian-Ye ZhangVassilios CharalampakosJian-Yong WuVassilios MyrianthopoulosJianzhang ZhaoVassilis AthanasiadisJianzhong YinVassilis DourtoglouJianzhou CuiVassilis J. DemopoulosJiao ZhaiVassya BankovaJiaqi SuVasudevan ManiJiaqi WangVasyl M. HaramusJiaqiang WangVed Prakash VermaJiawei WangVedrana Čikeš ČulićJiayue GuoVeera KrasnenkoJibo ZhangVeera Venkat Shivaji Rama Rao EdupugantiJichun ZhaoVeeresham CiddiJie AnVelaphi C. ThipeJie DingVelayutham RavichandiranJie FengVelimir MladenovJie Hong ChiangVelma Ganga ReddyJie JiangVelmurugan KrishnasamyJie LiVelyana G. GeorgievaJie ShenVenediktova NatalyaJie ZhangVenelina PopovaJiekai YinVenerando PistaràJierui YuVenkata ChelikaniJihan XiaVenkata Krishna Karthik TangiralaJijin XuVenkata SabbasaniJikai LiuVenkata Surya Kumar ChoutipalliJik-Chang LeongVenkata Surya N. ChavaJilin XuVenkata UdumulaJim Jinn-Chyuan SheuVenkatakrishnan YanamandramJimena MuciñoVenkatesh ChelvamJin Chul YangVenkatesh YarraJin GaoVenugopal Rao SomaJin Hyeok ChaVenugopala Narayanaswamy KatharigattaJin JiaVera A. Vil’Jin LeeVera AlferovaJin WangVera IsaacJin ZhouVera JencovaJinan GuanVera Lucia Marques Da SilvaJin'An WangVera V. BudaevaJinbo FeiVerena PichlerJincai SuVerena SpieglerJinchao LouVerena TaudteJinchen FanVerginica SchröderJin-Chul KimVerónica ManzanoJin-Chung SinVeronica MartiniJincy Parayangattil JyothibasuVerónica Mayela Rivas-GalindoJindrayani Nyoo PutroVerónica Rocío Vásquez-GarzónJiney JoseVeronica TermopoliJing WangVeronika BarišićJing ZengVeronika HuntošováJing ZhaoVeronika KhairullinaJing ZouVeronika Kralj-IglicJingbo PangVeronika MihaylovaJingbo YuVeroniki VidaliJinghua YangVéronique KemmelJingjing GaoVeselin R. MaslakJingming NingVesna JovanovićJingtao BiVesna LazicJingwei WangVesna RadojevićJingxiu YangVesna RastijaJingyi RaoVesna TepavčevićJingyu ZhangVesna Tumbas ŠaponjacJing-Yueh JengVessela Tsakova-StanchevaJing-Yun WuVetriselvan SubramaniyanJinho KimVeymar Guadalupe Tacias-PascacioJin-Hong ShinVibhav GautamJinjie QianVibhor KumarJinjin WangVibhuti AgrahariJinling CaoVicent Sixte Safont VillarrealJinling WangVicente Del AmoJinrong MaVicente Espinosa-SolisJinshan TangVicente Martí-CentellesJinsong HaoVicente Rodríguez GonzálezJinsong ZhangVicente TimónJin-Soo AhnVickram Jeet SinghJin-Soo ParkVictor Alejandro Suarez TorielloJinwei GaoVictor Alves CarneiroJin-Won ParkVictor ChechikJinwu YanVictor DavidJinxin GuoVictor E. Kuz'minJinxin ZhaoVictor F. PlyusninJin-Xun LiuVictor FajardoJinyang ZhuVíctor HernándezJinzhong GuVictor InfanteJiong ChenVictor LeeJiping ChengVictor Lvovich FurerJiquan ZhaoVictor MamaneJirayu TanprasertsukVíctor Manuel García SuárezJiří BarekVíctor Martínez-MerinoJiri KassaVíctor Meza-CarmenJiri PospisilVictor MidlejJitesh BarmanVictor SukhorukovJitlada VichapongVictor UjorJiujiu YuVictor YarzhemskyJiun-Jie ShieVictoria Gómez-MurciaJiuxing JiangVictoria GrishkoJixia WangVictoria KlangJixian ZhangVictoria N. SyryaminaJi-Ye KeeVictoria OsipovaJoachim MüllerVictoria Ramírez GonzálezJoachim ThiemVictoria SalvadoJoan BausellsVictoria V. ShumyantsevaJoan GilVictoria WorkmanJoan M. KingVictoriano MarceloJoan Oñate NarcisoVictorio CadiernoJoana C. BarbosaVictoriya A. GeorgiyantsJoana L. C. SousaVida ŠimatJoana LoureiroVidmantas BendokasJoana MagalhãesViera KaraffovaJoana PintoViet-Khoa Tran-NguyenJoana ValenteViglianisi CaterinaJo-Ann LeongVignesh MuthusamyJoanna Dagmara FedorowiczVignesh SundararajanJoanna DepciuchVignesh ViswanathanJoanna DobrzynskaVijay AvinJoanna FedorowiczVijay KotraJoanna Gdula-ArgasińskaVijay KumarJoanna Gromadzka-OstrowskaVijay Kumar JindalJoanna GrzelczykVijay Kumar SiripuramJoanna HarasymVijaya Anand ArumugamJoanna KarpińskaVijaya Saradhi MettuJoanna KlepackaVijayakumar SukumaranJoanna KozłowskaVijayalakshmi ShankarJoanna KrakowiakVikas KumarJoanna Le Thanh-BlicharzVikas MalikJoanna LeszczyńskaVikash KumarJoanna Małgorzata KrukVikram SarpeJoanna MatysiakVikramjit RatheeJoanna MikloszViktor DrganJoanna MystkowskaViktor Korzhikov-VlakhJoanna OraczViktor MihuczJoanna OrzelViktor PilepićJoanna PagaczViktor ReshetnikovJoanna PodlasińskaViktor TimoshenkoJoanna RaczkowskaViktor V. RevinJoanna RydzViktor ZvarychJoanna SadlejViktória HornokJoanna StefanskaViktoriia V. TorbinaJoanna ZawitkowskaViktorya AviyenteJoannis KallitsisVilena KašubaJoão AmaralVilhelmova-Ilieva NeliJoão Azevedo-SilvaViliana GuglevaJoão BassoVilma KaškonienėJoao Batista RosolemVilma PetrikaitėJoão C. SousaVilma RatautaiteJoão Flávio Da Silveira PetruciVinay RandhawaJoão Guilherme CostaVinayak KhodadeJoão Guilherme De Moraes PontesVinayak UpadhyaJoão Henrique Ghilardi LagoVincent BoudonJoão M. M. RodriguesVincent HucJoão P. Prates RamalhoVincent Kam Wai WongJoão Paulo S. FernandesVincent SallesJoão Paulo WiniarskiVincent SoubannierJoão Pedro VeigaVincent TerrassonJoão PriorVincenzo BrancaleoneJoaquim AlmeidaVincenzo CuomoJoaquim C. G. Esteves Da SilvaVincenzo De LeoJoaquim JaumotVincenzo FerroneJoaquim MarçaloVincenzo GerbiJoaquim Silvério Marques VitalVincenzo MarcotrigianoJoaquín ÁlvarezVincenzo QuagliarielloJoaquin BarberaVincenzo RussoJoaquin BarrosoVincenzo SummaJoaquín María CamposVincenzo TufarelliJochen StrubeVincenzo VaianoJodeh ShehdehVineet KumarJoe PanozzoVineeta RaiJoel LiebmanVinich PromarakJoel T. MagueVinícius OsterneJoelma PerezVinícius R. CamposJoerg ReindersVinit RajJogendra SinghVinita ChittoorJohan E. Ten ElshofVinod BalasubramaniamJohan VandenborreVinod GourJohanna R. BrucknerVinod K. NarayanaJohannes RaffVinod KumarJóhannes ReynissonVinod Kumar PaswanJohannes SchulzeVinod VijayakurupJohannes StadlmannViola CalabròJohan-Owen De CraeneVioleta Alexandra IonJohn ArnasonVioleta DediuJohn ChengVioleta PascalauJohn D. HaleyVioleta PopoviciJohn DespotopulosVioleta ValchevaJohn EnemarkViolina T. AngelovaJohn F. CassidyViorel CircuJohn F. GallagherViorica PârvulescuJohn Kerr WhiteViorica Railean-PlugaruJohn MackVipan KumarJohn MacKayVipin AmoliJohn Man Tak ChuVipin RawatJohn SorensenVirender SinghJohn T. SheridanVirendra YadavJohn ThomasVirgílio De Carvalho D. AnjosJohn VakrosVirgílio FalcoJohn WaltonVirginia Flores-MoralesJohura AnsaryVirginia Moreno-GarcíaJoias HammanVirginia Saez-martinezJoice Tom JobVirginie Lyria LhiaubetJolanta DługaszewskaVirpi VirjamoJolanta Elzbieta MarszalekVishal DwivediJolanta GawalekVishal KachwalJolanta Jaroszuk-ŚcisełVishal KumarJolanta JaśkowskaVishal SharmaJolanta KowalonekVishma Pratap SurJolanta KowalskaVishnu Priya MuraliJolanta MierzejewskaVisnja StepanicJolanta Nawrocka-RutkowskaVisnja StulicJolanta PolakVišnja VrdoljakJolanta RzymowskaVisva Bharati BaruaJolita RadušienėViswanath DasJon P. WoodsViswanathan SajiJonas ContieroVita RudovicaJonas SagboVitaly A. MorozovJonathan BennionVitaly V. KuznetsovJonathan DanielVito CalderoneJonathan K. WilliamsVitor Augusto GarciaJonathan ListVítor SpínolaJonathan PerreaultVittorio ChecchiJonathan Puente-RiveraVittorio PaceJonathan R. DimmockVivek Hamse KameshwarJonathan S. LindseyVivian TullioJonathan SinghViviana MaffeisJonathan SkeltonViviana OnofreiJonathan VennerstromViviana ScognamiglioJonathon Edward MohlViviane De Cássia OliveiraJong Hwa LeeViviane PillaJong Kil LeeVictor I. SaloutinJong Min LeeVlad AzovJong-Dae LeeVlad TomaJong-Eun KimVladena BauerováJonghun LimVladimir A. KomornikovJong-Il ChungVladimir A. KorshunJongmin AhnVladimir A. LarionovJong-Seok KimVladimir A. LazarenkoJong-Tae ParkVladimir A. MitkevichJong-Wha JungVladimir Anatolievich ChistyakovJong-Won KimVladimir B. ShirokovJoo Sung KimVladimir BelotelovJoohee JungVladimir BurilovJoon Ho SeoVladimir DobričićJoonhyuk SuhVladimir DolzhenkoJoon-Young JunVladimir DyakonovJordana GeorginVladimir EgorkinJorddy Neves CruzVladimír FrištákJordi CireraVladimir G. PetrovJordi ErasVladimir GureevJordi FarjasVladimir Gus’kovJordi TeixidóVladimir I. KalininJorens KviesisVladimir I. ZverevJörg KobargVladimir JakovljevicJörg KresslerVladimir K. IvanovJörg PieperVladimir MorochoJörg RadnikVladimir MuronetzJörg ReichenwallnerVladimir P. BarannikovJörg WaglerVladimir P. BerishviliJorge A. LópezVladimir P. ZhdanovJorge Alí-TorresVladimir PalyulinJorge Bañuelos PrietoVladimir PankratovJorge CanhotoVladimir PashtetskiyJorge CervantesVladimír PuchartJorge Cesar MasiniVladimir SapunovJorge Chavez BenavidesVladimir SerezhenkovJorge Del Real-OlveraVladimir ShalgunovJorge EscorihuelaVladimir TihkonovJorge Gonzalez-GarciaVladimir V. BezuglovJorge H. SaavedraVladimir VrkoslavJorge Javier AlfonsoVladimir Ya. LeeJorge L. JiosVladimir YeremeevJorge LopezVladislava GusarJorge Manuel Rosa De MedeirosVojislava BursićJorge Matias-GuiuVojtěch HrdličkaJorge MorgadoVolkan Arif YılmazJorge Olmedo-MartínezVolker ZickermannJorge PereiraVolodja KovalchukJorge RamírezVolodymyr BonJorge SantosVolodymyr M. Gun’koJorge Serment-GuerreroVolodymyr PaukJorge Verdú-AndrésVolodymyr S. BrovaretsJorunn BörveVolodymyr SashukJosé A. CañasVolodymyr V. ZagorodniiJosé A. FigueiraVolodymyyr KravetsJosé A. SaézVrba JiriJosé Agustín Tapia HernándezVuanghao LimJosé Alberto Gil CoriscoVuyisile S. ThibaneJosé Alberto MéndezVytautas MickevičiusJosé Antonio García-LópezWael A. El-SayedJosé Antonio Morales-GonzálezWael ElmoslimanyJosé Antonio PalenzuelaWael HegazyJosé Antonio RodríguezWael MahdiJosé Arturo Olguín-RojasWael TraboulsiJosé Ascención Martínez ÁlvarezWageh-Sobhy DarwishJose BaldoviWai San CheangJosé Baranda RibeiroWajid ZamanJose Basilio HerediaWakako FujitaJosé Blanco-SalasWalaa NegmJose BuesoWalace Do PimJose C. MartinezWaldemar A. MarmisolléJose C. S. CostaWaldemar GoldemanJosé Carlos AndradeWaldemar KazimierczakJosé Carlos Tavares CarvalhoWaldemar KuligJosé Cleiton Sousa Dos SantosWaldemar StamporJose CornejoWaldo CerpaJosé De Jesús Ornelas-PazWalhan AlshaerJosé DíazWalid ElfallehJosé Enrique Barquera-LozadaWalid ZorrigJosé Fernando Huertas-PérezWalied AbdoJosé Francisco Segura PlazaWalied Mohamed AlarifJosé GaviraWalter BergerJosé González-RiveraWalter CabriJosé González-SantamaríaWalter CanonJosé I. BorrellWalter DenJose Javier Miguel-HidalgoWalter J. SalcedoJose Javier Ruiz PerniaWalther BildJosé Jesús Vargas RadilloWan Abd Al-Qadar Imad Bin Wan MohtarJosé JusticiaWan Adibah Wan MahariJosé LeaoWan Hazman DanialJose Luis De PazWan Lutfi Wan JohariJose Luis Marco-ContellesWan Sinn YamJosé Luis PazWanbin ZhangJosé Luis Ruvalcaba-SilWanbing GongJose Luis Valverde PiedraWanfang LiJose Luis Villalpando-AguilarWang LeiJosé Luis Viveros-CeballosWangsa Tirta IsmayaJosé Luiz Martins Do NascimentoWanhao CaiJose M. MirandaWanhao ChenJose M. SansanoWanhe WangJosé Manuel Aguilar GarcíaWanhong MaJosé Manuel BenitoWanlu LiJosé Manuel Botubol-AresWanwan LiJose Manuel Calderon-MontañoWan-Yu TsaiJosé Manuel CruzWaqas AhmadJose Manuel Guevara-VelaWaras NurcholisJose Manuel Martin GarciaWarintorn RuksiriwanichJosé María De La RosaWasan KatipJosé María Palacios-SantanderWaseem AhmedJosé Martínez-LilloWaseem El-HuneidiJosé Miguel P. Ferreira De OliveiraWaseem LoneJosé P. Cerón-CarrascoWasif FarooqJosé P. Da SilvaWasin CharoentanthanakulJosé P. S. AnicetoWasuke MoriJosé Paulo PinheiroWatcharapong MitsuwanJosé Pedraza ChaverriWatshara ShoombuatongJosé Pedro Cerón CarrascoWeaam EbrahimJosé Pérez-RigueiroWee Yin KohJosé PinelaWei ChenJose QuilezWei Chih LinJose R. MoraWei FeiJosé Rafael De AlmeidaWei Guang SeetohJosé Ramón IsasiWei HanJosé Rodríguez-RodríguezWei HengJosé RoperoWei Hsum YapJosé SilvaWei LiJose Sousa CamaraWei NiJosé V. PrataWei ShaoJosé Weverton Almeida BezerraWei ShenJosé X. SoaresWei WuJose-Antonio AyllonWei XiaoJosef JůzaWei XingJosef KameníkWei XuJosef PlanetaWei YangJosefa Isasi MarínWei ZhangJosefina Villanueva RodríguezWei ZhaoJosé-Luis MaldonadoWei ZhengJosemar Gonçalves De Oliveira FilhoWei ZhuJosene Maria ToldoWei-Chao LinJosep AlberoWeichao YangJosep OlivaWeidong XieJoseph Amruthraj NagothWeidong YangJoseph Calvin KouokamWeifang HanJoseph GovanWeifang YuJoseph J. ReczekWei-Feng SunJoseph P. HornakWeiguo TianJoseph P. SteinerWei-Hsiung YangJoseph PesekWeihua ChenJoseph SmilanickWei-Hung ChiangJosephine AlbaWeili KongJoshua TobiasWeilin LiuJosiana A. VazWei-Lun HungJosias MeribWei-Lung TsengJosiel Martins CostaWeimer JörgJosy Anteveli OsajimaWeiping LiJotheeswari KothandaramanWeiqiang PangJovana AjdukovićWeiqun LiuJovana BradicWeiwei HanJovana FrancuzWeiyu JiangJovana KojićWeize WuJovana VundukWen MaJovanny GomezWen ShiJovanov PavleWen ZhangJoy Y. FengWenchao LuJoycy Francely Sampaio Dos SantosWenchao YangJoydeep BhaduryWenchao ZhangJózef DrabowiczWen-Chieh SungJozef HaponiukWen-Dee ChiangJozef KováčikWendy KamanJózsef DeliWenheng ZhangJózsef MihályWen-Hsi ChengJualang Azlan GansauWen-Huang PengJuan A. AyalaWen-Jen LeeJuan Augusto Hernández RiveraWenjia WangJuan Baselga LlidóWenjian LanJuan Bautista De SanctisWenjie RenJuan C. EstevezWenjie WangJuan C. OliverosWenjie ZhouJuan Carlos Alías GallegoWenjin DingJuan Carlos Cuevas BernardinoWenjing MaJuan Carlos Martinez EspinosaWenjing WangJuan Carlos Rendón-AngelesWenjun WangJuan CastilloWenjun ZhangJuan Eduardo Sosa-HernándezWen-Li HsuJuan F. SantibañezWen-Ling ShihJuan Francisco GonzálezWenmiao ChenJuan Francisco MartínWenqing JiangJuan Francisco Rodríguez-LandaWenquan YuJuan FrauWenshan ZhangJuan Gabriel Segovia HernandezWensi TaoJuan Garcıa GuiraoWen-Ta SuJuan Gerardo Reyes-GarcíaWentao LiJuan J. TorradoWen-Tsong HsiehJuan Jose AcevedoWenwei LeiJuan Jose Baeza BaezaWenwu LiJuan José MorenoWenxin FuJuan Jose Nogueira PerezWenxin TongJuan L. BenedéWenxiong ZhangJuan Luis AsensioWenxuan ZhangJuan Luis Pichardo-MolinaWenyuan LiJuan M. GonzalezWenzhou XiangJuan M. Romero-GarcíaWeon Bae KoJuan M. Serrador PeiróWerner BlauJuan Manuel Germán-AcacioWerner SeebacherJuan Manuel GutiérrezWeronika BorymskaJuan Manuel Mejía-AranguréWeronika GonciarzJuan Manuel TiradoWesam S. ShehabJuan P. Palomares-BáezWeslei AmbrósJuan Pablo BortolozziWesley ZandbergJuan Pardo-MonteroWessam H. Abd-elsalamJuan Pedro FernándezWibawa Hendra SaputeraJuan PellicoWidiastuti SetyaningsihJuan PeragónWidya FatriasariJuan Raùl Alvarez-IdaboyWieslaw SwietnickiJuan Rodrigo SalazarWiktor ZierkiewiczJuan RosadoWilfrid BoireauJuan Tolosa BarrileroWill WiseJuan XiongWilliam A. CantaraJuanmei ZhangWilliam B. MillerJuan-Pablo Gomez-EscribanoWilliam BlalockJudit HohmannWilliam ChiappimJudit KrischWilliam DonaldsonJudit S. TóthWilliam G. MayhanJudit VáradiWilliam GriffithsJudith BuchheimWilliam HarrisonJudith MihályWilliam J. TippingJudith MonnierWilliam N. SetzerJudy GopalWilliam PevelerJudyta Cielecka-PiontekWilliam R. KewJue LiuWilliam SganzerlaJuei-Tang ChengWilliam T. BeckJuewen LiuWillian G. BirolliJu-Hye YangWilly BienvenutJui-Cheng ChangWilly CarrasquelJukrapun KomaikulWilman CarrilloJulia D. Toscano-GaribayWilmer PereraJulia LorenzoWilson CardonaJulian A. SchreiberWim BuijsJulian KenyonWin SurachetpongJulian SwierczynskiWing Lueng WongJuliana FariaWing Shing HoJuliana Fonseca De LimaWioleta PietrzakJuliana LunaWioletta DrożdżJuliana Quero ReimãoWioletta ParysJuliana SaínWirginia Kukula-KochJuliane SchuphanWislei R. OsórioJuliane SimmchenWissem Aidi WannesJuliane ViganóWissem MnifJuliano GaravagliaWitold SzczurekJulie BolcaenWładysław G. WieczorekJulie L. SutcliffeWładysław W. KubiakJulien DiharceWladyslaw WeglarzJulien G. MahyWoei Yenn TongJulien LangWojciech BarańskiJulien LegrosWojciech JadwicienczakJulien MassueWojciech Jerzy PietrońJulien PetrignetWojciech KochJulio AlarconWojciech KolanowskiJulio Alberto ZygadloWojciech PiekoszewskiJulio Bastos ArrietaWojciech PlazinskiJulio C. Rivera-LeyvaWojciech SmułekJulio MoralesWolfgang BäumerJúlio Onésio Ferreira MeloWolfgang DomckeJulio Ramirez-CastellanosWolfgang EisenreichJulius LiobikasWolfgang FrenzelJun Cong GeWolfgang FritzscheJun FangWolfgang HornfeckJun HirabayashiWolfgang KandiollerJun LiWolfgang M. SamhaberJun MiyataWolfgang QuappJun Sik LeeWolfgang SipplJun WanWon Y. LeeJun WangWonchung LimJun XiWong Yong FooJun YangWon-Kyung ChoJun ZhaoWon-Sik HanJunaidi KhotibWonwoong LeeJun-Bo ZhangWooli BaeJunfeng MaWoong Sub ByunJung Ji-WonWoonghee LeeJung Yong KimWorawan PanpipatJung-hyun ParkWorawat WattanathanaJung-Rae RhoWöstemeyer JohannesJung-Seok ChoiWouter OlthuisJun-Ho LeeWoźniczka MagdalenaJun-Hui ChoiWufei TangJunhui JiWufu ZhuJunichi FujiiXanthopoulos KonstantinosJun-ichi ItoXavier BrazzolottoJun-ichi KadokawaXavier MartinJunichi SawadaXavier Morato ArusJunjie HaoXavier T. S.Jun-Jun WuXesús FeásJunko NagaiXi ZhaoJunnliang ChangXI ZhouJunrui ChengXia LiJunrui LiXian LuoJunsei TairaXianchuan XieJun-Sheng LinXiang Li (China)Junsoo LeeXiang Li (USA)Jun-Wei ZhaoXiang ShengJunya TanakaXiang WangJunye ChengXiang ZhangJunying MaXiang ZhouJunzeng ZhangXiang-Kai YangJunzhe LouXiangli ZhaoJuozas LazutkaXiangnan LiJuraj MajtanXiangping HuJuraj PiestanskyXiangyu GuJūratė ŠiugždaitėXianjin XuJurca TündeXiankui WeiJurga BūdienėXianshu BaiJürgen GeislerXianwen WangJürgen MartensXiao ChenJurgita ŠvedienėXiao DingJuri TimonenXiao MaJurica NovakXiaobin CuiJurij TronteljXiaobing LuoJuris PrikulisXiaobo HeJürn W. P. SchmelzerXiaochang XueJu-Seop KangXiaodong ChiJussara AmatoXiaodong WangJustas DapkūnasXiaofan JiJustin AntonyXiaofei LiJustyna AgierXiaofeng YeJustyna BrasunXiaohong ZhangJustyna BrzezichaXiaohui LiJustyna GrabskaXiaojia ChenJustyna Krzyżanowska-KowalczykXiaojie LiuJustyna KujawskaXiaoju WangJustyna Mazurek-PopczykXiaojun LiuJustyna MiłekXiaokun OuyangJustyna Piekielna-CiesielskaXiaolei XuJustyna Płotka-WasylkaXiaoling ChenJustyna PyrzanowskaXiaoling MaJustyna Stefanowicz-HajdukXiaoling XuJustyna StrycharzXiaoliu LiJustyna SulejXiaolu LiuJuyoung KimXiaomei HeJyotishkumar ParameswaranpillaiXiaomeng YouK. M. Faridul HasanXiaomin WanK. V. G. Chandra SekharXiaomin XuKabirullah LutfyXiaoming JiangKacper DruzbickiXiaoming LinKadarkaraisamy MariappanXiaoming WangKafeel Ahmad SiddiquiXiaonan XieKah Hui WongXiaoning ZhangKai CaiXiaopeng ChengKai ChenXiaoping PuKai LiXiaoqiang AnKai LichaXiaoqin ZouKai Min YangXiaorong FuKai TaoXiaoshuan ZhangKai WangXiaowei LuoKai ZhangXiaowei OuyangKaihong ChenXiaowei SongKai-Jun LuoXiaoxiong WangKailiang JiaXiaoyan ChenKailin TangXiaoyan YuKaio AleviXiaoyan YuanKaishun BiXiaoying BianKaïss AouadiXiaoyu HaoKaito TakahashiXiaoyu JiKai-Wen ChengXiaoyu WangKaixin LiangXiaoyu ZhangKai-Yin LoXiaoyue ZhuKaiyue ZhangXiaozhong QuKajal GhosalXiaxia DiKakali SenXie BingKakaraparthi KranthirajaXihui BianKakasaheb Ramoo MahadikXijun XuKaKing YanXile HuKalambuka Hudson AngeyoXiming XuKalavathy RajanXin ChenKaleab AsresXin DongKaliannan DurairajXin GuanKalil BernardinoXin LiKalina AlipievaXin LiuKallol PurkaitXin MinKalman ImreXin MuKalukapuge Asanka SanjeewaXin WuKalyan K. DewanXin ZhangKalyan PasunootiXin ZhouKalyan RameshXinfang ZhangKalyana ChakravarthyXing LuKamal Ahmad QureshiXing WangKamal El BissatiXingang YaoKamal KansouXingguo WangKamal KumarXingwei ZhenKamal ShahXingxian ZhangKamalendra SinghXingyun LiKamalesh PasadXinhua GaoKambiz TahvildariXinke ZhangKamel Abd-ElsalamXinlai ChengKamel EidXinping WuKamel LaghmaniXinran LiuKamel MsaadaXinxiang ZhangKamenna VutovaXinyan BiKamil J. KuderXinye LiuKamil JurowskiXinyu LiKamil KaminskiXinyu WangKamil Krzysztof RomanXinzhong ZhangKamil PiwowarekXiongfa YangKamil SokolowskiXiqian JiangKamil SzmucXiumin FuKamil WrobelXiushi YangKamila CzarneckaXiuying YangKamila HrubanovaXiyuan LuKamila KasprzakXiyue ChengKamila KociXiyue ZhangKamila Rybczyńska-TkaczykXochitl Aparicio-FernándezKamlesh BhopaleXóchitl TrujilloKamlesh PrasadXu ChenKampanart HuanbuttaXu Dong ZhangKamran AshrafXu FeiyuKamran T. MahmudovXu FengKan DingXu Ma (China)Kanako KumadaXu Ma (USA)Kanchan BhardwajXu WangKandasamy SaravanakumarXu YangKandasamy SelvamXu ZhangKaneda HirotakaXuan FangKanet WongraveeXubo LuoKang Chiang LiewXucheng HouKang CuiXuchuan JiangKang Mun LeeXudong WangKang WangXue LiKangxu WangXue YongKankan ZhangXuechen LiKannappan ArunachalamXuedan WuKanwal RehmanXuegong SheKapil DevXueguang RenKaran AroraXuejin ZhangKarel D. KlikaXuejun LiKarel Mena-UleciaXueliang ShiKarel SmetanaXuemei XiaoKaren GaudinXueming YangKaren KhachatryanXueqing XuKaren V. Pineda-HidalgoXuesong LiKari L. StoneXuetao XuKarima SchwabXuetong ZhaoKarin Müller-DeckerXuexiang HeKarina SalekXuexiu ZhengKarl BurgessXueying MaKarl StraubXufei WangKarla De Castro Figueiredo BordonXuman ChenKārlis BērziņšXuming ZhuangKarna WijayaXun FengKarnaker TupallyXun SongKarol BulaXuning LiKarol CiepluchXuntian JiangKarol FijałkowskiXupeng HongKarol KyziolXuri HuangKarol SzałowskiXuwei ChenKarol WróblewskiXuwen LiKarola Vorauer-UhlYa LiKarolina A. Wojtunik-KuleszaYachao ZhangKarolina DrężekYacine BoumgharKarolina Kordek-KhalilYadolah GanjkhanlouKarolina KulaYael Diskin-PosnerKarolina LabusYahia Z. HamadaKarolina PavicYahong WuKarolina PyciaYahya SefidbakhtKarolina SłoczyńskaYa-Ju HsiehKarolina StarzakYajuan SuKarolina Szewczyk-GolecYajun LiKarolina SzulcYalda Rahbar SaadatKarolina WieszczyckaYali FengKarsten FleischerYaling HanKarsten GroteYamil LiscanoKarsten MäderYamin BibiKarthick RajanYamin Leprince-WangKarthik KumaraYamin LiKarthikeyan MuthusamyYan DingKarthikeyan SekarYan DuanKarthikeyan VenkatachalamYan JinKartik BeheraYan LiKaruna DixitYan LinKarunamoorthy SaravanakumarYan PengKashif AliYan V. ZubavichusKashif AmeerYan VoloshinKashif ShamimYan WangKashma SharmaYan YangKasturi BanerjeeYan ZhangKatabathini NarasimharaoYan ZouKatalin F. MedzihradszkyYana E. IlievaKatalin LányiYana FeodorovaKatalin UrayYana Roka-MoiiaKatarina D. NovovićYanawath SantaladchaiyakitKatarina ItrićYanbo FangKatarina Lisak JakopovićYanchao WangKatarina M. NikolićYanchuan GuoKatarina SmiljanićYanchuan ZhaoKatarina VukojevicYanfei LiuKatarzyna A. PirógYanfei WangKatarzyna BączekYang JiangKatarzyna Bialik-WąsYang LiKatarzyna Bober-MajnuszYang LiuKatarzyna Gaweł-BębenYang SongKatarzyna GobisYang WangKatarzyna GodlewskaYang YushunKatarzyna Grzelak-BłaszczykYangchun ChengKatarzyna Hnatuszko-KonkaYangfan LuKatarzyna JedynakYang-Ju SonKatarzyna JelonekYangmei ChenKatarzyna Kotwica-MojzychYang-Wei LinKatarzyna KrukiewiczYangxin YuKatarzyna Kucwaj-BryszYangzhen LiuKatarzyna LewandowskaYanhu WangKatarzyna LisowskaYanis Toledano-MagañaKatarzyna MichalskaYanjie ZhangKatarzyna NucYan-Jye ShyongKatarzyna PobiegaYanka KaramalakovaKatarzyna RajkowskaYanli ZhouKatarzyna RatuszYanming XuKatarzyna RoszekYann MolardKatarzyna Smolińska-KempistyYann SarazinKatarzyna Sułkowska-ZiajaYanni ZhaoKatarzyna Sykłowska-BaranekYannick ValléeKatarzyna SzewczykYannis KaramanosKatarzyna SzkolnickaYanping LiuKatarzyna WigluszYanting WangKatarzyna WińskaYanty Noorzianna Abdul ManafKatarzyna Winsz-SzczotkaYanwei LiKatarzyna WittYao WangKatarzyna Wójcik-PszczołaYaochun YuKatarzyna Wolny-KoładkaYao-Haur KuoKatarzyna WozniakYaowared ChulikhitKatarzyna ZawadaYaoyao PengKateřina BišováYaozong LiKateřina DadákováYap Soon PohKatharigatta N. VenugopalaYap-Hang ChanKatharina RentschYaqiong SuKatherine E. VestYara M. MichelacciKathleen FreyYaroslav M. BeltukovKatia BarbaroYash RavalKatia CiuffiYashpal SinghKatia ConceiçãoYasir AkramKatia LiburdiYasir LatifKatia MartinaYasser El-NahhalKatia SeremetaYasser KandilKatiuscia PaganoYasser MajeedKatja KoschorreckYassine Oulad El MajdoubKatragadda Suresh BabuYassine RiadiKatrin FrankeYasufumi FuchiKatrin Schaper-GerhardtYasuhiko KizukaKatrin SchmittYasuhiro KubotaKatrina MirandaYasuhiro NishidaKatsuaki IeguchiYasuhiro NomuraKatsuhito KinoYasuhito KoyamaKatsuya IchinoseYasuhito UezonoKatsuyoshi MatsunamiYasunori YaoitaKaushik GhoseYasuo WatanabeKausik ChakrabortyYasuo YoshimiKaustuv MannaYasushi NumataKaveesha WijesingheYasushi SasajimaKaveh MoulaeeYasuteru ShigetaKavindra Kumar KesariYatao RenKavindra Nath TiwariYaw Duah BoakyeKavita SharmaYaxiong TaoKawee SrikulkitYayoi KawanoKay-Hooi KhooYazan RannehKazem ArzaniYazhong BuKazufumi TakanoYe WangKazuhiko UchidaYean Ling PangKazuhiro HaraguchiYee Ying LeeKazuhiro TamuraYeimmy Peralta-RuizKazuhisa MaedaYekaterina D. BedoshviliKazuhisa WatanabeYen-Hua Crystal HuangKazuhito SuzukiYeon Sun LeeKazunari YoshidaYeou-Lih HuangKazunori MatsuuraYeşim ÖzoǧulKazunori YamadaYesu AddepalliKazuo KobayashiYeung Joon ChoiKazuomi SatoYhiya AmenKazuto TajiriYi HeKazuya KobayashiYi LuKazuya MorimatsuYi PangKe DongYi Sheng LaiKe XiaoYi WangKe XuYibo QiuKebede Taye DestaYi-Cheng ChenKecheng LeiYifan WangKedar N. PrasadYifan ZhuKee-Lung ChangYifei WangKeenan MintzYifeng CaoKee-sung KyungYihang WangKefayat UllahYihang ZhouKei ManabeYihenew Simegniew BirhanKei MaruyamaYihong YeKeigi FujiwaraYi-Huang HsuehKeiji KomatsuYiliang ChenKeiji MurayamaYiming WangKeisuke HagiharaYin WangKeisuke YanagisawaYing GuKeith MurrayYing LiKeith R. FoxYing LiuKeith WarrinerYing LuoKejiang LiYing PanKemal Hüsnü Can BaşerYingang FengKemel ArafetYingang GuiKen LeungYing-Gang LuoKen MillsYinghuai ZhuKen ShiratoYinghui WangKendal RyterYingjie ZhaoKengo HanayaYingxiang LiKeni VidilaserisYingya LiuKenichi GotoYinzhou YanKenichi KimuraYi-Pei LiKenji IkeharaYiqi YehKenji KomaguchiYiqiang LiKenjiro BandowYiran WangKenneth NickersonYiren WangKennio PaimYi-Sheng ChengKenta TeruyaYisong WangKentaro YoshidaYi-Ting ChiangKenzo KoikeYiu-lun TangKerttuli HelariuttaYiwei TangKesaven BhubalanYiwen LiKesawat Mahipal SinghYixuan GuoKeshav KumarYiyuan HanKeumhan NohYngve StenstrømKeunhong JeongYoana KiselovaKeun-Yeong JeongYoann CazaubonKevin ItaYoann LadnerKevin LobbYoav SharoniKevin NashYodthong BaimarkKevin PinneyYogendra Kumar MishraKevin TvrdyYogendra Singh RajpurohitKeyang WuYogesh NarkhedeKeykavous ParangYoh YamamotoKhac-Minh ThaiYohan ChampouretKhac-Uan DoYohei SakaguchiKhadija JavedYoichi SunagawaKhai Pang LeongYolande T. R. ProrogaKhairana HusainYolandy LemmerKhajornsak TragoolpuaYong AiKhaled A. ShaabanYong Chool BooKhaled E. El-KelanyYong DuKhaled ElokelyYong FanKhaled HosnyYong GuoKhaled M. DarwishYong JiangKhaled SalemYong JinKhaleed Abdelsabour ElsayedYong WangKhaleel Ibrahim AssafYong XuKhalid DoudinYong Yoong SoonKhalid ElwakeelYongai XiongKhalid HashimYongbin YanKhalid KarrouchiYongbo XueKhalid Mohammed KhanYongfeng BuKhalid RazaYonghai ChaiKhalil Saad-AllahYonghuan YunKhan KhalidYongjun WeiKhan NaeemYong-Lai FengKhashayar GhandiYonglan LiuKhawar SiddiquiYongming ZhangKhawla S. KhashanYongmun ChoiKhian-Hooi ChewYongping BaoKhizar HayatYongqing ZhangKhursheed ShiekhYongsheng ChenKhurshid AhmadYongsheng ZhaoKhushwant Singh BhullarYongsheng ZhouKi Hyun KimYongxiang SongKi Hyun NamYongzheng XingKianoosh CheghamirzaYoonjee ChaeKianoush Khosravi-DaraniYoon-Seok ChungKiarash Jamshidi GoharriziYoonsoo PangKiat Moon LeeYoon-Yen YowKibeom KimYoshi YamanoKidane GebremariamYoshiharu FujiiKien Voon KongYoshihiko NakataniKieran ScottYoshihisa MatsumotoKiessoun KonatéYoshihito KayakiKihwan ChoiYoshihito SudaKiichirou KoyasuYoshikazu KitanoKijung PaengYoshiki OzawaKi-Kwang OhYoshinao NakagawaKil Sik MinYoshinori MikamiKim Youn-ChulYoshinosuke UsukiKinga AdamenkoYoshio NishimotoKinga GawlińskaYoshiro SuzukiKinga OstrowskaYoshitaka HamashimaKinga SalatYoshitaka HosokawaKinga Stuper-SzablewskaYosif AlmoshariKira V. VyatkinaYosra Ben FadhelKiran LokhandeYosuke GotoKiril B. GavazovYosuke KageshimaKirill EmelyanenkoYosuke ShidaKirill TkachenkoYou HanKirill V. ZaitsevYou ZhouKirill ZuevYouai QiuKirsi Marja Oksman-CaldenteyYoufeng YueKisan ChhetriYouhei MiuraKishneth PalanivelooYoujun XuKishor Kumar SadasivuniYounes HanifehpourKishor MazumderYouness El BakriKishwor PoudelYouness LimamiKíssila RabeloYoung G. ShinKit Leong CheongYoung Hae ChoiKi-Tae HaYoung Ho KohKittibunchakul SuwapatYoung Il JeongKi-Yeon YooYoung Joon SungKiyonori KawasakiYoung Sam KeumKiyoshi FujisawaYoung Sik KimKiyoshi TanemuraYoung Whan ChoiKi-Young KimYoung Wook LeeKiyoyuki KitaichiYoung-Dae GongKlára KosováYoungeup JinKlara RetkoYoung-Geun ParkKlaudia KaniewskaYounghoon JangKlaudia KocurekYoungjoo KwonKlaus DollYoungmin LeeKlaus H. HoffmannYoung-Mo KimKlaus HeeseYounhee KoKlaus PetryYoun-Suk ChoiKlaus UnfriedYousef AlthobaitiKléber T. De OliveiraYousry BayoumiKlodian XhanariYoussef AttiaKo FujimoriYoussef ElamineKoh SugamataYoussef RouphaelKoh TakeuchiYoussef W. NaguibKohei KawabataYou-Ying ChauKoichiro WadaYouzhi TangKoji AoyamaYouzhong LiuKoji NakanoYu CaiKoji OohoraYu ChenKojima TatsuhiroYu H. SunKollarigowda RavichandranYu LiuKollur Shiva PrasadYu MengKonda Mani SaravananYu TangKonda Reddy KarnatiYu XiaoKondapa Naidu BobbaYu ZhangKong Shun Ah-HenYu ZhaoKonrad A. SzychowskiYu ZhouKonrad KowalskiYuan HuKonrad RudnickiYuan NieKonstantin ChekanovYuan YuKonstantin DomasevitchYuanchao PeiKonstantin Ivanov HadjiivanovYuanhua SangKonstantin NikolaevYuan-Min ShenKonstantin S. RodyginYuan-Pin ChangKonstantin V. KiselevYuanshuai LiuKonstantin V. KudryavtsevYuanwei ZhangKonstantin V. MoiseenkoYuanyuan ShangKonstantin VolchoYuanzhi ChengKonstantina YannakopoulouYu-Che LinKonstantinos ChristoforidisYu-Chen LoKonstantinos DimasYu-Chia ChangKonstantinos GardikisYu-Chun ChiangKonstantinos GkrintzalisYudi RosandiKonstantinos LiarasYudong ShenKonstantinos SimeonidisYudong WangKonstanty RutkowskiYue GuiKorawan SringarmYue HuKorey CarterYue JiangKorzec MateuszYue SuKostas BethanisYue Wang (Australia)Kostas KasiotisYue Wang (China)Kosuke MinamiYuemin BianKosuke NishiYueqin George ZhengKosuke OkuyaYuerou ZhangKotai LaszloYueshen WuKouhei YamadaYuesheng LiKowalska MałgorzataYue-Wen ChenKowit HengphasatpornYufeng QinKranthiraja KakaraparthiYufeng XiaoKrastena NikolovaYugal Kishore MohantaKravchenko YaroslavYugang ZhaoKrešimir SalamonYuhao LiKrijt JanYuhong LiuKripa ShankarYuhong ZhengKrishna SharmaYuh-Shuen ChenKrishnan ThirumoorthyYuhsuan TsaiKrishnarjuna BankalaYuichiro KobayashiKristen Lynn KozielskiYuji KajitaKristian LeisegangYuji MikataKristian PastorYuji ShimizuKristina KukurovaYujia WangKristina LožienėYujie QiangKristina PavicYuki HitoraKristina Pogrmic-MajkicYuki NakagawaKristina RadićYukinori YabutaKristina RadoševićYuksel AkinayKristina RamanauskienėYukui RuiKristina SepcicYulia A. FrankKristl JanjaYulia BaburinaKristyán SándorYulia BudnikovaKrisztián HorváthYulia GorbunovaKrisztina BelaYulia I. DeryabinaKrisztina LudányiYulia KudyakovaKrisztina NikovicsYulian TumbarskiKrystyna GizaYulian VoynikovKrystyna Mazan-MamczarzYuliana Nancy DewiKrystyna MichalakYu-Liang YangKrystyna OlczykYuliar FirdausKrystyna Skalicka-WoźniakYuliia S. SidorovaKrystyna Szymandera-BuszkaYulin HuKrzesimir CiuraYulin RenKrzysztof Artur BogdanowiczYulin TangKrzysztof B. BećYuliya E. RyzhkovaKrzysztof BienkowskiYuliya Yu. TitovaKrzysztof BiernackiYuljae ChoKrzysztof BrzezińskiYulong HuangKrzysztof DołowyYulong ZhongKrzysztof DziedzicYu-Lun ChangKrzysztof GibasiewiczYumin DaiKrzysztof KruczalaYun ChiKrzysztof KucińskiYun LiuKrzysztof MarciniecYun ShiKrzysztof MudrykYun-Bao LiuKrzysztof NawaraYun-Chin ChungKrzysztof OwsianikYung Bum SeoKrzysztof PolewskiYung Hou WongKrzysztof SzczepanowiczYung-Chia ChenKrzysztof SztankeYung-Chuan LiuKrzysztof WalczyńskiYungyi ChengKrzysztof WaleronYunhi ChoKrzysztof WieczorekYunhui GeKrzysztof ŻamojćYunhui XuKrzysztof ZborowskiYunierkis Perez CastilloKrzyżak EdwardYu-Ning TengKsenia ParchomenkoYun-Kee JoKsenija DurgoYunlan SuKseniya MaryuninaYunlong ShiKseniya V. BelyaevaYunqian DaiKu Youn BaikYunusa UmarKuang-Sheng YehYunwei NiuKuan-Han LeeYunwen TaoKubinec RobertYunxiao ZhangKubo KazuyaYunyan DengKuei-Hung LaiYunzi FengKui ChengYupeng ZhouKuiwu WangYuqing ZhangKuldeep BansalYuri KimKumar Ashutosh Sri HariYuri NascimentoKumar GanesanYuri NesmelovKumar KatraguntaYuri V. PisarevskyKumar VaibhavYurii KistenevKumar VikrantYurii ShepelytskyiKumaran KadirgamaYuriy G. DenisenkoKumaran VediappanYuriy LittiKumiya SugiyamaYuriy MalyarKummari Subba Venkata Krishna RaoYuriy ShulgaKun LiangYuriy Yu. RusakovKun LiuYuriy ZhabanovKun TianYury BelousovKun ZhouYury TorubaevKunal NepaliYusaku MiyamaeKunal RoyYushe YangKunle OkaiyetoYusof KamisahKun-Ling TsaiYusuf Can GerçekKunlun HongYusuf HaggagKunqi ChenYusuf SertKunyan ZhangYusuf TutarKuo Hao LeeYusuf Valentino KanetiKuo-Chang LuYusuke YamamotoKuo-Shyan LinYuta MatsushimaKupka TeobaldYutaka InoueKvěta KalíkováYutaka MatsudaKwang Suk LimYutaka OhsedoKwang-Mook JungYutaka TakaguchiKyeongryoon LeeYutaka TamaruKyle R. OverdeepYutao HuoKyoji MoritaYuthana PhimolsiripolKyosuke IsodaYutian MaKyoungJoo ChoYuto MoritakeKyriakos ProusisYuttapoom PuttisongKyuichi YasuiYuuki HataKyungil KongYuvaraj ArunKyung-Mok ParkYu-Wei ChangLa Hoang AnhYu-Wei ChengLacrimioara SenilaYu-Wu ZhongLada Lukić-BilelaYuyin ZhouLadislav Eav KokoškaYuyun LuLaetitia L. S. Canabady-RochelleYuzhi XuLahcen DaoudiYuzhu LiuLahcen KhouchafYves QueneauLahcen OuahmaneYves RoggoLahoucine BahsisYves-Jacques SchneiderLaia Josa-CulleréZaara SarwarLaibao ZhangZacharoula IatridiLaifeng LuZachary GatesLaila Muñoz-CastellanosZachary SmithLaila NoureenZachary T. GraberLaima ČesonienėZafar Hussain IbhupotoLajos Attila PappZafer CeylanLak Shin JeongZaheeruddin MohammedLakmini SenavirathnaZaher JudehLakshmanan GovindanZahid MajeedLakshmi Narashimhan RamanaZahid ShafiqLakshmi Priya DattaZahra DehghaniLalit BatraZahra EsfandiariLalit GolaniZahra GharariLalitha GummidiZahra LotfollahiLamar O. MairZaid MaayahLamees HegazyZaid YounesLamprini MalletzidouZaihua DuanLan LiuZaiming ChenLan ZhangZain Ul-AbdinLanderos-Martinez Linda-LucilaZaizhu LouLara K. AbramowitzZakaria HafidiLara Saftić MartinovićZakaria TagnamasLarisa A. TsarkovaZamantha Escobedo-AvellanedaLarisa TsarkovaZaneta Czyznikowska-BalcerakLarisa VolovaZanmin HuLars KattnerZarenezhad ElhamLars Oliver KlotzZarezade VahidLars Porskjær ChristensenŽarko MitićLars-Peter KamolzZarrin BasharatLászló AlmásyZbârcea Cristina ElenaLászló HazaiZbigniew AdamskiLászló JicsinszkyZbigniew CzarnockiLászló PusztaiZbigniew DutkiewiczLászló SiposZbigniew J. WitczakLászló SomsákZbigniew KarczmarzykLaszlo SzalayZbigniew PedzichLaszlo WojnárovitsZbigniew SrokaLászló ZimányiZbigniew WitczakLatha PeriyasamyZbigniew ZiembikLatifa BouissaneZbyšek PoselLato PezoZdenek RemesLauberts MarisZdenek StaryLaura Antonella AronicaZdeněk TůmaLaura BulgariuZdenek WimmerLaura Buzón DuránZdenka FohlerováLaura C. J. PereiraZeba FarooquiLaura Carmen ApostolZebin GuoLaura De LellisZeeshan AjmalLaura De PlanoZeeshan HamidLaura Dorina DinuZeeshan RafiLaura EstévezZeev PoratLaura FerrerZehra EdisLaura Grațiela VicașZehua LiuLaura GutiérrezZeineb AturkiLaura HondebrinkZeineen MominLaura IeloZekai ZhangLaura Leticia Barrera-NechaZeliha DemirelLaura LombaZeliha SelamoğluLaura Lõpez-PingarrõnŽelimir JelčićLaura MartínZeljan MalesLaura OrianŽeljana FredotovićLaura Rodríguez-PérezŽeljka PeršurićLaura RombolàŽeljko AndabakaLaura Rubio LareuZeljko KnezLaura SbarceaZenghui DiaoLaura ScranoZenghui LiuLaura SiracusaZengqian ShiLaura StanZening WangLaura TiberioZenon WęglarzLaura TomaZeping WangLaura ValdezZeshan AliLaura XicotaZeyan LiLaurencas RaslavičiusZeynel SeferoğluLaurence DinanZeynep Tacer CabaLaurence DujourdyZhandos TauanovLaurent ChiarelliZhang LiLaurent DufosséZhaofeng WuLaurent M. SachsZhaohai QinLaurentiu RusenZhaoqi ZhangLauro Velazquez-SalinasZhaoqin WangLaurye Van MaeleZhaoxi SunLauryna PudziuvelyteZhaoxiong WanLavinia ArdeleanZhe LiuLavinia MorosiZhefei PanLawrence Asamoah AdutwumZhen ChengLawrence M. WolfZhen JiangLaxman MainaliZhenbin ZhangLe Minh Tu PhanZheng ChenLe ZhaoZheng ChengLeandro Augusto CalixtoZheng GaoLeandro MamoneZhengbao ZhaLeandro PrevostoZhenghan WangLeandro Wang HantaoZhenglin YangLeandro ZúñigaZhengming QianLebaka Veeranjaneya A. ReddyZhengnan YuanLeda GuzmánZhengnian LiLee Seong WeiZhengtao XiaoLee Te ChuanZhengwei HuangLee Wei LimZhengyi HuLeena KhannaZhengyun WuLei BaoZhengze PanLei GuoZhenhua ZhangLei HuangZhenhuan ZhaoLei LiZhenjia ZhengLei LiuZhenmin HongLei LuoZhenning YanLei MaZhenwen ZhaoLei MeiZhenxin WangLei ShaoZhenxing WangLei WangZhenyuan WangLei WuZhexenbek ToktarbayLei XuZhi ChangLei YinZhi ChaoLei ZhangZhi YangLeila A. ChiavacciZhi YueLeila KeshavarzZhichao LouLeili MohammadiZhichong QiLeilson RibeiroZhichuan ZhuLeiting ShenZhifei YanLekshmi R. NathZhifeng JingLélia ChambelZhifeng XinLena RuzikZhigang LyuLenka CeslovaZhigang XuLenuta KloetzerZhigang YinLenuta-Maria SutaZhigang ZangLeo FrkanecZhihao CuiLeonard AtanaseZhiheng HuangLeonard SiebertZhihui ZengLeonardo M. TurchenZhijie TanLeonel Paredes-MadridZhijun WangLeonel PereiraZhijun ZhangLeonid A. KulikovZhikan YaoLeonid B. KrivdinZhipeng WuLeonid FershtatZhipeng ZhangLeonid G. GorbZhiping LiuLeonid M. KustovZhiqi HeLeonid V. KulikZhiqiang FanLeonid V. PerelomovZhiqiang NiuLeontina C. GurguZhiqing LiuLeqi CuiZhirong ChenLesław JuszczakZhirong LiuLeszek KadzińskiZhi-Ting YeLeszek SzablewskiZhiwei WenLeticia González-MayaZhiwei YangLeticia Xochitl Lopez-MartinezZhiwei YeLetizia BonizzoniZhixiang ZhangLetizia CrocettiZhiyong HeLetizia SavioZhiyu WangLevan ChkhartishviliZhiyuan FengLevent AydemirZhiyuan WuLevent PelitZhiyue ZhangLewis Kirk PennyZhizhi DuLeyao ShenZhongbing LuLeyre MadariagaZhonggang FengLi FuZhongqi LiuLi LiZhongtao DingLi LuZhongwu ZhouLi WangZhongxing JiangLi WeiZhongyi MaLi WuZhou HuiLi XiangjunZhousheng XiaoLi Zhang (China)Zhuangrong HuangLi Zhang (USA)Zhuo ChenLi ZhengZhuo ShangLia Tânia J. DinisZhuo WangLian LiZhuoran MaLiana Claudia SalanţăZhuoyan HuLianet MonzoteZia Shariat-MadarLiang LiZia Ullah KhanLiang ZhaoZiad MoussaLiang ZhouZiaudding KhanLiangchun LiZichao WangLiangfeng HuangZidan ZhangLiang-Yu ChenZienab F. R. AhmedLibo ZhangZifan WanLibor BrabecZihao OuLibor KostkaZijian LiLichen BaiZijian YaoLicia PantanoZijian ZhangLidia CicconeZili ZhaiLidia GaffkeZinaida B. ShifrinaLidia MonteroZiqing ChenLídia PinheiroŽivoslav TešićLidia Ruiz-RoldánZiwen WangLidia Stasiak-RóżańskaZixin ChenLidia Szulc-DąbrowskaZizy Ibrahim ElbialyLidiia KolzunovaZlata Kelar Tučeková TučekováLidija Androš DubrajaZlata KralikLidija BarišićZoe ToddLidija BegovićZofia Hordyjewicz-BaranLidong WuZofia MazerskaLifeng ZhuZofia Urbanczyk-LipkowskaLigen LinZoi G. LadaLígia Carla Faccin GalhardiZoltan BozokiLígia MartinsZoltán DudásLígia Nunes De Morais RibeiroZoltán KókaiLigia ValenteZoltán SzűcsLi-Han ChenZoltan UjhelyiLihong LiZoltán VargaLi-Hsin ChanZongkui WangLihua WangZongliang LiuLijiang XuanZongshuai ZhuLijiang YangZongtao LinLijing FangZoran JovovicLijuan XieZoran MarkovićLi-Jun ChenZoran RadicLik Tong TanZoran ZekovićLikang ChinZorica StojanovićLi-Kwan ChangZorita DiaconeasaLili FengZrinka MihaljevićLili HuangZrinka RajićLili LiZsanett S. BodorLili ZhangZsolt FabianLilia CroitorZsolt GállLiliana CepoiZsolt KelemenLiliana DobrzanskaZsolt SzakonyiLiliana GrenhoZsuzsanna Bata-CsorgoLiliana LiceaZsuzsanna GáborikLiliana Mititelu TartauZsuzsanna LászlóLiliana Ostopovici-HalipZuankai WangLiliana RockenbachZuhai LeiLiliane Guerlou-DemourguesZuhair JamainLilianne BeolaZunyao WangLilibeth Salvador-ReyesZuoquan JiangLiliya A. PasechnikZupeng ChenLiliya FrolovaZurina HassanLiliya S. LogoydaZuzanna BielanLillian GungatZuzanna RzepkaLilya DzhemilevaZvezdelina L. YanevaLiming WangZygfryd WitkiewiczLiming WuZygmunt WarzechaLiming Yang



